# Peripheral Nerve Single-Cell Analysis Identifies Mesenchymal Ligands that Promote Axonal Growth

**DOI:** 10.1523/ENEURO.0066-20.2020

**Published:** 2020-06-11

**Authors:** Jeremy S. Toma, Konstantina Karamboulas, Matthew J. Carr, Adelaida Kolaj, Scott A. Yuzwa, Neemat Mahmud, Mekayla A. Storer, David R. Kaplan, Freda D. Miller

**Affiliations:** 1 Program in Neurosciences and Mental Health, Hospital for Sick Children, 555 University Avenue, Toronto, Ontario M5G 1X8, Canada; 2 Institute of Medical Sciences University of Toronto, Toronto, Ontario M5G 1A8, Canada; 3 Department of Physiology, University of Toronto, Toronto, Ontario M5G 1A8, Canada; 4 Department of Molecular Genetics, University of Toronto, Toronto, Ontario M5G 1A8, Canada

**Keywords:** growth factor, nerve, paracrine interactions, regeneration, scRNA-seq, peripheral neurons, neuronal growth, Schwann cell, mesenchymal cell

## Abstract

Peripheral nerves provide a supportive growth environment for developing and regenerating axons and are essential for maintenance and repair of many non-neural tissues. This capacity has largely been ascribed to paracrine factors secreted by nerve-resident Schwann cells. Here, we used single-cell transcriptional profiling to identify ligands made by different injured rodent nerve cell types and have combined this with cell-surface mass spectrometry to computationally model potential paracrine interactions with peripheral neurons. These analyses show that peripheral nerves make many ligands predicted to act on peripheral and CNS neurons, including known and previously uncharacterized ligands. While Schwann cells are an important ligand source within injured nerves, more than half of the predicted ligands are made by nerve-resident mesenchymal cells, including the endoneurial cells most closely associated with peripheral axons. At least three of these mesenchymal ligands, ANGPT1, CCL11, and VEGFC, promote growth when locally applied on sympathetic axons. These data therefore identify an unexpected paracrine role for nerve mesenchymal cells and suggest that multiple cell types contribute to creating a highly pro-growth environment for peripheral axons.

## Significance Statement

This work expands our understanding of the cellular sources of ligands in the injured peripheral nerve that are potentially important for promoting axon growth. Here, we used single-cell RNA sequencing (scRNA-seq) to reveal that Schwann cells and, surprisingly, nerve mesenchymal cells are primary sources of ligands in the injured nerve. We then combined injured nerve scRNA-seq data with proteomic and transcriptomic data from sensory and sympathetic neurons and used a systems-biology/modeling approach to predict novel mesenchymal cell-derived factors that may promote peripheral axon growth. We tested some of these predictions and found three factors, ANGPT1, CCL11, and VEGFC, that promoted outgrowth of cultured sympathetic axons, supporting a potential role for mesenchymal-derived factors in axon growth.

## Introduction

Following injury, mammalian peripheral neurons can regenerate and reinnervate their target tissues. Their ability to do so is thought to be a consequence of a peripheral nerve environment that is highly supportive of axonal growth. Support for this idea comes from classic studies with CNS neurons, which normally fail to regenerate following brain or spinal cord injury but will regrow their axons when peripheral nerve segments are transplanted into the damaged region (David and Aguayo, 1981; for review, see Benowitz et al., 2017). Intriguingly, peripheral nerves are also important for maintenance, repair and regeneration of the non-neural tissues that they innervate. For example, normal peripheral innervation is essential for mammalian hair follicle and hematopoietic stem cells (Brownell et al., 2011; Yamazaki et al., 2011), for cardiac and dermal repair (Mahmoud et al., 2015; Johnston et al., 2013, 2016) and for amphibian limb (for review, see Kumar and Brockes, 2012) and murine digit tip regeneration (Johnston et al., 2016).

The supportive peripheral nerve environment has largely been ascribed to growth factors made by nerve cells (for review, see Terenghi, 1999; Fledrich et al., 2019). These nerve-derived ligands have been particularly well studied with regard to axonal development and regeneration (Chen et al., 2007; Fledrich et al., 2019), although several studies have shown that they are also important for limb and digit tip regeneration (Kumar et al., 2007; Johnston et al., 2016). These growth factors are thought to be Schwann cell derived, since transplantation of Schwann cells alone is enough to promote CNS axon regeneration (for review, see Bunge, 2016) and murine digit tip regeneration (Johnston et al., 2016). In addition to growth factors, the peripheral nerve provides an extracellular matrix environment that is highly conducive to axonal growth, particularly by contrast to the CNS, where known axon growth inhibitors prevail (Chen et al., 2007; Benowitz et al., 2017). This supportive substrate is also thought to derive in part from Schwann cells, which generate a basal lamina and synthesize ECM proteins and cell adhesion molecules (Muir, 2010; Gardiner, 2011).

These studies all indicate that Schwann cells play an important role in establishing a nerve environment that is supportive of axonal growth. However, the nerve is a structurally-complex tissue containing many different cell types, including vasculature-associated cells, immune cells such as tissue-resident macrophages, and mesenchymal cells of both mesodermal and neural crest origin. In this regard, one recent study identified four transcriptionally and spatially-distinct populations of *Pdgfra*-positive mesenchymal cells within the injured peripheral nerve, including endoneurial mesenchymal cells that are tightly associated with Schwann cells and axons (Carr et al., 2019). These nerve mesenchymal cells were shown to directly contribute to the repair and regeneration of mesenchymal target tissues including the digit tip, bone, and dermis. Nerve mesenchymal cells have also been shown to play an essential role in forming bridges over gaps in injured nerves (for review, see Cattin and Lloyd, 2016). Together, these findings raise the possibility that mesenchymal cells might also be important for axonal growth in the peripheral nerve.

Here, we provide support for this concept, using an unbiased systems biology approach to define the sciatic nerve ligand environment. We show, using single-cell profiling, that under both homeostatic and injury conditions, mesenchymal cells and Schwann cells are the predominant sources of peripheral nerve ligands, including known and uncharacterized ligands, and that there is induction of ligand expression in both these cell types following injury. Moreover, using mass spectrometry, transcriptional profiling, and computational modeling, we show that peripheral neurons and CNS retinal ganglion neurons express receptors for many of these ligands. Finally, we validate three of these ligands, ANGPT1, CCL11, and VEGFC, as being synthesized and secreted by *Pdgfra*-positive nerve mesenchymal cells and show that they can promote growth when applied to axons of peripheral sympathetic neurons. Thus, our data support a model where nerve mesenchymal cells and Schwann cells collaborate to establish a generally supportive growth environment in the peripheral nerve.

## Materials and Methods

### Animals

All animal procedures were performed in accordance with Canadian Council on Animal Care regulations as approved by the Hospital for Sick Children animal care committee. Sprague Dawley rats (purchased from Charles River) used in this study ranged from embryonic day (E)15 to young adult (six weeks old) and CD1 mice (purchased from Charles River) ranged in age from eight to twelve weeks old. All rats and mice were healthy throughout the duration of the study and had free access to chow and water in a 12/12 h light/dark cycle room. In most cases, rats and mice of both sexes were used with the exception of six-week-old male rats for sciatic nerve injury microarray experiments. *Pdgfra^EGFP/+^* (B6.129S4-Pdgfrα*^tm11(EGFP)Sor^*/J; JAX stock #007669; Hamilton et al., 2003) mice were obtained from The Jackson Laboratory and were bred and genotyped as recommended by The Jackson Laboratory. Animals that underwent sciatic nerve injury surgeries were housed individually for recovery purposes.

### Sciatic nerve resection surgeries

Sciatic nerve resections were performed on young adult male Sprague Dawley rats (microarray analysis), adult CD1 mice (scRNA-seq analysis) or adult *Pdgfra^EGFP/+^* mice [fluorescence *in situ* hybridization (FISH) and immunostaining]. Before surgery, animals were anesthetized with 2% isoflurane gas and the surgical site was shaved. Animals were kept under anesthesia for the duration of the surgery. To resect the sciatic nerve, an incision was made along the lateral aspect of the mid-thigh of the right hindlimb, the sciatic nerve was then raised, an ∼5- to 10-mm segment was removed, and the distal nerve ending was carefully tucked away (distally) from the injury site to prevent regeneration. The wound was then closed with 4–0 Polysorb sutures (Covidien). Animals were treated subcutaneously with ketoprofen or meloxicam (∼2–5 mg/kg) as well as buprenorphine (0.05 mg/kg) before surgery, along with a postoperative treatment of ketoprofen or meloxicam 24 h after surgery. Mice and rats were housed separately following surgery and remained healthy throughout the postoperative period and were monitored twice daily for 3 d following surgery.

### Single-cell isolation and myelin removal for Drop-seq analysis

**Figure 1.**
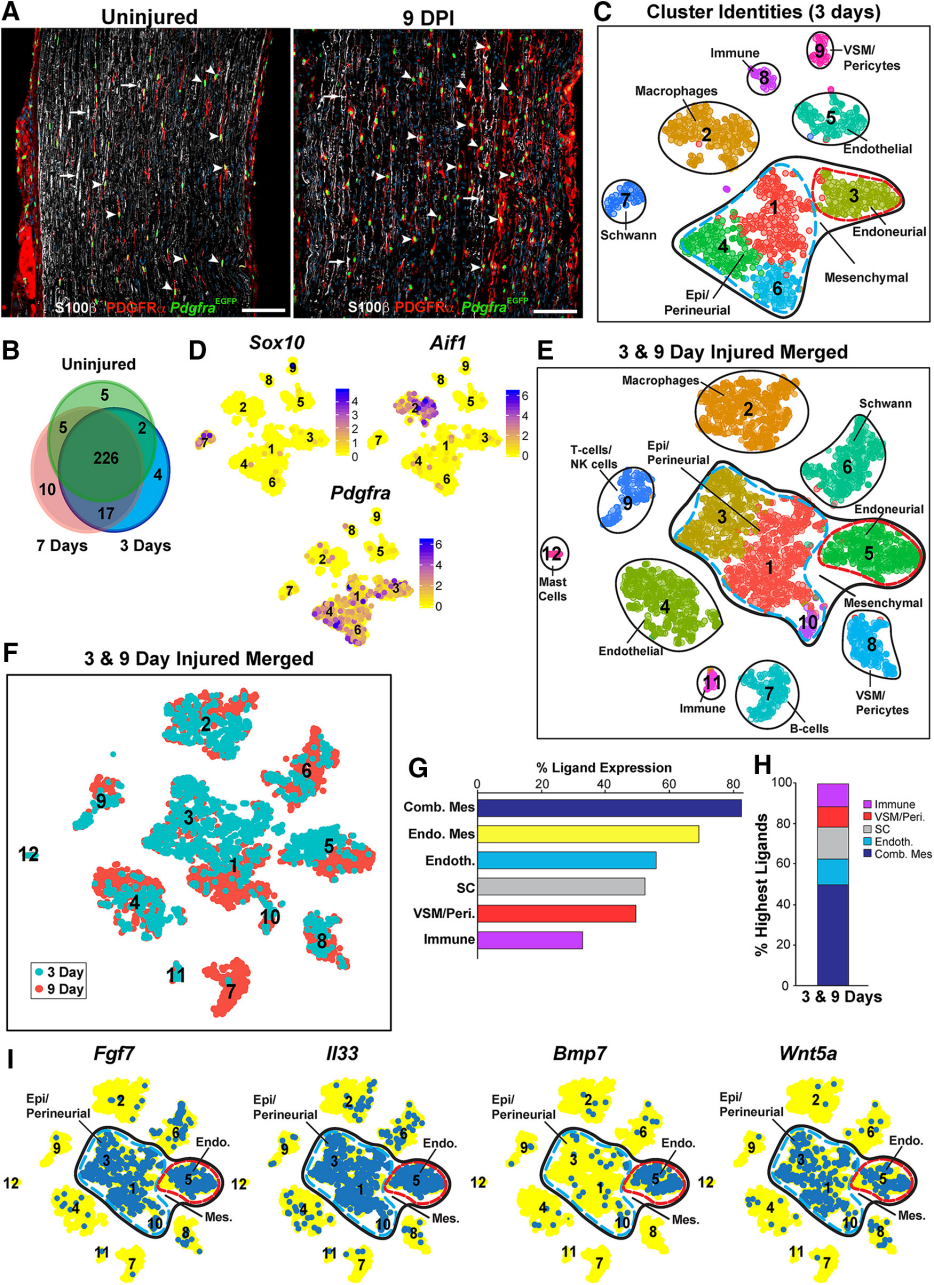
Characterization of ligand expression in the injured sciatic nerve (see also Extended Data Fig. 1-1). ***A***, Images of longitudinal sections of an uninjured adult nerve and a 9 DPI distal sciatic nerve from *Pdgfra^Egfp/+^* mice analyzed for EGFP (green) and immunoreactivity for PDGFRα (red) and S100β (white). Arrowheads denote endoneurial cells positive for both PDGFRα protein and nuclear *Pdgfra*-EGFP and arrows indicate S100β immunoreactive Schwann cells. Scale bars = 100 μm. ***B***, Venn diagram showing the number of ligands expressed in the uninjured versus 3 and 7 DPI distal sciatic nerves, based on microarray analysis. Ligand mRNAs were defined as expressed if their levels were ≥*Ntf3*. ***C–I***, Characterization of ligand expression in injured distal sciatic nerve scRNA-seq datasets. ***C***, t-SNE cluster visualization of 3 DPI sciatic nerve cell transcriptomes analyzed via the computational pipeline, with clusters annotated for cell types as identified by marker gene expression. ***D***, t-SNE gene expression overlays on the dataset in ***C*** for the Schwann cell marker *Sox10*, the macrophage marker *Aif1*, and the mesenchymal cell marker *Pdgfra*. Relative transcript expression levels are color coded as per the adjacent color keys. ***E***, t-SNE cluster visualization of the combined 3 and 9 DPI distal sciatic nerve cell transcriptomes with clusters annotated for cell types as identified by marker gene expression. ***F***, t-SNE visualization of the dataset in ***E*** with cells color coded for their dataset of origin. Numbers correspond to cluster numbers in ***E***. ***G***, Bar graph showing the percentage of the 143 injured nerve ligand mRNAs detectably expressed in the combined 3 and 9 DPI sciatic nerve cell types (shown and annotated in ***E***), including *Pdgfra*-positive mesenchymal cells (Comb. Mes), *Pdgfra*-positive endoneurial mesenchymal cells (Endo. Mes), endothelial cells (Endoth.), Schwann cells (SC), VSM/pericyte cells (VSM/Peri.), and immune cells. Ligand mRNAs were considered to be expressed if they were detected in 2% or more of the cell type of interest. ***H***, Stacked bar graph showing the relative percentage of ligand mRNAs expressed at the highest levels in the different injured peripheral nerve cell types shown in ***E***. ***I***, t-SNE gene expression overlays of the combined 3 and 9 DPI sciatic nerve dataset (shown in ***E***) for *Fgf7*, *Il33*, *Bmp7*, and *Wnt5a*. Cells that detectably express the ligand are colored blue and the numbers correspond to the clusters.

**Figure 2.**
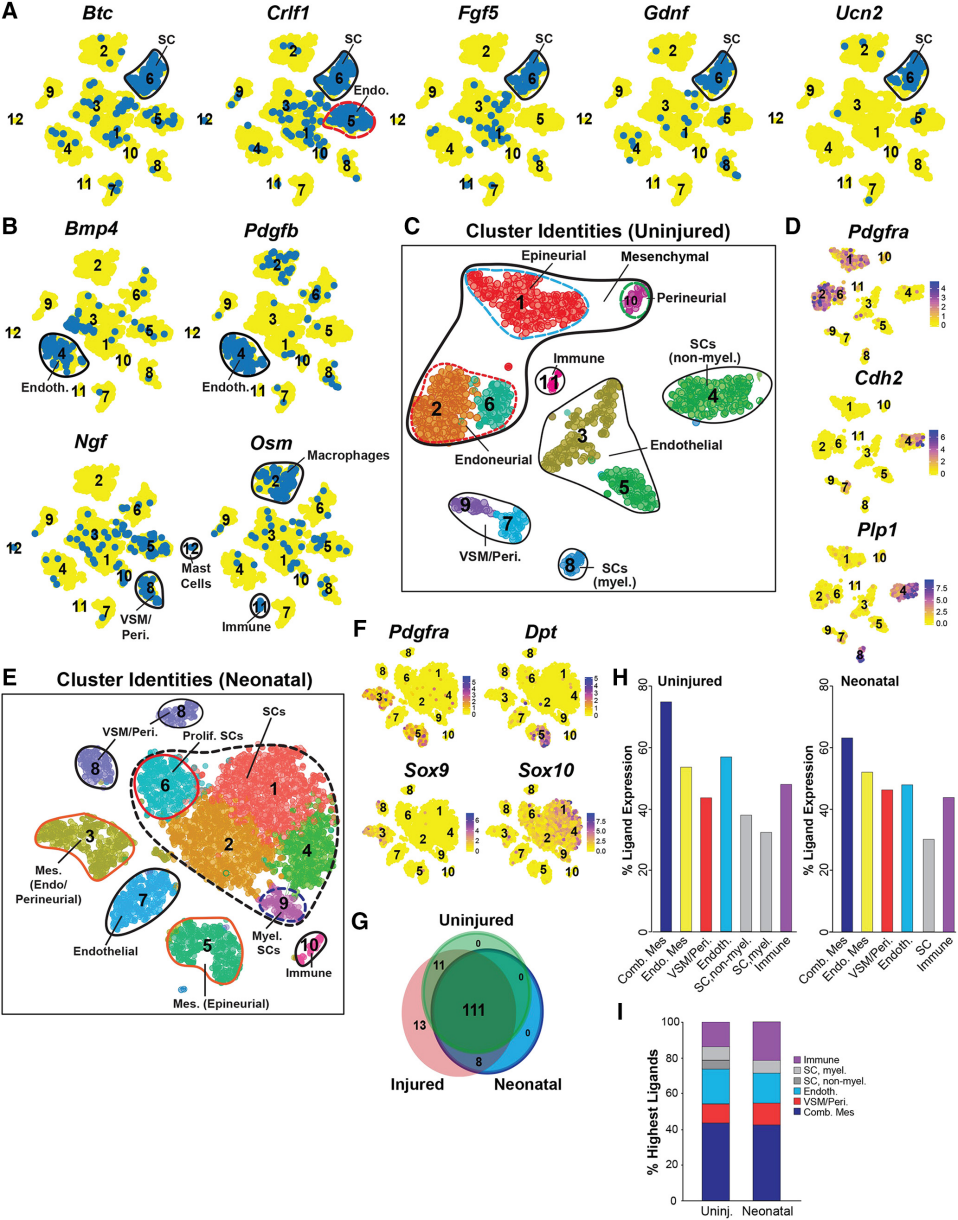
Ligand expression in the injured, uninjured and neonatal sciatic nerves (see also Extended Data Fig. 2-1). ***A***, ***B***, t-SNE gene expression overlays of the combined 3 and 9 DPI sciatic nerve dataset (shown in Fig. 1*E*) for *Btc*, *Crlf1*, *Fgf5*, *Gdnf*, and *Ucn2* (***A***) and *Bmp4*, *Pdgfb*, *Ngf*, and *Osm* (***B***). Cells that detectably express the ligand are colored blue and the numbers correspond to the clusters. Specific cell types with the highest ligand expression are circled and annotated, including Schwann cells (SC), endoneurial mesenchymal cells (Endo.), endothelial cells (Endoth.), VSM/pericytes (VSM/Peri.), and various types of immune cells. ***C***, t-SNE cluster visualization of uninjured sciatic nerve single-cell transcriptomes annotated for cell types as identified by marker gene expression. ***D***, t-SNE gene expression overlays of the dataset in ***C*** for the mesenchymal cell gene *Pdgfra*, and for the Schwann cell genes *Cdh2* and *Plp1*. Relative transcript expression levels are color coded as per the adjacent color keys. ***E***, t-SNE cluster visualization of neonatal sciatic nerve single-cell transcriptomes annotated for cell types identified by marker gene expression. Mes. = mesenchymal cells. ***F***, t-SNE gene expression overlays of the dataset in ***E*** for the mesenchymal cell genes *Pdgfra*, *Dpt*, and *Sox9,* and the Schwann cell gene *Sox 10*. Relative transcript expression levels are color coded as per the adjacent color key. ***G***, Venn diagram showing overlapping expression of the 143 injured nerve ligand mRNAs in the uninjured, neonatal, and injured nerve scRNA-seq datasets. Ligand mRNAs were considered to be expressed if they were detectable in 2% or more cells in any defined cell type cluster. ***H***, Bar graphs showing the percentage of the 143 injured nerve ligand mRNAs detectably expressed in the uninjured or neonatal sciatic nerve cell types (shown and annotated in ***C***, ***E***), including *Pdgfra*-positive mesenchymal cells (Comb. Mes), *Pdgfra*-positive endoneurial mesenchymal cells (Endo. Mes), endothelial cells (Endoth.), Schwann cells (SC; for the uninjured, also designated as myelinating vs non-myelinating), VSM/pericyte cells (VSM/Peri.), or immune cells. Ligand mRNAs were considered to be expressed if they were detected in 2% or more cells of that particular cell type. ***I***, Stacked bar graphs showing the relative percentages of ligand mRNAs expressed at the highest levels in the different uninjured and neonatal peripheral nerve cell types shown in ***C***, ***E***, respectively.

For preparation of the 3 d postinjury (DPI) nerve scRNA-seq dataset, young adult CD1 mice underwent unilateral surgical resections as described above, and injured distal sciatic nerve segments were collected 3 d following surgery. For the uninjured nerve and neonatal nerve analyses, bilateral sciatic nerve segments were collected from adult and postnatal day (P)2–P4 CD1 mice, respectively. Freshly dissected nerves were digested in a mixture of collagenase Type XI (1 mg/ml, Sigma) and 0.05% Trypsin-EDTA (Thermo Fisher Scientific) for 30 min at 37°C. Enzymatic digestion was halted by diluting the cell suspension with HBSS (Thermo Fisher Scientific). Following centrifugation (1200 rpm for 5 min) and removal of the supernatant, the cell pellet was resuspended in PBS containing 0.5% BSA and passed through a 70-μm cell strainer (BD Biosciences). For datasets purified with myelin removal beads (3 DPI, neonatal and uninjured nerve; as shown in Figs. 1*C*,*E*, 2*C*,*E*; referred to as set 2 for the neonatal analyses, where cells were prepared in two ways), myelin debris was removed from the single-cell suspension using Myelin Removal Beads II and a MidiMACS magnetic separator with LS columns (Miltenyi Biotec), according to the manufacturer’s instructions. Following myelin removal, the cell suspension was centrifuged (1200 rpm for 5 min), and the supernatant was removed before resuspending the pellet in 0.22-mm sterile-filtered PBS containing 0.01% BSA. For the second neonatal nerve dataset that was purified using fluorescence-activated cell sorting (FACS), a single-cell suspension of dissociated injured nerve cells was prepared as described above. After passing the cells through a 70-μm cell strainer and resuspending them in PBS containing 0.25% BSA, Hoechst 33258 was added to distinguish nucleated cells from myelin debris, in addition to propidium iodide (PI) to exclude dead cells. The Hoechst^high^ and PI-negative cell fractions were FACS purified using a MoFlo XDP cell sorter (Beckmann Coulter) before proceeding with scRNA-seq analysis. In all cases, cells were then resuspended in PBS containing 0.01% BSA, counted with a hemocytometer, and the solution was adjusted to a final concentration of 140,000 cells/ml and run through the Drop-seq apparatus at the Princess Margaret Genomics Facility. Drop-seq, cDNA amplification, library preparation, sequencing, processing of FASTQ sequencing reads, and read alignment steps were all conducted including minor modifications according to previously published protocols (Macosko et al., 2015). For the 3 DPI nerve scRNA-seq analysis (as shown in Fig. 1*C*), a raw digital gene expression (DGE) matrix was generated from 2500 cell barcodes as described in the Drop-seq Alignment Cookbook (version 1.2, January 2016; http://mccarrolllab.com/dropseq/). Similarly, for the uninjured nerve scRNA-seq analysis (as shown in Fig. 2*C*), a raw DGE matrix was generated from 2000 cell barcodes and used for all further analyses. In the case of the two neonatal nerve datasets (FACS sorted and bead treated), 2500 and 6200 cell barcodes were used to generate the DGE matrices as described above. DGE matrices described here were used for all subsequent analyses. The previously published DGE matrices for the 9-d injured nerve datasets (both FACS and myelin bead treated; GEO:GSE120678) were described in Carr et al. (2019).

10.1523/ENEURO.0066-20.2020.f1-1 Extended Data Figure 1-1 Characterization of the 3- and 9-d injured sciatic nerve scRNA-seq datasets. ***A***, t-SNE gene expression overlays on the 3 DPI total cell dataset (shown in Fig. 1*C* and in the adjacent legend) for the endothelial cell marker *Pecam1*, the immune cell marker *Trbc2*, the VSM/pericyte cell marker *Rgs5*, and the *Pdgfra*-positive mesenchymal epineurial and endoneurial cell markers *Dpp4* and *Wif1*. Relative transcript expression levels are color coded as per the adjacent color keys and numbers correspond to clusters. ***B***, t-SNE gene expression overlays on the combined 3 and 9 DPI total cell datasets (shown in Figure 1E and in the adjacent legend) for the endothelial cell marker *Pecam1*, the immune cell marker *Aif1*, the VSM/pericyte cell marker *Rgs5,* the mesenchymal marker *Pdgfra*, the Schwann cell marker *Sox10*, and the B cell marker *Cd19*. Relative transcript expression levels are color coded as per the adjacent color keys and numbers correspond to clusters. ***C***, t-SNE gene expression overlays on the combined 3 and 9 DPI total cell dataset (shown in Fig. 1*E* and in the adjacent legend) for markers for the different types of *Pdgfra*-positive mesenchymal cells, including *Etv1*-positive endoneurial cells, *Pcolce2*-positive epineurial cells, *Msln-*positive perineurial cells, and *Dlk1*-positive differentiating mesenchymal cells. Relative transcript expression levels are color coded as per the adjacent color keys and numbers correspond to clusters. ***D***, t-SNE gene expression overlays of the combined 3 and 9 DPI total cell dataset for *Fgf10*, *Adm*, *Pthlh*, and *Ntn1*. Cells that detectably express the ligand are colored blue and the numbers correspond to the clusters. Specific cell types are circled and annotated, including mesenchymal (Mes.), endoneurial (Endo.), and epineurial/perineurial (Epi/Perineurial) cells. Download Figure 1-1, TIF file

10.1523/ENEURO.0066-20.2020.f2-1 Extended Data Figure 2-1 Characterization of the uninjured and neonatal sciatic nerve scRNA-seq datasets. ***A***, t-SNE gene expression overlays of the combined 3 and 9 DPI total cell dataset (shown in Fig. 1*E* and the adjacent legend) for *Bdnf*, *Dhh*, and *Shh*. Cells that detectably express the ligand are colored blue and the numbers correspond to the clusters. Schwann cells are circled and annotated (SC). ***B***, t-SNE gene expression overlays on the uninjured sciatic nerve total cell dataset (shown in Fig. 2*C* and in the adjacent legend) for the VSM/pericyte cell marker *Acta2,* the endothelial cell marker *Pecam1*, the immune cell marker *Cd52*, and the proliferating cell marker *Top2a*. Relative transcript expression levels are color coded as per the adjacent color keys and numbers correspond to clusters. ***C***, t-SNE cluster visualization of neonatal sciatic nerve single-cell transcriptomes (as in Fig. 2*E* and the adjacent legend) showing dataset of origin. Set 1 (red) refers to the neonatal nerve cells isolated by FACS and Set 2 (blue) to the neonatal nerve cells isolated by treatment with the myelin removal beads. The right t-SNE cluster visualization indicates the datasets of origin following Harmony data integration batch correction of the combined datasets. ***D***, t-SNE gene expression overlays on the neonatal sciatic nerve total cell dataset (shown in Fig. 2*E* and in the adjacent legend) for *Osr2*, which marks endoneurial mesenchymal cells, *Casq2*, which is expressed in perineurial cells, the endothelial cell marker *Pecam1*, the VSM/pericyte cell marker *Acta2*, and the proliferating cell marker *Top2a*. Relative transcript expression levels are color coded as per the adjacent color keys and numbers correspond to clusters. Download Figure 2-1, TIF file.

### Computational analysis of scRNA-seq data

Drop-seq data (DGE matrices) were analyzed used a previously described custom computational pipeline (Yuzwa et al., 2017; Carr et al., 2019; Storer et al., 2020; described in detail in Innes and Bader, 2019). Briefly, data were filtered to remove cells with low unique molecular identifier counts, cell doublets, contaminant red blood cells, and cells that contained high mitochondrial gene content. Genes detected in less than three cells were removed. Cell transcriptomes were then normalized as previously described (Lun et al., 2016) using an algorithm in the scran package in R that corrects for differences in sequencing depth by the use of scaling factors within each cell by pooling random subsets of cells, summing their library sizes, and comparing them with average library size across all cells in the group. This is iteratively performed, and the cell-wise scaling factors can be deconvolved from the set of pool-wise scaling factors. Following normalization, DGE matrices were imported into Seurat (v. 1.4.0.16) in R. Principal component (PC) analysis was then undertaken using highly variable genes, and clustering analysis was performed using top PCs. This analysis was conducted using the shared nearest neighbor (SNN) modularity optimization-based clustering algorithm implemented in Seurat (FindClusters function). Clustering was iteratively performed at increasing resolution until a lower limit of ∼10–20 differentially expressed genes [calculated by the Seurat FindMarkers function, *p* < 0.01 family-wise error rate (FWER), Holm’s method] was reached between the most similar clusters. For conservative analysis of all datasets analyzed, clusters were assigned at the lowest resolution that still distinguished distinct cell types, as defined by established marker genes. As a result, clusters were assigned at a resolution of 0.4 for analysis of all datasets.

For the 3 DPI dataset (2075 total cells), nine clusters were identified with 210 differentially expressed genes between most similar clusters (*p* < 0.01, FWER). Cells were sequenced to an average depth of >70,000 reads/cell. The average number of genes detected per cell was 1027 ± 588, and the average number of transcripts was 2257 ± 2855. For the 3 and 9 DPI nerve combined dataset, cell transcriptomes from the 3 DPI dataset and the myelin bead removal-treated 9 DPI dataset (from Carr et al., 2019; GEO:GSE120678) were merged using the unique cell identifier barcodes from all cells present in all clusters of the two datasets following pipeline processing. The constructed raw DGE matrices of the combined datasets were then re-run through the pipeline for re-clustering, resulting in 5395 total cells. Twelve clusters were identified with 200 differentially expressed genes between most similar clusters (*p* < 0.01, FWER). The uninjured nerve dataset was previously analyzed for *Pdgfra*-positive mesenchymal cells (Carr et al., 2019), but not for other cell types. Reanalysis of this dataset (1841 total cells) identified 11 clusters with 105 differentially expressed genes between the most similar clusters (*p* < 0.01, FWER). For the combined neonatal dataset, cell transcriptomes from both the FAC-sorted (set 1; Extended Data Fig. 2-1*C*) and myelin removal bead-treated (set 2; Extended Data Fig. 2-1*C*) samples were merged using the unique cell identifier barcodes from all cells present in all clusters of the two datasets following pipeline processing. The constructed raw DGE matrices of the combined datasets were then re-run through the pipeline for re-clustering, resulting in 6885 total cells. Ten clusters were identified with 540 differentially expressed genes between most similar clusters (*p* < 0.01, FWER). For the FAC-sorted sample, cells were sequenced to an average depth of >90,000 reads/cell. The average number of genes detected per cell was 1005 ± 728 and the average number of transcripts was 2044 ± 1985. For the bead treated sample, cells were sequenced to an average depth of >43,000 reads/cell. The average number of genes detected per cell was 732 ± 485, and the average number of transcripts was 1231 ± 1095.

**Figure 3.**
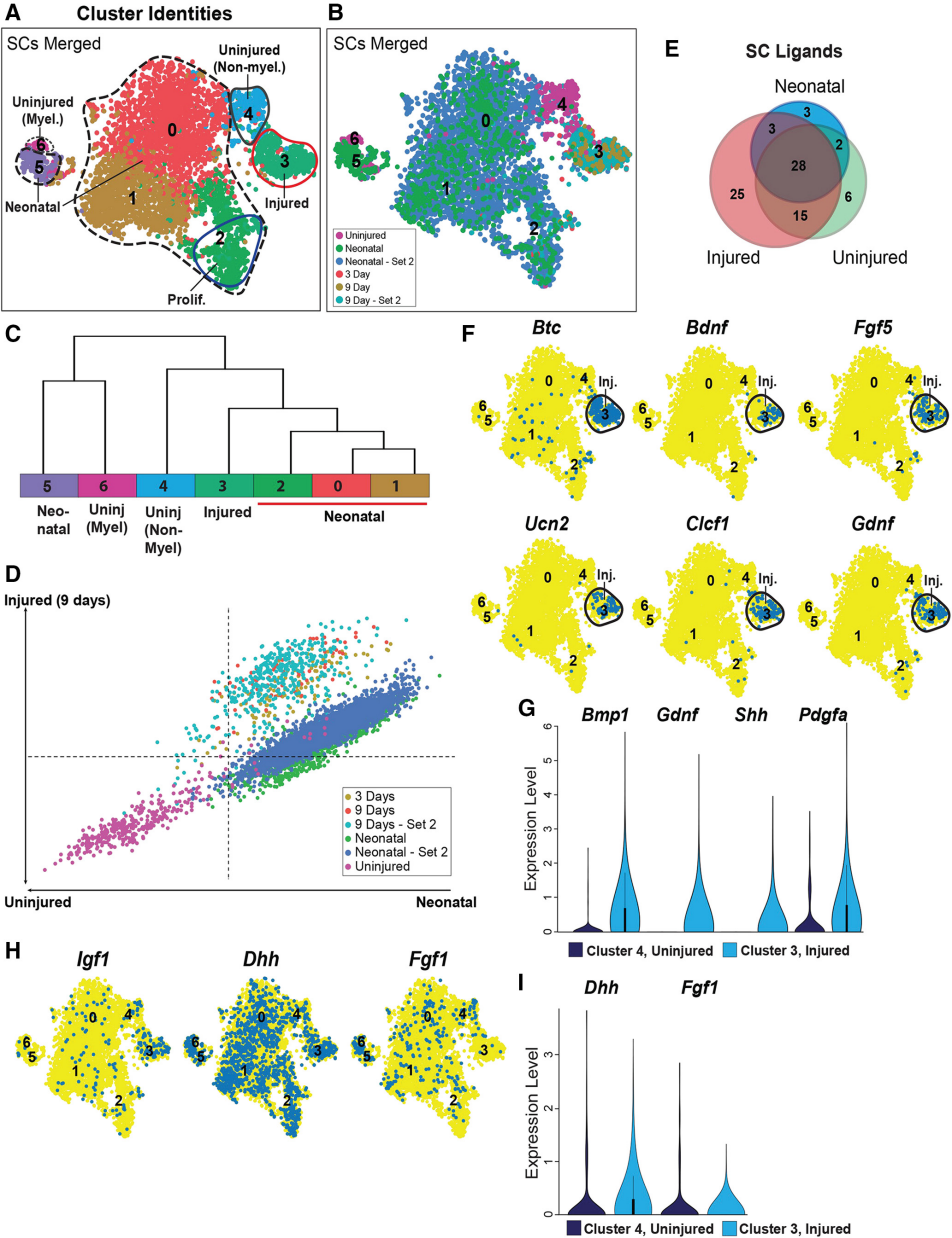
Characterization of ligand expression in sciatic nerve Schwann cells (see also Extended Data Fig. 3-1). Schwann cell transcriptomes from the injured, uninjured and neonatal nerve datasets (in Figs. 1*E*, 2*C*,*E*; also see Carr et al., 2019) were extracted, combined together, analyzed and batch-corrected using Harmony data integration and clustered in Seurat based on principal components. ***A***, ***B***, t-SNE visualizations of the combined Schwann cells (SCs), showing clusters (***A***) and datasets of origin (***B***). The clusters in ***A*** are also annotated based on marker gene expression (Extended Data Fig. 3-1*A*) and datasets of origin. Neonatal and 9 d refer to the FAC-sorted preparations at these timepoints while neonatal – set 2 and 9 d – set 2 refer to the cells prepared with myelin removal beads. ***C***, A dendrogram showing hierarchical analysis of cell clusters from the combined Schwann cell dataset, with cluster identity numbers, annotations and colors as in ***A***. ***D***, Scatterplot showing differential correlation of single-cell transcriptomes from the combined Schwann cell dataset in ***A*** (individual colors represent different datasets) with bulk transcriptomes from uninjured adult versus neonatal Schwann cells on the *x*-axis and uninjured adult versus 9 DPI on the *y*-axis. ***E***, Venn diagram showing the overlap of ligand mRNAs expressed in neonatal, injured and uninjured Schwann cell clusters from the combined Schwann cell dataset in ***A***. Ligand mRNAs were considered to be expressed if they were detectable in 2% or more cells in any defined cell type cluster. ***F***, t-SNE gene expression overlays of the combined Schwann cell data in ***A*** for *Btc*, *Bdnf*, *Fgf5*, *Ucn2*, *Clcf1*, and *Gdnf*. Cells that detectably express the ligand are colored blue, and the numbers correspond to the clusters. Injured Schwann cell cluster 3 (Inj.) is circled. ***G***, Violin plots showing the relative expression of *Bmp1*, *Gdnf*, *Shh*, and *Pdgfa* in injured Schwann cluster 3 versus uninjured Schwann cell cluster 4 from the dataset in ***A***. ***H***, t-SNE gene expression overlays of the combined Schwann cell data in ***A*** for *Igf1*, *Dhh*, and *Fgf1*. Cells that detectably express the ligand are colored blue and the numbers correspond to the clusters. ***I***, Violin plots showing the relative expression of *Dhh* and *Fgf1* in injured Schwann cell cluster 3 versus uninjured cluster 4 from the dataset in ***A***.

**Figure 4.**
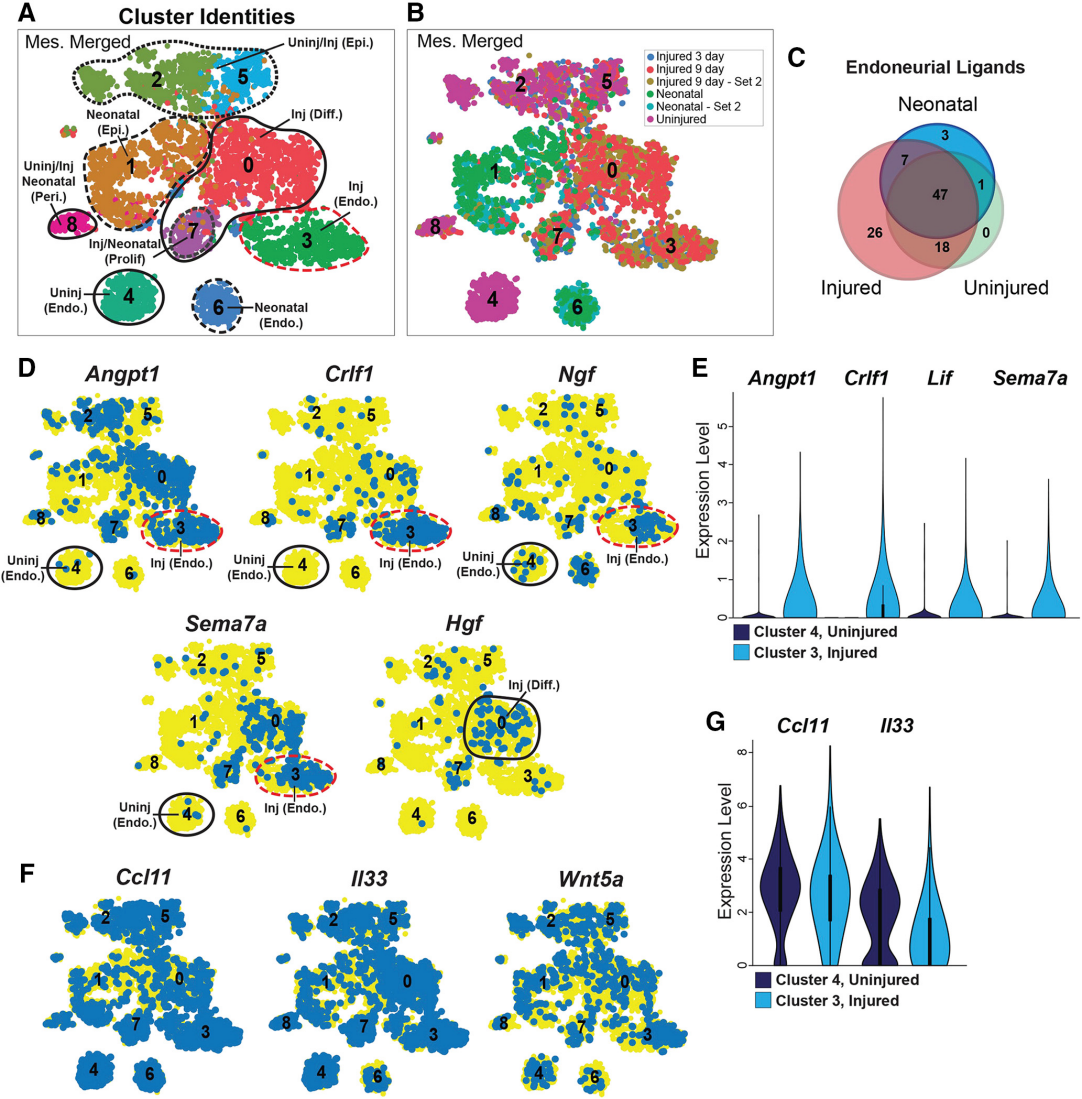
Characterization of ligand expression in sciatic nerve mesenchymal cells (see also Extended Data Fig. 4-1). *Pdgfra*-positive mesenchymal cell transcriptomes from the injured, uninjured and neonatal nerve datasets (in Figs. 1*E*, 2*C*,*E*; also see Carr et al., 2019) were extracted, combined together, analyzed and batch-corrected using Harmony data integration and clustered in Seurat based on principal components. ***A***, ***B***, t-SNE visualizations of the combined mesenchymal cells showing clusters (***A***) and datasets of origin (***B***). The clusters in ***A*** are also annotated based on marker gene expression (Extended Data Fig. 4-1) and datasets of origin. Neonatal and 9 d refer to the FAC-sorted preparations at these timepoints, while neonatal – set 2 and 9 d – set 2 refer to the cells prepared with myelin removal beads. ***C***, Venn diagram showing the overlap of ligand mRNAs expressed in neonatal (cluster 6), injured (cluster 3), and uninjured (cluster 4) nerve endoneurial mesenchymal cells from the combined mesenchymal nerve dataset in ***A***. Ligand mRNAs were considered to be expressed if they were detectable in 2% or more cells in the relevant cluster. ***D***, t-SNE gene expression overlays of the combined mesenchymal cell data in ***A*** for *Angpt1*, *Crlf1*, *Ngf*, *Sema7a*, and *Hgf.* Cells that detectably express the ligand are colored blue, and the numbers correspond to the clusters. Relevant clusters are circled and annotated, including uninjured endoneurial (Uninj Endo.), injured endoneurial (Inj Endo.), and injured differentiating (Inj Diff.) mesenchymal cells. ***E***, Violin plots showing the relative expression of *Angpt1*, *Crlf1*, *Lif*, and *Sema7a* in injured endoneurial mesenchymal cell cluster 3 versus uninjured endoneurial cell cluster 4 from the dataset in ***A***. ***F***, t-SNE gene expression overlays of the combined mesenchymal cell data in ***A*** for *Ccl11*, *Il33*, and *Wnt5a*. Cells that detectably express the ligand are colored blue, and the numbers correspond to the clusters. ***G***, Violin plots showing the relative expression of *Ccl11* and *Il33* in injured endoneurial mesenchymal cell cluster 3 versus uninjured endoneurial cell cluster 4 from the dataset in ***A***.

For the combined Schwann cell dataset (Fig. 3*A*,*B*), the unique cell identifier barcodes from all cells present in *Sox10*-positive clusters in each of the six described datasets (Figs. 1*E*, 2*C*,*E*; FAC-sorted cells in Carr et al., 2019) were merged. For the combined mesenchymal cell dataset (Fig. 4*A*,*B*), the unique cell identifier barcodes from all cells present in *Pdgfra*-positive clusters in each of the same six datasets were merged. The constructed raw DGE matrices of the combined datasets were then re-run through the pipeline, resulting in 5331 total Schwann cells and 5416 total mesenchymal cells. Batch correction of these combined datasets as well as data shown in Extended Data Figure 2-1*C* was performed using the Harmony batch-effect-correction method (Korsunsky et al., 2019) with Seurat V2. Briefly, gene expression data from the combined datasets were transferred to Seurat, where highly variable genes were then used to carry out principal component analysis. The Harmony iterative algorithm was used to integrate datasets and adjust for dataset specific effects based on the top 20 principal components. Iterative clustering was performed using the FindClusters function in Seurat V2, with clusters being assigned at a resolution of 0.4. This resulted in seven and nine clusters in the combined Schwann cell and mesenchymal cell datasets, respectively. t-Distributed stochastic neighbor embedding (t-SNE) visualizations of batch-corrected data were generated using the FeaturePlot function in Seurat. t-SNE gene expression overlays displayed in the figures were generated using the FeaturePlot function in Seurat and binary expression overlays were performed using the SubsetCells and TSNEPlot functions in Seurat. These tSNE overlays were further edited using Adobe Illustrator (Adobe Systems Incorporated) as necessary to highlight features of the t-SNE visualization.

10.1523/ENEURO.0066-20.2020.f3-1 Extended Data Figure 3-1 Characterization of the combined Schwann cell sciatic nerve scRNA-seq dataset. ***A***, t-SNE gene expression overlays on the combined and batch-corrected neonatal, injured adult and uninjured adult Schwann cell data (shown in Fig. 3*A* and the adjacent legend) for *Sox10*, the myelination gene *Mag*, the pre-myelinating Schwann cell marker *Pou3f1*, the non-myelinating Schwann cell gene *Emp1*, and the proliferation marker *Top2a*. Relative transcript expression levels are color coded as per the adjacent color keys and numbers correspond to clusters. ***B***, Plots show correlation analyses of average transcript expression in the in the combined injured nerve dataset (Fig. 1*E*) showing Schwann cell cluster 6 compared to endoneurial cell cluster 5 (left plot) and to epineurial cell cluster 3 (right plot). Outlier transcripts expressed in the mesenchymal cell clusters are highlighted red and labelled. ***C***, ***D***, t-SNE gene expression overlays of the combined Schwann cell data (shown in Fig. 3*A* and the adjacent legend) for *Ccl3*, *Crlf1*, *Lif*, *Shh*, and *Tgfb1* (***C***) and *Bmp1*, *Fgf7*, *Mdk*, and *Pdgfa* (***D***). Cells that detectably express the ligand are colored blue and the numbers correspond to the clusters. Injured Schwann cell cluster 3 is circled and annotated (***C***, Inj.). Download Figure 3-1, TIF file.

10.1523/ENEURO.0066-20.2020.f4-1 Extended Data Figure 4-1 Characterization of the combined *Pdgfra*-positive mesenchymal cell sciatic nerve scRNA-seq dataset. t-SNE gene expression overlays on the combined and batch-corrected neonatal, injured adult and uninjured adult *Pdgfra*-positive mesenchymal cell data (shown in Fig. 4*A* and the adjacent legend) for *Pdgfra*, the epineurial markers *Pcolce2* and *Dpp4*, the perineurial gene *Slc2a1*, the endoneurial gene *Meox1*, the differentiating injured cell genes *Mest* and *Dlk1*, and the proliferation gene *Top2a*. Relative transcript expression levels are color coded as per the adjacent color keys and numbers correspond to clusters. Download Figure 4-1, TIF file.

Cell types (clusters) were defined based on the expression of the following established marker genes: endothelial cells: *Pecam1*/*Cd31*, *Plvap*, and *Esam*; Schwann lineage cells: *Ngfr*/*p75NTR* and *Sox10*; non-myelinating Schwann cells: *Ngfr/p75NTR*, *Cdh2*, *L1cam*, *Ednrb*, *Emp1*, and *Sema3e*; premyelinating Schwann cells: *Pou3f1* and *Egr2*; myelinating Schwann cells: *Mag*, *Mbp*, *Pmp22*, *Mpz*, and *Plp1*; macrophages/monocytes: *Aif1/Iba1*; lymphoid immune cells including B cells, T cells, and NK cells: *Ptprcap*, *Trbc2*, and *Cd52*; B cells: *Cd19*; vasculature-associated smooth muscle (VSM) and pericyte cells: *Desmin*, *Mylk*, *Acta2*, and *Rgs5*; mesenchymal cells: *Pdgfra*; epineurial mesenchymal cells: *Pcolce2*, *Dpp4*, *Dpt*, *Ly6c1*, and *Comp*; endoneurial mesenchymal cells: *Etv1*, *Wif1*, *Sox9*, *Osr2*, and *Meox1*; perineurial mesenchymal cells: *Slc2a1*, *Casq2*, and *Msln*; differentiating nerve bridge mesenchymal cells: *Dlk1* and *Mest*; and proliferating cells: *Mki67* and *Top2a*.

In combined datasets, dataset identities were distinguished by using the gg color hue and hcl functions in R. Correlation analysis comparing gene expression between different clusters was performed by averaging the expression of each gene across all cells in the individual clusters to be compared, then Pearson correlation analysis was performed using the Cor function and plotted in R. Genes of interest were then highlighted using Adobe Illustrator (Adobe Systems Incorporated; as in Extended Data Fig. 3-1*B*). Differential correlation of single-cell transcriptomes as shown in Figure 3*D* was performed as described previously (Gerber et al., 2018). Briefly, mock bulk transcriptomes were generated for the 9 DPI Schwann cells (both bead and FAC sorted), the uninjured non-myelinating Schwann cells and the neonatal Schwann cells (both bead and FAC sorted) by determining the mean expression of each gene in the total combined cells in each dataset. Each single-cell transcriptome was then correlated with each of the mock bulk transcriptomes. We then determined the differential correlation of each single cell with the bulk uninjured nerve versus the bulk 9 DPI transcriptomes (*y*-axis) and the differential correlation with the bulk uninjured nerve versus the bulk neonatal nerve transcriptomes (*x*-axis). Violin plots were generated using the VioPlot package in R. Hierarchical clustering of the batch corrected combined Schwann cell data in Figure 3*C* was performed based on the top 20 Principal Component (PC) using the BuildClusterTree and PlotClusterTree functions in Seurat. Node numbers were removed from the plot and cluster descriptions and colors were added for clarity using Adobe Illustrator (Adobe Systems Incorporated). The single cell heatmaps were generated (with scaled expression values) using the DoHeatMap function in Seurat at resolution 0.4.

### Ligand mRNA expression and Venn diagram analysis

The expression of ligand mRNAs (Table 1) was characterized from the whole nerve microarray analysis using a custom curated ligand-receptor database (modified from Yuzwa et al., 2016). Extracellular matrix proteins and potential ligands without well-defined, receptor-mediated paracrine roles were excluded. The VennDiagram package in R was used to determine overlapping ligands in the uninjured, 3-d injured, and 7-d injured nerve datasets and was modified to show proportional representation of data using the euler*APE* tool (Micallef and Rodgers, 2014).

**Table 1:** Ligand mRNAs expressed in uninjured, 3 DPI, and 7 DPI sciatic nerves using global transcriptomic analysis

Uninjured (238)		3 DPI (249)		7 DPI (258)		Uninjured,3 DPI, and 7 DPIintersect (226)
Adipoq		Adipoq		Adipoq		Adipoq
Adm		Adm		Adm		Adm
Agt		Agt		Agt		Agt
Angpt1		Angpt1		Angpt1		Angpt1
Angpt2		Angpt2		Angpt2		Angpt2
Angpt4		Angpt4		Angpt4		Angpt4
Apln		Apln		Apln		Apln
Artn		Areg^++^		Areg^++^		Artn
Avp		Artn		Artn		Avp
Bdnf		Avp		Avp		Bdnf
Bmp1		Bdnf		Bdnf		Bmp1
Bmp2		Bmp1		Bmp1		Bmp2
Bmp4		Bmp2		Bmp2		Bmp4
Bmp5		Bmp4		Bmp4		Bmp5
Bmp6		Bmp5		Bmp5		Bmp6
Bmp7		Bmp6		Bmp6		Bmp7
Btc		Bmp7		Bmp7		Btc
Cck		Btc		Btc		Cck
Ccl11		Cck		Btla^+^		Ccl11
Ccl19		Ccl11		Calca^+^		Ccl19
Ccl2		Ccl17^*^^*^		Cck		Ccl2
Ccl21		Ccl19		Ccl11		Ccl21
Ccl22		Ccl2		Ccl19		Ccl22
Ccl24		Ccl20^++^		Ccl2		Ccl24
Ccl25		Ccl21		Ccl20^++^		Ccl25
Ccl27		Ccl22		Ccl21		Ccl27
Ccl3		Ccl24		Ccl22		Ccl3
Ccl5		Ccl25		Ccl24		Ccl5
Ccl7		Ccl27		Ccl25		Ccl7
Clcf1		Ccl3		Ccl27		Clcf1
Clu		Ccl5		Ccl3		Clu
Cmtm8		Ccl7		Ccl5		Cmtm8
Cntf		Cga^++^		Ccl7		Cntf
Cntn1		Clcf1		Cga^++^		Cntn1
Cntn2		Clu		Clcf1		Cntn2
Copa		Cmtm8		Clu		Copa
Crlf1		Cntf		Cmtm8		Crlf1
Csf1		Cntn1		Cntf		Csf1
Cst3		Cntn2		Cntn1		Cst3
Ctf1		Copa		Cntn2		Ctf1
Ctgf		Crlf1		Copa		Ctgf
Cx3cl1		Csf1		Crlf1		Cx3cl1
Cxcl1		Cst3		Csf1		Cxcl1
Cxcl10		Ctf1		Cst3		Cxcl10
Cxcl12		Ctgf		Ctf1		Cxcl12
Cxcl13		Cx3cl1		Ctgf		Cxcl13
Cxcl16		Cxcl1		Cx3cl1		Cxcl16
Cxcl2		Cxcl10		Cxcl1		Cxcl2
Cxcl9		Cxcl12		Cxcl10		Cxcl9
Dhh		Cxcl13		Cxcl11^+^		Dhh
Dll1		Cxcl16		Cxcl12		Dll1
Dll4		Cxcl2		Cxcl13		Dll4
Ebi3		Cxcl9		Cxcl16		Ebi3
Eda		Dhh		Cxcl2		Eda
Edn3		Dll1		Cxcl9		Edn3
Efna1		Dll4		Dhh		Efna1
Efna2		Ebi3		Dll1		Efna2
Efna4		Eda		Dll4		Efna4
Efna5		Edn3		Ebi3		Efna5
Efnb1		Efna1		Eda		Efnb1
Efnb2		Efna2		Edn3		Efnb2
Efnb3		Efna4		Efna1		Efnb3
Epo		Efna5		Efna2		Epo
Esm1		Efnb1		Efna4		Esm1
Fgf1		Efnb2		Efna5		Fgf1
Fgf10		Efnb3		Efnb1		Fgf17
Fgf17		Epo		Efnb2		Fgf18
Fgf18		Esm1		Efnb3		Fgf19
Fgf19		Fgf1		Epo		Fgf2
Fgf2		Fgf17		Esm1		Fgf4
Fgf4		Fgf18		Fgf1		Fgf5
Fgf5		Fgf19		Fgf10		Fgf7
Fgf7		Fgf2		Fgf17		Figf
Figf		Fgf4		Fgf18		Fjx1
Fjx1		Fgf5		Fgf19		Flt3lg
Flt3lg		Fgf7		Fgf2		Fstl1
Fstl1		Figf		Fgf23^+^		Gal
Gal		Fjx1		Fgf4		Gap43
Gap43		Flt3lg		Fgf5		Gas6
Gas6		Fstl1		Fgf7		Gdf10
Gdf10		Gal		Figf		Gdf11
Gdf11		Gap43		Fjx1		Gdf9
Gdf9		Gas6		Flt3lg		Gdnf
Gdnf		Gdf10		Fstl1		Ghrh
Ghrh		Gdf11		Gal		Gip
Gip		Gdf5^++^		Gap43		Gmfb
Gmfb		Gdf6^*^^*^		Gas6		Gmfg
Gmfg		Gdf9		Gdf10		Gnrh1
Gnrh1		Gdnf		Gdf11		Gpi
Gpi		Gh1^*^^*^		Gdf5^++^		Grp
Grp		Ghrh		Gdf9		Habp2
Guca2a		Gip		Gdnf		Hbegf
Habp2		Gmfb		Ghrh		Hcrt
Hbegf		Gmfg		Gip		Hdgf
Hcrt		Gnrh1		Gmfb		Hdgfrp3
Hdgf		Gpi		Gmfg		Hgf
Hdgfrp3		Grp		Gnrh1		Hmgb1
Hgf		Habp2		Gpi		Ifna4
Hmgb1		Hbegf		Grp		Igf1
Ifna1		Hcrt		Guca2a		Igf2
Ifna4		Hdgf		Habp2		Igfbpl1
Igf1		Hdgfrp3		Hbegf		Ihh
Igf2		Hgf		Hcrt		Il13
Igfbpl1		Hmgb1		Hdgf		Il15
Ihh		Ifna4		Hdgfrp3		Il16
Il13		Igf1		Hgf		Il17b
Il15		Igf2		Hmgb1		Il18
Il16		Igfbpl1		Ifna1		Il19
Il17b		Ihh		Ifna4		Il23a
Il18		Il12a^++^		Igf1		Il25
Il19		Il13		Igf2		Il33
Il21*		Il15		Igfbpl1		Inha
Il23a		Il16		Ihh		Inhba
Il25		Il17b		Il12a^++^		Inhbb
Il33		Il18		Il13		Insl3
Inha		Il19		Il15		Jag1
Inhba		Il23a		Il16		Jag2
Inhbb		Il25		Il17b		Kiss1
Insl3		Il27^*^^*^		Il18		Kitlg
Jag1		Il33		Il19		Lgals3
Jag2		Il6^++^		Il1b^+^		Lif
Kiss1		Inha		Il23a		Lrsam1
Kitlg		Inhba		Il25		Ltb
Lgals3		Inhbb		Il33		Mdk
Lgi1		Inhbe^++^		Il6^++^		Metrn
Lif		Insl3		Inha		Mif
Lrrc4		Jag1		Inhba		Mln
Lrsam1		Jag2		Inhbb		Nampt
Ltb		Kiss1		Inhbe^++^		Nenf
Mdk		Kitlg		Insl3		Ngf
Metrn		Lgals3		Jag1		Nodal
Mif		Lgi1		Jag2		Nov
Mln		Lif		Kiss1		Npb
Mst1^*^		Lrsam1		Kitlg		Npff
Nampt		Ltb		Lgals3		Nppb
Nenf		Mdk		Lif		Nppc
Ngf		Metrn		Lrrc4		Nrtn
Nodal		Mif		Lrsam1		Ntf3
Nov		Mln		Ltb		Ntf4
Npb		Mmp12^++^		Mdk		Ntn1
Npff		Mmp9^++^		Metrn		Osm
Nppb		Nampt		Mif		Oxt
Nppc		Nell2^++^		Mln		Pcsk1n
Nrtn		Nenf		Mmp12^++^		Pdap1
Ntf3		Ngf		Mmp9^++^		Pdgfa
Ntf4		Nodal		Nampt		Pdgfb
Ntn1		Nov		Nell2^++^		Pdgfc
Osm		Npb		Nenf		Pf4
Oxt		Npff		Ngf		Pgf
Pcsk1n		Nppb		Nodal		Plg
Pdap1		Nppc		Nov		Pnoc
Pdgfa		Nrtn		Npb		Prdx2
Pdgfb		Ntf3		Npff		Prdx6
Pdgfc		Ntf4		Nppb		Prlh
Pf4		Ntn1		Nppc		Proc
Pgf		Osm		Nrg1^+^		Prok1
Plg		Oxt		Nrtn		Prok2
Pnoc		Pcsk1n		Ntf3		Psip1
Prdx2		Pdap1		Ntf4		Pspn
Prdx6		Pdgfa		Ntn1		Pth2
Prlh		Pdgfb		Osm		Pthlh
Proc		Pdgfc		Oxt		Ptn
Prok1		Pf4		Pcsk1n		Rabep1
Prok2		Pgf		Pdap1		Rln3
Psip1		Plg		Pdgfa		Rspo1
Pspn		Pnoc		Pdgfb		Rtn1
Pth2		Ppy^++^		Pdgfc		Rtn4
Pthlh		Prdx2		Pf4		S100b
Ptn		Prdx6		Pgf		Scgb3a1
Rabep1		Prlh		Plg		Scrn1
Rln3		Proc		Pnoc		Sct
Rspo1		Prok1		Pomc^+^		Sema3a
Rspo3		Prok2		Ppbp^+^		Sema3b
Rspo4*		Psip1		Ppy^++^		Sema3c
Rtn1		Pspn		Prdx2		Sema3d
Rtn4		Pth2		Prdx6		Sema3e
S100b		Pthlh		Prlh		Sema3f
Scg3*		Ptn		Proc		Sema3g
Scgb3a1		Rabep1		Prok1		Sema4a
Scrn1		Rln3		Prok2		Sema4b
Sct		Rspo1		Psip1		Sema4c
Sema3a		Rtn1		Pspn		Sema4d
Sema3b		Rtn4		Pth2		Sema4g
Sema3c		S100b		Pthlh		Sema5a
Sema3d		Scgb3a1		Ptn		Sema5b
Sema3e		Scrn1		Rabep1		Sema6a
Sema3f		Sct		Rln3		Sema6b
Sema3g		Sema3a		Rspo1		Sema6c
Sema4a		Sema3b		Rspo3		Sema6d
Sema4b		Sema3c		Rtn1		Sema7a
Sema4c		Sema3d		Rtn4		Serpinh1
Sema4d		Sema3e		S100b		Sfrp1
Sema4g		Sema3f		Scgb3a1		Sfrp2
Sema5a		Sema3g		Scrn1		Sfrp4
Sema5b		Sema4a		Sct		Sfrp5
Sema6a		Sema4b		Sema3a		Shh
Sema6b		Sema4c		Sema3b		Smoc1
Sema6c		Sema4d		Sema3c		Sost
Sema6d		Sema4f^++^		Sema3d		Sparc
Sema7a		Sema4g		Sema3e		Sparcl1
Serpinh1		Sema5a		Sema3f		Spp1
Sez6		Sema5b		Sema3g		Sst
Sfrp1		Sema6a		Sema4a		Tgfa
Sfrp2		Sema6b		Sema4b		Tgfb1
Sfrp4		Sema6c		Sema4c		Tgfb2
Sfrp5		Sema6d		Sema4d		Tgfb3
Shh		Sema7a		Sema4f^++^		Thpo
Smoc1		Serpinh1		Sema4g		Timp2
Sost		Sez6		Sema5a		Tnf
Sparc		Sfrp1		Sema5b		Tnfrsf11b
Sparcl1		Sfrp2		Sema6a		Tnfsf10
Spp1		Sfrp4		Sema6b		Tnfsf11
Sst		Sfrp5		Sema6c		Tnfsf12
Tgfa		Shh		Sema6d		Tnfsf13
Tgfb1		Smoc1		Sema7a		Tnfsf14
Tgfb2		Sost		Serpinh1		Tnfsf15
Tgfb3		Sparc		Sfrp1		Tymp
Thpo		Sparcl1		Sfrp2		Ucn2
Timp2		Spp1		Sfrp4		Ucn3
Tnf		Sst		Sfrp5		Vegfa
Tnfrsf11a*		Tgfa		Shh		Vegfb
Tnfrsf11b		Tgfb1		Smoc1		Vegfc
Tnfsf10		Tgfb2		Sost		Wnt2
Tnfsf11		Tgfb3		Sparc		Wnt5a
Tnfsf12		Thpo		Sparcl1		Wnt11
Tnfsf13		Timp2		Spp1		Xcl1
Tnfsf14		Tnf		Sst		
Tnfsf15		Tnfrsf11b		Tdgf1^+^		
Tymp		Tnfsf10		Tgfa		
Ucn2		Tnfsf11		Tgfb1		
Ucn3		Tnfsf12		Tgfb2		
Vegfa		Tnfsf13		Tgfb3		
Vegfb		Tnfsf13b^++^		Thpo		
Vegfc		Tnfsf14		Timp2		
Wnt2		Tnfsf15		Tnf		
Wnt5a		Tnfsf8^++^		Tnfrsf11b		
Wnt11		Tnfsf9^++^		Tnfsf10		
Xcl1		Tslp^++^		Tnfsf11		
		Tymp		Tnfsf12		
		Ucn2		Tnfsf13		
		Ucn3		Tnfsf13b^++^		
		Vegfa		Tnfsf14		
		Vegfb		Tnfsf15		
		Vegfc		Tnfsf8^++^		
		Wnt2		Tnfsf9^++^		
		Wnt5a		Tslp^++^		
		Wnt7a^++^		Tymp		
		Wnt11		Ucn2		
		Xcl1		Ucn3		
				Vegfa		
				Vegfb		
				Vegfc		
				Wnt1^+^		
				Wnt2		
				Wnt5a		
				Wnt7a^++^		
				Wnt11		
				Xcl1		

Ligands identified in 3 DPI, 7 DPI, and uninjured rat sciatic nerves based on microarray analysis and our curated ligand-receptor database (modified from Yuzwa et al., 2016). Ligands were considered expressed if their expression levels exceeded that of *Ntf3* mRNA. Ligands expressed only in the uninjured nerve are indicated by one asterisk, only in the 3 DPI nerve by two asterisks, and only in the 7 DPI nerve by one plus sign. Ligands expressed in the 3 and 7 DPI nerves but not in the uninjured nerve are indicated by two plus signs. Also shown in a separate column are ligands expressed in all populations (intersect).

* uninjured only.

** 3 DPI only.

^+^ 7 DPI only.

^++^ 3 DPI and 7 DPI intersect.

The combined 3 and 9 DPI nerve scRNA-seq dataset (Fig. 1*E*) was analyzed to identify the percentage of cells in each cell type expressing the ligand mRNAs identified by the microarray analysis. For this analysis, *Pdgfra*-positive mesenchymal cells were separated into endoneurial cells (cluster 5; Fig. 1*E*) and all other mesenchymal cells. Ligand mRNAs were considered further only if they were detectable in 2% or more cells of at least one cell type. The 143 resultant injured nerve ligands (Table 2) were further analyzed in the other scRNA-seq datasets. Venn diagrams comparing expression of the 143 injured nerve ligands in the various datasets (Figs. 2*G*, 3*E*, 4*C*) were prepared using the VennDiagram package, modified to show proportional representation with the euler*APE* tool (Micallef and Rodgers, 2014).

**Table 2:** Gene abundance analysis of ligand mRNAs in the combined 3 and 9 DPI nerve scRNA-seq dataset

	Gene abundance (%)						
Gene	Epineurial/perineurial	Endoneurial	Combinedmesenchymal	VSM/pericytes	Endothelialcells	Schwanncells	Immune
Adm^*^^*^	11.6	34.2	17.4	2.8	BT	BT	BT
Agt^*^	2.2	10.4	4.2	13.2	BT	BT	BT
Angpt1^*^^*^	17.3	23.7	18.9	21.1	2.1	BT	BT
Angpt2^*^	3.2	BT	2.6	29.0	18.5	BT	BT
Angpt4^**^	2.6	BT	2.0	BT	BT	BT	BT
Apln^*^	2.0	6.0	3.0	BT	11.2	BT	BT
Artn	BT	BT	BT	BT	BT	3.3	BT
Bdnf^*^	2.6	BT	2.3	2.2	BT	9.5	BT
Bmp1^**^	53.1	35.7	48.7	14.8	10.8	50.6	2.7
Bmp2^*^	BT	3.0	2.1	24.6	3.3	BT	BT
Bmp4^*^	2.8	BT	2.3	BT	16.6	BT	BT
Bmp5^*^	BT	3.8	BT	11.7	BT	BT	BT
Bmp7^**^	2.2	31.1	9.5	BT	BT	BT	BT
Btc^*^	BT	2.0	2.0	BT	BT	60.4	BT
Cck	BT	BT	BT	5.0	BT	BT	BT
Ccl11^**^	29.4	87.0	44.0	34.1	7.5	6.2	4.7
Ccl19	BT	BT	BT	2.5	BT	BT	BT
Ccl2^**^	27.0	81.5	40.9	21.1	12.9	13.8	15.2
Ccl24	BT	BT	BT	BT	BT	BT	2.4
Ccl25	BT	BT	BT	BT	BT	2.2	BT
Ccl3^*^	3.0	4.2	3.3	3.2	2.3	3.1	13.7
Ccl5^*^	3.0	2.8	2.9	BT	BT	BT	6.4
Ccl7^**^	21.8	78.0	36.1	10.4	7.7	6.6	7.7
Ccl9^**^	11.8	75.0	27.9	4.1	6.1	5.9	22.5
Clcf1^*^	3.7	4.5	3.9	BT	4.3	16.0	BT
Crlf1^*^	3.2	25.9	9.0	BT	BT	35.2	BT
Csf1^**^	32.3	31.2	32.0	13.9	11.7	11.6	3.3
Ctgf^**^	25.7	36.9	28.5	25.6	33.3	3.5	2.2
Cx3cl1^**^	4.0	14.4	6.7	BT	5.3	BT	BT
Cxcl1^**^	27.4	59.3	35.5	38.2	29.0	11.7	9.5
Cxcl10^**^	2.6	9.8	4.4	3.2	4.1	6.6	2.0
Cxcl12^**^	46.6	52.6	48.1	33.8	44.0	5.9	5.0
Cxcl13^**^	6.7	BT	5.1	BT	BT	BT	BT
Cxcl16^*^	6.3	4.2	5.7	2.8	3.7	BT	15.7
Cxcl2^*^	12.3	22.9	15.0	7.3	10.4	7.2	22.9
Cxcl9^**^	5.3	2.7	4.6	BT	4.6	BT	BT
Dhh	BT	BT	BT	BT	6.3	30.1	BT
Dll1	BT	BT	BT	BT	6.4	BT	BT
Dll4	BT	BT	BT	BT	10.0	BT	BT
Ebi3	BT	BT	BT	BT	BT	BT	2.5
Eda^**^	6.0	9.7	7.0	BT	BT	3.7	BT
Edn3^**^	3.1	BT	2.3	BT	BT	BT	BT
Efna1^*^	3.4	8.0	4.5	BT	24.9	BT	BT
Efna2^**^	3.3	3.8	3.4	2.5	BT	3.5	BT
Efna4^**^	4.8	6.2	5.2	3.2	BT	4.0	BT
Efna5^**^	5.1	6.7	5.5	BT	BT	BT	BT
Efnb1^**^	22.5	39.2	26.8	9.5	11.4	11.4	2.3
Efnb2^**^	13.0	42.4	20.4	4.1	16.4	4.0	BT
Fgf1^*^	7.1	4.8	6.5	9.8	BT	2.2	BT
Fgf10^**^	7.5	BT	5.8	BT	BT	BT	BT
Fgf18^**^	8.5	BT	6.8	BT	BT	BT	BT
Fgf5	BT	BT	BT	BT	BT	19.4	BT
Fgf7^**^	26.1	37.7	29.1	3.2	BT	12.5	BT
Figf^**^	15.8	11.2	14.6	BT	BT	BT	BT
Fstl1^**^	96.8	96.5	96.7	85.8	56.5	33.4	19.6
Gas6^**^	44.3	2.3	35.9	13.6	22.5	2.2	6.2
Gdf10^**^	24.5	4.7	19.5	BT	3.3	2.0	BT
Gdf11^**^	4.3	10.4	5.9	10.1	3.4	3.9	BT
Gdnf	BT	BT	BT	BT	BT	21.8	BT
Gmfb^**^	23.4	34.6	26.3	10.7	17.5	27.9	11.6
Gmfg	BT	BT	BT	BT	5.7	BT	17.9
Gnrh1^**^	BT	2.3	BT	BT	BT	BT	BT
Grp	BT	BT	BT	BT	3.4	BT	BT
Hbegf^*^	6.5	5.2	6.2	10.7	19.2	43.9	BT
Hgf^**^	4.0	BT	3.1	BT	BT	BT	BT
Igf1^**^	76.6	84.3	78.6	15.8	31.7	16.7	13.8
Igf2^*^	15.5	8.2	13.6	16.4	6.1	2.4	BT
Il15^**^	2.2	9.0	3.9	BT	7.4	BT	BT
Il16^*^	BT	3.0	BT	BT	2.3	BT	7.9
Il18^**^	6.4	5.3	6.1	BT	BT	BT	2.5
Il1b^*^	5.6	7.3	6.1	5.0	5.7	5.0	18.6
Il33^**^	39.1	63.3	45.3	2.5	3.6	6.4	2.2
Il6^*^	7.2	7.0	7.1	12.6	15.2	BT	BT
Inha^**^	BT	2.0	BT	BT	BT	BT	BT
Inhba^**^	12.6	11.9	12.4	9.8	BT	BT	1.9
Inhbb^**^	BT	12.7	4.5	BT	9.2	BT	BT
Jag1^*^	20.3	17.2	19.5	33.1	17.8	8.4	2.8
Jag2	BT	BT	BT	BT	8.4	BT	BT
Lif^**^	2.6	13.2	5.3	BT	BT	5.9	BT
Ltb	BT	BT	BT	BT	BT	BT	12.2
Mdk^**^	39.0	51.1	42.1	9.1	4.0	25.1	BT
Metrn^*^	7.1	14.5	9.0	8.5	3.0	57.8	2.6
Mif^**^	32.7	40.2	34.6	26.5	31.4	29.5	26.0
Nenf^**^	54.7	59.1	55.8	42.6	29.4	50.8	8.4
Ngf^*^	2.4	6.7	3.5	10.4	BT	BT	BT
Nov^**^	25.0	11.4	21.5	6.0	5.4	5.0	BT
Nppc^**^	2.2	5.3	3.0	BT	BT	BT	BT
Ntf3^*^	3.4	BT	2.9	4.4	4.8	BT	BT
Ntn1^**^	19.2	25.5	20.8	BT	6.0	4.6	BT
Osm	BT	BT	BT	BT	BT	BT	8.3
Pdgfa^*^	12.1	7.8	11.0	49.5	6.8	50.6	5.7
Pdgfb	BT	BT	BT	2.5	22.3	BT	2.4
Pdgfc^**^	7.2	BT	5.8	6.0	4.3	BT	BT
Pf4^*^	2.3	4.3	2.8	BT	BT	BT	10.0
Pgf^**^	2.0	12.2	4.6	7.6	BT	BT	BT
Pomc^**^	BT	3.3	BT	BT	3.3	2.2	BT
Pthlh^**^	10.4	13.7	11.2	BT	BT	2.4	BT
Ptn^*^	35.9	10.4	29.4	2.2	19.9	52.5	BT
Rspo1^**^	2.2	BT	BT	BT	BT	BT	BT
Rspo3^**^	6.0	BT	4.7	BT	2.4	BT	BT
Rtn4^*^	57.9	68.3	60.5	51.7	47.1	73.6	28.3
Sema3b^*^	7.7	12.4	8.9	BT	BT	53.8	BT
Sema3c^**^	35.4	9.0	28.7	BT	BT	25.1	BT
Sema3d^**^	9.6	2.0	7.6	BT	BT	BT	BT
Sema3e^*^	2.0	BT	BT	BT	BT	31.0	BT
Sema3f^*^	2.3	4.5	2.9	2.8	14.5	2.9	BT
Sema3g^*^	BT	3.0	BT	BT	11.8	9.0	BT
Sema4a^*^	BT	3.7	2.0	BT	BT	BT	6.9
Sema4b^*^	2.0	3.2	2.3	3.5	BT	7.7	BT
Sema4c^*^	8.4	12.0	9.3	6.3	10.2	16.0	BT
Sema4d	BT	BT	BT	BT	BT	BT	14.5
Sema4f	BT	BT	BT	BT	BT	18.3	BT
Sema5a^*^	4.3	3.5	4.1	13.2	BT	BT	BT
Sema5b	BT	BT	BT	10.4	BT	BT	BT
Sema6a^*^	4.0	14.5	6.7	BT	35.6	9.4	BT
Sema6b^*^	3.1	7.0	4.1	BT	18.1	BT	BT
Sema6c^**^	7.2	4.0	6.4	BT	BT	2.0	BT
Sema6d^*^	5.9	11.7	7.3	18.9	9.5	16.7	BT
Sema7a^*^	7.5	19.5	10.5	BT	17.4	20.0	BT
Sfrp1^**^	42.3	31.9	39.6	4.1	3.4	8.8	BT
Sfrp2^**^	29.6	6.5	23.8	2.2	3.3	BT	2.4
Sfrp4^**^	61.5	51.9	59.1	8.5	9.4	11.0	6.3
Sfrp5^**^	8.8	3.2	7.4	BT	2.1	8.1	BT
Shh	BT	BT	BT	BT	BT	12.8	BT
Tgfa	BT	BT	BT	BT	2.6	BT	BT
Tgfb1^*^	6.5	12.0	7.9	6.0	13.4	10.3	12.4
Tgfb2^*^	13.3	2.7	10.6	16.4	5.1	6.2	BT
Tgfb3^*^	26.3	8.7	21.8	27.4	2.3	4.6	BT
Tnf^*^	BT	2.2	BT	BT	BT	BT	7.7
Tnfrsf11b^**^	6.6	BT	5.0	BT	BT	BT	BT
Tnfsf10	BT	BT	BT	BT	22.8	1.1	BT
Tnfsf12^**^	15.0	16.7	15.4	11.4	9.1	6.8	7.3
Tnfsf14	BT	BT	BT	BT	BT	BT	2.3
Tnfsf8^**^	3.8	2.0	3.3	BT	BT	BT	BT
Tnfsf9^**^	2.8	5.8	3.6	5.4	BT	BT	BT
Tslp^**^	2.5	4.8	3.1	2.8	2.4	BT	BT
Ucn2	BT	BT	BT	BT	BT	15.0	BT
Vegfa^**^	28.2	18.7	25.8	3.8	4.1	2.6	7.0
Vegfb^*^	7.1	7.0	7.0	7.3	6.0	8.4	3.3
Vegfc^*^	2.0	BT	BT	BT	5.5	BT	BT
Wnt11^**^	2.7	BT	2.2	BT	BT	BT	BT
Wnt2^**^	6.5	BT	5.3	BT	BT	BT	BT
Wnt5a^**^	15.9	14.4	15.5	4.4	BT	BT	BT

The combined 3 and 9 DPI scRNA-seq dataset (Fig. 1*E*) was analyzed for the percentage of cells within the defined nerve cell types that expressed ligand mRNAs as identified by microarray analysis (Table 1). Ligand mRNAs were only considered to be detected if they were expressed in at least 2% of cells within at least one cell type. BT = below threshold and indicates that <2% of cells detectably expressed the ligand mRNA. Ligands annotated with one asterisk in the leftmost column were expressed in ≥2% *Pdgfra*-positive mesenchymal cells and two asterisks indicate ligands with the highest expression in either the epineurial/perineurial or endoneurial *Pdgfra*-positive mesenchymal cells.

^*^ >2% expression in *Pdgfra*-positive cells.

^**^ >2% expression and highest expression in *Pdgfra-*positive cells.

### RNA isolation and microarray analysis

Total RNA was extracted from E15 rat dorsal root ganglion (DRG) sensory neurons and neonatal rat superior cervical ganglion (SCG) sympathetic neurons using the RNeasy Micro kit (QIAGEN) according to manufacturer’s instructions. RNA was isolated from the distal rat sciatic nerve following injury (3 and 7 d after resection) and contralateral uninjured sciatic nerve as follows. After harvesting, nerves were flash frozen in liquid nitrogen and stored at −80°C until RNA isolation was performed. Nerve tissue was lysed using a Dounce homogenizer with cooled TRIzol (1 ml/50–100 mg of tissue) followed by passing the lysate through a 23.5-gauge needle. The homogenate was then spun at 12,000 × *g* at 4°C for 10 min, with the clear supernatant transferred to a new tube and allowed to incubate at room temperature for 5 min, 0.2 ml of chloroform was then added per milliliter of homogenate, shaken vigorously for 15 s, incubated 2–3 min at room temperature, and spun at 12,000 × *g* for 15 min at 4°C. The resulting aqueous phase was removed and added to a new tube with equal volume of 70% ethanol before RNA isolation with the RNeasy Micro kit (QIAGEN) according to manufacturer’s instructions. Microarray analysis was then performed at the Center for Applied Genomics at the Hospital for Sick Children (Toronto, ON). A total of 250 ng of total RNA was processed using the Affymetrix WT Plus kit to generate cDNA following Bioanalyzer analysis to confirm the quality of the RNA. 5.5 μg of labeled cDNA was hybridized onto Rat Gene 2.0 ST arrays using the Affymetrix FS450_0002 hybridization protocol and scanned using the Affymetrix GeneChip Scanner 3000.

### Normalization and differential gene expression analysis of microarray data

Raw probe intensity values were background corrected and then normalized with quantile normalization. Values were transformed into the log2 scale and summarized into probesets using the robust multichip analysis (RMA) algorithm in the oligo Bioconductor package in R. For all of the rat microarray datasets, gene annotation was performed using the “ragene20sttranscriptcluster.db” library in R. Motor neuron microarray data (Kaplan et al., 2014) was obtained from mouse P7 lumbar motor neurons from the GEO database under the accession number GSE52118. For these data, raw probe intensity values were normalized and summarized into probesets as described above, except for the Affymetrix Mouse Genome 430 2.0 Array (*n* = 3 replicates), and gene annotation was performed using the “mouse4302.db” library in R. The Limma Bioconductor package was used to calculate differential gene expression between the sensory neurons (DRGs, *n* = 6 replicates) and sympathetic neurons (SCGs, *n* = 4 replicates). Bayesian statistics were calculated and annotated receptor genes were considered to be differentially expressed if they were ≥2-fold different with *p* < 0.05 false discovery rate (FDR; Benjamini and Hochberg correction).

### Cell-surface capture mass spectrometry

Cell-surface mass spectrometry was conducted based on modified published protocols (McDonald et al., 2009; Schiess et al., 2009; Yuzwa et al., 2016). Briefly, 6-cm dishes containing sensory and sympathetic neurons were washed with coupling buffer [1× PBS (pH 6.5) and 0.1% fetal bovine serum (FBS)] following removal of the medium. Cultures were treated with 5 mm NaIO_4_ in coupling buffer for 30 min at room temperature in the dark and lysed in buffer containing 20 mm Tris-HCl, 150 mm NaCl, 0.0002% NaN_3_, 1% NP-40 (pH 7.5), and one complete mini-protease inhibitor tablet (Roche) per 10 ml of lysis buffer. Lysates were passed through 23-gauge needles, protein concentrations determined using the Pierce BCA assay kit (catalog #23227, Thermo Fisher Scientific), and equal amounts of total protein (between 0.9 and 1.5 mg for sensory neurons and <1 mg for sympathetic neurons) were added to 200 μl of Ultralink Hydrazide resin (Pierce) pre-equilibrated with lysis buffer and rotated overnight at room temperature. The unbound protein was removed via centrifugation the following day, washed twice with 8 m urea and three times with 50 mm ammonium bicarbonate (pH 8; ABC). The resin was treated with 50 mm dithiothreitol (DTT; American Bioanalytical) in ABC at 37°C for 60 min, washed once with ABC, and incubated with 65 mm iodacetamide in ABC at room temperature in the dark for 30 min. The resin was washed once with ABC, once with 1.5 m NaCl and three times with ABC and incubated with 40 ng/μl of Trypsin (Worthington) in ABC overnight at 37°C. The following day, the resin was washed three times with 1.5 m NaCl, followed by 80% acetonitrile, methanol, water, and ABC and then incubated with 1300 U/ml of PNGaseF (New England Biolabs) at 37°C overnight in ABC. The following day, the eluted peptides were collected from the resin and the resin washed once with ABC and combined with the eluted peptides. The eluates were lyophilized overnight and prepared for mass spectrometry using C18 reverse-phase ZipTips (EMD Millipore). Peptides were lyophilized and resuspended in 11 μl of 0.1% formic acid following elution from ZipTips. Samples were then analyzed on an Orbitrap analyzer (Q-Exactive, Thermo Fisher) outfitted with a nano-spray source and EASY-nLC nano-LC system (Thermo Fisher); 5 μl of the resuspended peptide mixtures were loaded onto a 75 μm × 50 cm PepMax RSLC EASY-Spray column filled with 2 μm C18 beads (Thermo Fisher) at a pressure of 800 Bar. Peptides were eluted over 60 min at a rate of 250 nl/min using a 0–35% acetonitrile gradient in 0.1% formic acid. Peptides were introduced by nano-electrospray into the Q-Exactive mass spectrometer (Thermo Fisher). The instrument method consisted of one MS full scan (400–1500 m/z) in the Orbitrap mass analyzer with an automatic gain control (AGC) target of 1e6, maximum ion injection time of 120 ms, and a resolution of 70,000 followed by 10 data-dependent MS/MS scans with a resolution of 17,500, an AGC target of 1e6, maximum ion time of 120 ms, and one microscan. The intensity threshold to trigger a MS/MS scan was set to 1.7e4. Fragmentation occurred in the HCD trap with normalized collision energy set to 26. The dynamic exclusion was applied using a setting of 8 s. Peptide and protein identification was performed using PEAKS version 8 software (Bioinformatics Solutions Inc.). Peptides and proteins were identified at the 0–0.1% FDR level in the sensory neuron dataset and at the 0.8–1% FDR level in the sympathetic neuron dataset. With both sensory and sympathetic neuron culture datasets, data were pooled across the three samples, and we included all proteins where at least a single peptide was detected in at least one of the samples.

### Identification of receptors based on the microarray and mass spectroscopy data

**Figure 5.**
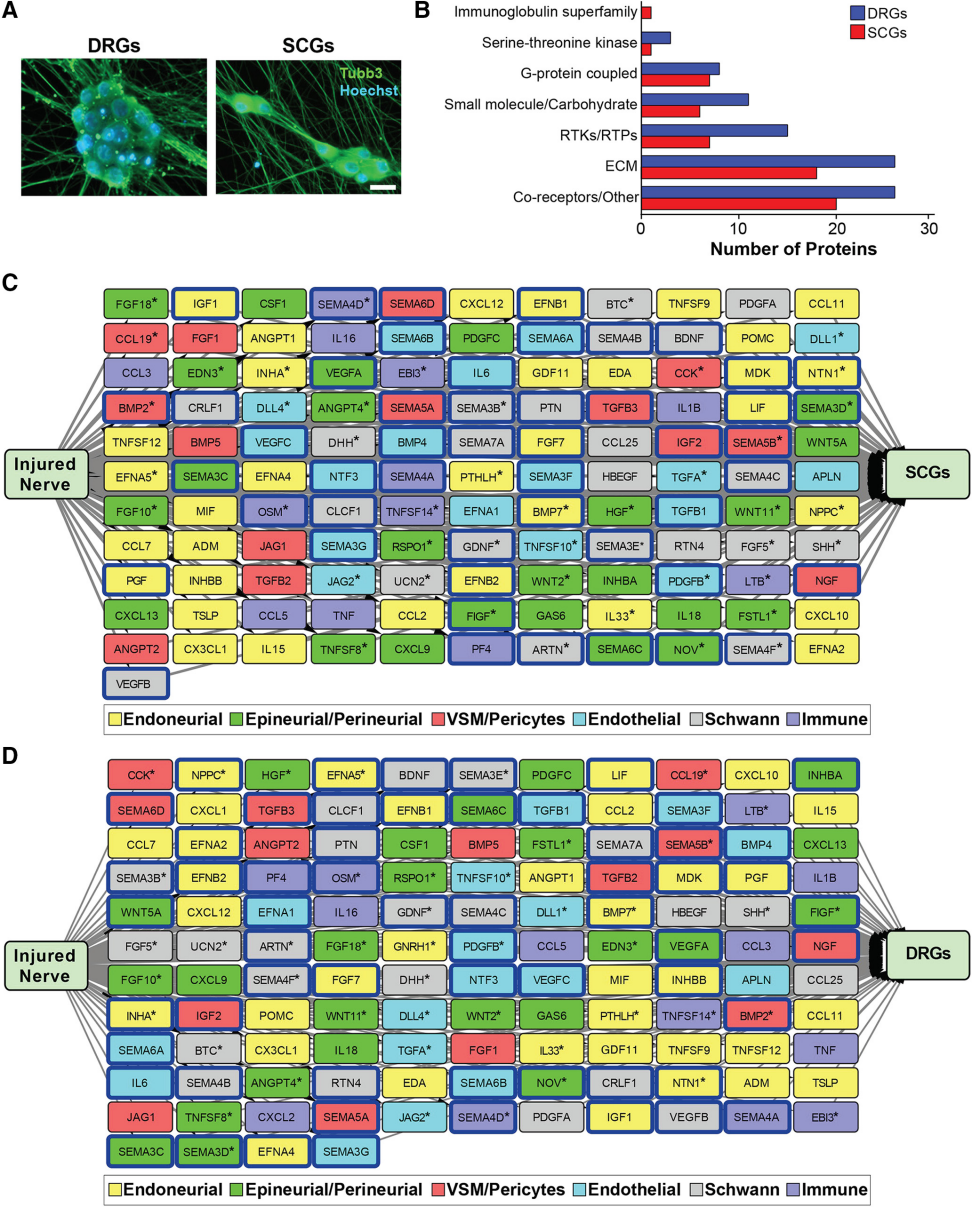
Sensory and sympathetic neuron receptor expression and modeling of their predicted interactions with injured nerve-derived ligands (see also Extended Data Fig. 5-1). ***A***, Images of E15 rat DRG sensory neurons cultured for 9 d (left panel) and neonatal rat SCG sympathetic neurons cultured for 6 d (right panel), immunostained for βIII-tubulin (Tubb3; green). Cells were counterstained with Hoechst 33258 to show cell nuclei (blue). Scale bars = 20 μm. ***B***, Bar graphs showing types of receptor proteins detected on the surface of sensory and sympathetic neurons following cell-surface capture mass spectrometry. Classification of receptors was based on GO terms identified via the UniProt database (http://uniprot.org) as well as manual curation of all detected cell-surface proteins. RTKs/RTPs = receptor tyrosine kinases and receptor tyrosine phosphatases; ECM = extracellular matrix. ***C***, ***D***, Models showing predicted unidirectional paracrine interaction networks between the 143 injured nerve ligands and receptors expressed by sympathetic (***C***, SCGs) and sensory (***D***, DRGs) neurons, as defined at the transcriptional and proteomics levels. Interactions were predicted by computational modeling using the ligand-receptor database, and then manually curated for well-validated interactions. Nodes represent ligands that are color coded to identify the injured nerve cell type with the highest expression of the ligand mRNA based on the scRNA-seq analysis. Predicted interactions where the receptors were detected at the protein level are indicated by a blue box around the corresponding ligand node. Asterisks indicate ligands that were expressed at 4-fold higher levels in the indicated cell type as compared with all other injured nerve cell types. Arrows indicate directionality of interactions.

Receptors were identified in both the microarray and mass spectrometry data using the ligand-receptor database described in Yuzwa et al. (2016), with modifications. In addition, proteins that were classified as receptors using the PANTHER (http://pantherdb.org) protein classification system, but not necessarily present in the ligand-receptor database, were included. Manual curation of receptors with known signaling functions (such as the Plxn receptors) as well as other genes identified as receptors by Gene Ontology (GO) terms were also included as an update to the previously published version of the ligand-receptor database. Receptor classifications (Fig. 5*B*) were based on GO terms and descriptions provided by websites including GeneCards (http://genecards.org) and UniProt (http://uniprot.org).

10.1523/ENEURO.0066-20.2020.f5-1 Extended Data Figure 5-1 Identification of cell-surface proteins on sensory and sympathetic neurons. ***A***, Bar graphs showing the percentage of cells expressing the neuronal protein βIII-tubulin (Tubb3), the Schwann cell protein S100β, or the fibroblast protein Fibronectin in cultures of DRG sensory neurons or SCG sympathetic neurons as shown in Figure 5*A*. The total number of cells in the cultures was determined by counterstaining with Hoechst 33258. Values: mean ± SEM, *n* = 6 for DRGs, *n* = 4 for SCGs except for cultures immunostained for Fibronectin where *n* = 2. ***B***, Venn diagram showing the overlap of cell-surface proteins detected by mass spectrometry in sensory neurons and sympathetic neurons. All proteins included were annotated by the terms “cell membrane” and/or “secreted” by the UniProtKB database (http://uniprot.org). ***C***, Bar graphs showing classification of the proteins detected by cell-surface capture mass spectrometry on sensory neurons (DRGs, blue) and sympathetic neurons (SCGs, red). Proteins were classified as receptors based on the ligand-receptor database, GO terms in the PANTHER classification system, as well as by manual curation, and were further classified into receptor types as shown in Figure 5*B*. The remainder of the graph includes proteins classified using PANTHER (http://pantherdb.org). ***D***, Graphs showing the distribution of proteins detected by cell-surface capture mass-spectrometry relative to their transcript expression levels (based on microarray analyses described in the text) in sensory neurons (DRGs, left) and sympathetic neurons (SCGs, right). The cutoffs used to define receptor expression in the microarray data were based on the receptors detected by mass spectrometry analysis that had the lowest levels of mRNA expression. This was *Itgam* for sensory neurons (DRGs) and *Sorcs3* for sympathetic neurons (SCGs, shown in red). Download Figure 5-1, TIF file.

### Computational modeling and pre-processing of data

To define receptors for generation of ligand-receptor models, gene expression values from the injured nerve and from the motor, sensory, and sympathetic neuron microarrays were first averaged across replicates, and in the case of multiple probes corresponding to the same receptor gene, the highest expressed probe was used (this was following averaging). For rat retinal ganglion cells (RGCs), we analyzed RNA-seq data from Blanco-Suarez et al. (2018) as obtained from the GEO database (accession GEO:GSE108484). Data from control RGCs were used (*n* = 3) and processed as described above for the microarray data. Expression values with fragments per kilobase of transcript per million mapped reads (FPKM) >1 were considered expressed and included for analysis. For the modeling, receptor mRNAs that had expression values exceeding the thresholds as described in the results were included. These receptors and the 143 injured nerve ligands (Table 2) were then analyzed using a custom Python script (“Cellcellinteractnet,” Python version 2.7.6) and custom ligand-receptor database (database modified from Yuzwa et al., 2016) to predict ligand-receptor interactions. Models were all constructed from this information using Cytoscape (3.7.0). The Venn diagram comparing predicted interactions in Figure 7*C* was prepared using the VennDiagram package, modified to show proportional representation with the euler*APE* tool (Micallef and Rodgers, 2014).

### Sympathetic neuron cultures

SCGs of newborn (P1–P2) Sprague Dawley rats were dissected, dissociated and cells were plated at eight ganglia per 3.5-cm tissue culture-treated dish for microarrays, ∼1 × 10^6^ cells per 6-cm dish for mass spectrometry, or ∼1.4 × 10^5^ cells per 13-mm glass coverslip for immunostaining. Neurons were plated on dishes and coverslips coated with poly-d-lysine and laminin (1 μg/ml laminin; VWR). Neurons were cultured in growth medium composed of UltraCULTURE medium (Lonza), 2 mm l-glutamine (Lonza), 100 U/ml penicillin (Wisent) with 100 μg/ml streptomycin (pen/strep; Wisent), and 50 ng/ml NGF (Cedarlane). Neurons were cultured in growth medium containing 3% heat-inactivated FBS for 3 d with inclusion of 7.2 μm cytosine arabinoside (CA) for the first day, and 3.6 μm CA for the second and third days. Cultures were switched to growth medium alone for an additional 3 d, as described previously (Park et al., 2010; Feinberg et al., 2017).

### Sensory neuron cultures

Sensory neurons from E15 rat DRGs were cultured as described (Feinberg et al., 2010, 2017). For immunostaining, neurons were plated at a density of 2 × 10^5^ cells per 13-mm glass coverslip precoated with laminin (VWR), and poly-d-lysine (Sigma-Aldrich). Neurons were plated at a density of 1 × 10^6^ cells per 6-cm dish for proteomics. For microarrays, neurons were plated under both conditions. Initially, cells were plated in a medium #1 containing Neurobasal medium (Invitrogen), GlutaMAX (Invitrogen), 50 ng/ml NGF (Cedarlane), B27 supplement (Invitrogen), and pen/strep. The day after plating, cultures were treated for 2 d in medium #2 composed of Eagles basal medium (Invitrogen), ITS supplement (Sigma), 0.2% BSA (Sigma), 4 mg/ml d-glucose (Sigma), GlutaMAX (Invitrogen), 50 ng/ml NGF (Cedarlane), and pen/strep with 0.8 μm CA to eliminate mitotic non-neuronal cells. Cells were then treated with another cycle of medium #1 for 2 d followed by a final 2-d cycle of medium #2. After the second CA treatment, cultures were grown in medium #1 for two additional days before cell harvest.

### Culturing and sorting mesenchymal cells from the sciatic nerve for ELISA analysis

Sciatic nerves were harvested from P4 Sprague Dawley rat pups. Each biological sample included four litters of pups (two litters harvested per day over 2 d). Nerve cells from two litters were dissociated and plated on 6-cm dishes in medium consisting of low-glucose DMEM/F12 (3:1, Invitrogen) and 1% pen/strep (referred to as basal medium) with 10% FBS. In order to isolate PDGFRα-positive mesenchymal cells, 1–2 d after plating, cells were treated with an antibody solution containing basal medium with goat anti-PDGFRα antibody (1:250, R&D Systems, catalog #AF1062) and donkey anti-goat Alexa Fluor 488 secondary (1:500, Thermo Fisher Scientific, catalog #A11055) that was preincubated for 30 min at room temperature. Cells were treated for 1 h at 37°C with the antibody solution before being returned to basal medium with 10% FBS overnight. PDGFRα-positive cells were then isolated using a Mo-Flo XDP sorter (Beckman Coulter). Following the sort, between 4–9 × 10^5^ cells were re-plated on 6-cm dishes with basal medium and 10% FBS. Approximately 72 h later, medium containing FBS was removed, cells were washed with HBSS, and basal medium without serum was added for conditioning. Cells were treated with cycles of basal medium for conditioning (24–96 h) and basal medium with FBS for a period of 2.5–4 weeks total. Collected conditioned medium was spun down at 1300 rpm for 5 min to removed cell debris and frozen at −80°C until ELISAs were performed. ELISA assays for rat ANGPT1 (catalog #LS-F10827, LSBio), CCL11 (catalog #LS-F11046, LSBio), and VEGFC (catalog #LS-F5482, LS Bio) were performed and results were analyzed according to manufacturer’s instructions.

### Sympathetic neuron compartment cultures

Campenot cultures were established on 35-mm collagen-coated dishes as previously reported (Campenot et al., 1991); 20 × 20 mm Teflon chambers (Tyler Research) were grease-sealed and assembled on plates where the substratum was corrugated with 20 parallel tracks 200 μm wide using a pin rake (CAMP-PR, Tyler Research), forming lanes for outgrowth. Neonatal SCG sympathetic neurons were enzymatically and mechanically dissociated and plated in the central chamber at a density of three to four ganglia per compartment in 10 ng/ml NGF (Cedarlane, catalog #CLMCNET-001.1), methylcellulose Ultraculture media (Lonza) supplemented with 2 mm L-glutamine (Lonza), and antibiotics [100 U/ml penicillin (Wisent) and 100 μg/ml streptomycin (Wisent)]; 10 μm CA with 3% FBS was added to the cell bodies for 2–3 d as the axons were permitted to extend into the adjacent compartments containing 10 ng/ml NGF. After this period, central and side chambers were washed with fresh medium, medium in the cell body chamber was replaced with 10 ng/ml NGF, and medium on the axonal chambers was replaced with 100 ng/ml candidate ligand and varying amounts of NGF (0.5 ng/ml for experimental compartments, 50 ng/ml for maximum NGF control). Ligands included human recombinant ANGPT1 (Peprotech, catalog #130-06-5UG), murine recombinant Eotaxin (CCL11, Peprotech, catalog #250-01-5UG), and human recombinant VEGFC (Peprotech, catalog #100-20CD). Images of axon outgrowth were obtained at 3d post-ligand addition using a Zeiss AxioObserver Z1 microscope and outgrowth was analyzed using ImageJ software (NIH). To quantify the density of axonal growth in these compartments, a line was drawn perpendicular to the axis of the outgrowth within the furthest 1 mm of outgrowth where axons were maximally defasciculated and the number of axons crossing the line were quantified.

### Immunostaining, imaging, and analysis of cultures

Immunostaining of cultured cells was performed on glass coverslips and tissue culture dishes. Cells were fixed for ∼10 min in 4% paraformaldehyde in PBS solution and washed three times (10 min per wash) in PBS before treatment with the primary antibody solution, which contained the antibody, 10% normal donkey or normal goat blocking serum (Jackson ImmunoResearch), and 0.3% Triton X-100 (Fisher) in PBS. After 1–2 h of incubation at room temperature, cells were washed three times (10 min per wash) with PBS. Cells were then incubated in a secondary antibody solution containing antibodies diluted 1:500 in 0.3% Triton X-100 (Fisher) in PBS. After 1 h, cells were then washed three times (10 min per wash) in PBS with Hoechst 33258 (Sigma) added in one of the washes to label nuclei. For glass slides, coverslips were mounted with PermaFluor mounting media (Thermo Fisher Scientific). For tissue culture dishes, mounting medium was added directly on to the cells, and coverslips were placed on top of this. Cells were imaged using either a Zeiss AxioImager M2 microscope with an X-Cite 120 LED light source and a C11440 Hamamatsu camera using Zen acquisition software in the case of glass coverslips, or with a Zeiss AxioObserver Z1 microscope with Zen software in the case of tissue culture dishes. To quantify cells, multiple regions per glass coverslip or dish were imaged and cells were counted. For purified sensory and sympathetic neuron culture quantifications, S100β-positive and βIII-tubulin-positive cells were counted in four regions per coverslip across two coverslips per biological replicate and summed before calculating percent positive immunoreactive cells. Fibronectin-positive cells were counted in three regions over two sympathetic neuron replicates. Total cell counts per biological replicate ranged from ∼100 to ∼600. In cases where cells in dishes were counted, between three and eight regions per dish were used. Images were then processed using Photoshop software (Adobe Systems Incorporated), where brightness and contrast were edited as appropriate.

### Immunostaining of sections

Adult *Pdgfra^EGFP/+^* mice underwent unilateral sciatic nerve injury and injured distal sciatic nerve and uninjured contralateral sciatic nerve tissue was harvested at 9 DPI. Nerves were fixed overnight in 4% paraformaldehyde at 4°C, then cryoprotected overnight in 30% sucrose at 4°C. Nerves were then cryosectioned longitudinally at 12 μm and immunostained for PDGFRα, S100β and counterstained with Hoechst 33258 (Sigma). Images were acquired with a 20× objective lens on a Zeiss AxioImager M2 microscope with a light source, camera, and software setup as described above.

### Antibodies

The following primary antibodies were used in this study at the indicated dilutions: rabbit anti-Fibronectin (1:500; Sigma; catalog #F3648), goat anti-PDGFRα (1:250 for cultures and 1:500 for sections; R&D Systems, catalog #AF1062), rabbit anti-S100β (1:500; Dako; catalog #Z0311), and mouse anti-βIII-tubulin (1:1000; BioLegend; catalog #801202). The following secondary antibodies were used in this study (all from Thermo Fisher Scientific and used at a dilution of 1:500, or two drops/ml in the case of the ReadyProbe): donkey anti-goat IgG Alexa Fluor 647 (catalog #A21447), donkey anti-mouse IgG Alexa Fluor 488 (catalog #A21202), donkey anti-mouse IgG ReadyProbes Alexa Fluor 488 (catalog #R37114), donkey anti-rabbit IgG Alexa Fluor 488 (catalog #A21206), and donkey anti-rabbit IgG Alexa Fluor 555 (catalog #A31572).

### FISH

Single molecule FISH was performed on 9 DPI adult *Pdgfra^EGFP/+^* nerve sections with probes targeting *Vegfc* (catalog #492701), *Ccl11* (catalog #464031), and *Angpt1* (catalog #449271) mRNAs using the RNAscope kit (Advanced Cell Diagnostics), according to the manufacturer’s instructions. Briefly, freshly dissected nerves were fixed overnight in 4% PFA at 4°C and cryopreserved in 30% sucrose overnight (at 4°C) under RNase-free conditions using RNase-free reagents. Nerves were cryosectioned sagittally at a thickness of 14 μm. Sections were washed with ethanol, followed by tissue pretreatment (1:10 dilution) for 20 min, probe hybridization, and signal amplification. Positive signal was identified as red punctate dots. Digital images were acquired with a Quorum Spinning-Disk confocal microscope system using Volocity acquisition software (PerkinElmer). Z-stack confocal images were taken with an optical slick thickness of 0.3 μm, and projected Z-stacked images are shown. The scrambled probe provided with the RNAscope kit was used as a negative control.

### Statistical analyses

With the exception of analyses related to the microarray and scRNA-seq data, all statistics were performed using GraphPad Prism 8 (GraphPad Software). Standard deviation values are given for the genes and transcripts per cell values for the scRNA-seq data. Scatter plots show standard error of the mean. Statistical significance was considered reached at *p* < 0.05. All statistical tests and conditions are described in the text. Graphs were generated in both GraphPad Prism 8 and Excel (Microsoft).

### Data/software accessibility

Raw scRNA-seq and microarray datasets have been deposited in the GEO database under the ID codes GEO:GSE147285 and GSE146958. The Cellcellinteractnet code, the updated ligand-receptor database, and the raw proteomics data are available on request.

## Results

### Defining growth factors made within the injured and uninjured peripheral nerve

We hypothesized that peripheral nerves promote axon growth in part due to ligand secretion by resident nerve *Pdgfra*-positive mesenchymal cells. To test this idea, we analyzed the sciatic nerve, which contains axons from sensory, sympathetic and motor neurons. Initially, we confirmed that *Pdgfrα*-positive mesenchymal cells are found within the nerve endoneurium where axons are located. To do this, we analyzed control and injured sciatic nerve sections from mice carrying a transgene where EGFP is driven by *Pdgfra* regulatory sequences, and which thus tags *Pdgfra*-positive mesenchymal cells (*Pdgfra^Egfp/+^* mice; Hamilton et al., 2003). We resected sciatic nerves from these mice, and at 9 DPI immunostained for the PDGFRα protein product and for the Schwann cell marker S100β. For comparison, we performed a similar analysis of the contralateral uninjured sciatic nerve. This analysis identified many *Pdgfra*-EGFP-positive, PDGFRα protein-positive cells within the endoneurium of both the control and injured nerves (Fig. 1*A*), consistent with a similar recent analysis in Carr et al. (2019).

Having confirmed the presence of many mesenchymal cells in the endoneurium, we obtained an overview of ligands expressed in the sciatic nerve by performing global transcriptomic analysis of rat sciatic nerves that were either uninjured or that were injured by resection 3 or 7 d earlier. In all cases, we isolated total RNA from four independent biological replicates of the distal nerve segment and analyzed samples on Affymetrix GeneChip Rat Gene 2.0 ST Arrays. To define ligand mRNAs, we used a curated database of secreted ligands and their cognate receptors (Yuzwa et al., 2016), establishing an expression cutoff by considering only mRNAs expressed at similar or higher levels than neurotrophin 3 (*Ntf3*) mRNA, which is expressed in and important for peripheral nerves. *Ntf3* mRNA was expressed in the top 67.6%, 70.3%, and 71.2% of mRNAs in control, 3-d injured, and 7-d injured distal nerves, respectively. This analysis identified 238, 249, and 258 ligand mRNAs in the uninjured, 3-d injured, and 7-d injured nerves, respectively (Fig. 1*B*; Table 1). Most (226) were detected in all three conditions, but 31 ligand mRNAs were found only in the injured nerves, including *Areg*, *Ccl20*, *Il6*, and *Wnt7a* and five ligands were detected only in the control nerve, including *Il21* (Table 1).

### Single-cell profiling defines similarities between peripheral nerves at 3 and 9 DPI

The microarray analysis defined the global nerve ligand environment. To ask which nerve cells express these ligands, we performed single-cell RNA sequencing (scRNA-seq), initially analyzing the distal sciatic nerve 3 d following a nerve transection. To do this, we dissociated the distal transected sciatic nerve, removed myelin debris with myelin-removal beads, and sequenced cells using high-throughput, droplet-based scRNA-seq (for details of all sequencing runs, see Materials and Methods). To analyze these individual transcriptomes, we then used a pipeline that incorporates extensive low-level data quality analysis with visualization and clustering methods that use evidence-based parameter selection (Innes and Bader, 2019), as previously described (Yuzwa et al., 2017; Carr et al., 2019; Storer et al., 2020). Genes with high variance were then used to compute principal components as inputs for projecting cells in two dimensions using t-SNE followed by graph-based clustering (Butler et al., 2018) with a range of resolution parameters.

This analysis defined nine distinct clusters containing 2075 cells in the 3-d injured sciatic nerve dataset (Fig. 1*C*). To assign cell types to these different clusters, we used well-characterized marker genes (for all cell type-specific marker genes used, see Materials and Methods; Fig. 1*D*; Extended Data Fig. 1-1*A*). This approach identified *Pecam1*/*Cd31*-positive endothelial cells, *Sox10*-positive Schwann cells, *Aif1*/*Iba1*-positive macrophages, *Trbc2*-positive immune cells, and *Rgs5*-positive VSM and pericyte cells. As previously seen in the 9-d injured sciatic nerve (Carr et al., 2019), we also identified distinct populations of *Pdgfra*-positive nerve mesenchymal cells, including *Dpp4*-positive epineurial cells (clusters 1, 4, and 6) and *Wif1*-positive endoneurial cells (cluster 3).

We next asked whether distal sciatic nerve cells were similar at 3 and 9 d following a transection, taking advantage of a recently published 9 DPI scRNA-seq analysis where nerve cells were also isolated using myelin beads (Carr et al., 2019). To do this, we extracted the relevant raw transcriptomes from the previously published 9 DPI dataset, combined them with the 3 DPI raw transcriptomes we had generated, and put this combined 5395 cell dataset through our computational pipeline, which corrects for any differences in sequencing depth and library size. As seen with the 3 DPI dataset alone, marker gene analysis (Fig. 1*E*; Extended Data Fig. 1-1*B*,*C*) identified clusters containing endothelial cells, VSM/pericyte cells, Schwann cells, macrophages, immune cells, and *Pdgfra*-positive mesenchymal cells. These mesenchymal cells included *Etv1*-positive endoneurial cells (cluster 5), *Pcolce2*-positive epineurial cells (cluster 3), *Msln*-positive perineurial cells (cluster 10), and differentiating nerve bridge cells enriched for *Dlk1* (cluster 1; Fig. 1*E*; Extended Data Fig. 1-1*B*,*C*). Analysis of the dataset of origin (Fig. 1*F*) showed that all cell type clusters were comprised of intermingled 3 and 9 DPI cells except for cluster 7, which included *Cd19*-positive B cells that came almost exclusively (99.4%) from the 9 DPI time point (Extended Data Fig. 1-1*B*). Similar results were obtained following batch correction. Thus, 3- and 9-d distal transected sciatic nerve cells are transcriptionally similar, although there is an increased proportion of B cells at 9 d.

### Schwann cells, mesenchymal cells, vasculature, and immune cells make distinct contributions to the peripheral nerve ligand environment

To understand how the different cell types contributed to the injured nerve environment, we analyzed the combined 3- and 9-d scRNA-seq dataset for expression of ligands identified in the microarray analysis (Fig. 1*B*; Table 1), excluding extracellular matrix proteins and ligands without well-defined, receptor-mediated paracrine roles. This analysis identified 143 ligands that were detectably expressed in ≥ 2% of at least one nerve cell type (Table 2), including many well-known nerve ligands such as the neurotrophins NGF, BDNF, and NT3, the Ret family ligands GDNF, Artemin and Neurturin, DHH, and various Semaphorin and FGF family members. Notably, *Pdgfra*-positive mesenchymal cells detectably expressed more ligand mRNAs than any other nerve cell type (118/143, or 82.5%; Fig. 1*G*; Table 2). Of these, 71 were expressed, proportionately, in more mesenchymal cells than any other cell type (Fig. 1*H*; Table 2), and some were highly mesenchymally enriched, including *Adm*, *Bmp7*, *Cxcl13*, *Fgf7*, *Fgf10*, *Gdf10*, *Hgf*, *Il33*, *Ntn1*, *Pthlh*, and *Wnt5a* (Fig. 1*I*; Extended Data Fig. 1-1*D*). A total of 39 of these mesenchymal ligands were most highly expressed in the endoneurial mesenchymal cells that are closely apposed to Schwann cells and axons (Fig. 1*G*; Table 2).

Schwann cells were a second major source of injured nerve ligands, detectably expressing 74 ligand mRNAs (Fig. 1*G*,*H*; Table 2). A total of 23 of these were expressed in more Schwann cells than any other cell type, including *Artn*, *Bdnf*, *Btc*, *Clcf1*, *Crlf1*, *Dhh*, *Fgf5*, *Gdnf*, *Hbegf*, *Sema3e*, *Sema4f*, *Shh*, and *Ucn2* (Fig. 2*A*; Extended Data Fig. 2-1*A*; Table 2). Of the other cell types, endothelial and VSM/pericyte cells expressed 18 and 15 ligand mRNAs, respectively, at the highest levels (Fig. 1*G*,*H*; Table 2). These included *Bmp4* and *Pdgfb* mRNAs in endothelial cells and *Bmp2* and *Ngf* in VSM/pericytes (Fig. 2*B*). The immune cells expressed 16 ligand mRNAs in the highest proportions including well-characterized immune ligands like *Osm* and *Tnf* (Figs. 1*G*,*H*, 2*B*; Table 2). Thus, ligands known to be important for axon growth and tissue regeneration are expressed by diverse nerve cell populations after injury.

### Single-cell transcriptional profiling of uninjured and developing nerves

We asked whether this cellular profile of ligand expression was exclusive to the injured nerve by analyzing single-cell transcriptional datasets of the uninjured and developing sciatic nerves. For the uninjured nerve, we used a dataset that was previously analyzed for *Pdgfra*-positive mesenchymal cells (Carr et al., 2019), but not for other cell types. Analysis of the entire 1841 uninjured nerve cell dataset (Fig. 2*C*,*D*; Extended Data Fig. 2-1*B*) identified clusters comprised of epineurial, perineurial, and endoneurial mesenchymal cells, as well as VSM/pericyte cells, endothelial cells, and a small population (1.9%) of immune cells. There were also two *Sox10*-positive Schwann cell populations; non-myelinating cells expressing *Ngfr/p75NTR*, *Cdh2*, and *L1cam*, and myelinating Schwann cells expressing high levels of *Mbp*, *Pmp22*, and *Plp*. This latter population was likely relatively reduced in numbers because myelin removal beads were used when isolating the nerve cells. There were very few proliferating cells, as indicated by expression of cell cycle genes like *Top2a* and *Ki67* (Extended Data Fig. 2-1*B*).

For the developing neonatal nerve, we generated a new dataset, sequencing P2–P4 sciatic nerve cells after isolating them using either flow cytometry or myelin removal beads in two separate preparations. We combined raw transcriptomes from both preparations and analyzed them together. This analysis, which included 6885 total cells, identified 10 clusters containing intermingled cells from both preparations (Fig. 2*E*; Extended Data Fig. 2-1*C*,*D*). This intermingling was unaffected by batch correction (Extended Data Fig. 2-1*C*). Cell type-specific marker genes identified endothelial cells, VSM/pericyte cells, macrophages, and epineurial, endoneurial, and perineurial mesenchymal cells (Fig. 2*E*,*F*; Extended Data Fig. 2-1*D*). At this age, there were five *Sox10*-positive Schwann cell clusters, with one cluster (6) containing proliferative Schwann cells and another (9) myelinating Schwann cells expressing high levels of *Mag*, *Mbp*, *Mpz*, and *Pmp22* (Fig. 2*E*,*F*).

Analysis of ligand mRNA expression in these datasets showed that cells in the uninjured and developing sciatic nerves expressed many but not all injured nerve ligands. Specifically, of 143 total ligands, 122 and 119 were detectably expressed in uninjured and neonatal nerves, respectively, and 111 were shared in all three conditions (Fig. 2*G*; Tables 3, 4). These ligand mRNAs were contributed by all of the different cell types in the uninjured and neonatal nerves (Fig. 2*H*,*I*). However, the pattern of ligand expression differed from the injured nerve (compare Figs. 2*I*, 1*H*), with endoneurial mesenchymal cells and Schwann cells contributing relatively fewer ligands in the developing and uninjured nerves (endoneurial cells, 11% and 27% in the uninjured vs injured nerves; Schwann cells, 5% and 16% in the uninjured vs injured nerves). Moreover, 13 injured nerve ligands were not detectably expressed in the neonatal or uninjured nerves (*Artn*, *Btc*, *Ccl25*, *Crlf1*, *Fgf5*, *Gdnf*, *Gnrh1*, *Hgf*, *Rspo3*, *Sema4f*, *Shh*, *Tnfsf8*, and *Ucn2*; Tables 3, 4). Notably, all of these were injured nerve Schwann cell or mesenchymal cell ligands (Table 2). Thus, multiple cell types contribute to the sciatic nerve ligand environment in all conditions, but mesenchymal and Schwann cells become relatively more important following injury.

**Table 3:** Gene abundance of injured nerve ligand mRNAs in the uninjured nerve scRNA-seq dataset

	Gene abundance (%)						
Gene	Epineurial/perineurial	Endoneurial	VSM/pericytes	Endothelial	Schwann(non-myelinating)	Schwann(myelinating)	Immune
Adm^**^	14.4	8.4	BT	3.6	BT	BT	2.9
Agt^**^	2.9	BT	BT	BT	BT	BT	2.9
Angpt1^*^	12.2	BT	12.4	BT	BT	BT	BT
Angpt2^*^	2.2	BT	21.1	13.6	BT	6.0	BT
Angpt4^**^	6.2	BT	BT	BT	BT	BT	2.9
Apln	BT	BT	BT	7.1	BT	BT	BT
Bdnf	BT	BT	3.1	BT	BT	BT	BT
Bmp1^**^	42.0	16.1	6.8	6.2	4.6	4.8	BT
Bmp2^*^	3.5	BT	15.5	BT	BT	BT	11.4
Bmp4^**^	18.6	6.5	BT	17.6	BT	BT	BT
Bmp5^*^	2.4	6.8	8.1	BT	BT	BT	BT
Bmp7^**^	3.1	11.9	BT	BT	BT	BT	BT
Cck	BT	BT	3.1	BT	BT	BT	BT
Ccl11^**^	35.8	88.3	22.4	9.0	5.3	3.6	2.9
Ccl19^**^	6.2	BT	5.0	BT	BT	BT	BT
Ccl2^*^	2.2	16.1	4.3	BT	BT	BT	28.6
Ccl3	BT	BT	BT	BT	BT	BT	11.4
Ccl5	BT	BT	BT	BT	BT	BT	11.4
Ccl7^**^	6.0	35.5	2.5	5.0	2.7	3.6	8.6
Ccl9^**^	BT	16.6	BT	BT	BT	2.4	14.3
Clcf1	BT	BT	BT	4.0	BT	BT	2.9
Csf1^**^	31.9	10.0	4.3	15.2	8.8	2.4	8.6
Ctgf^*^	16.6	2.1	10.6	28.8	BT	BT	BT
Cx3cl1	BT	BT	BT	11.2	BT	2.4	BT
Cxcl1^**^	17.5	52.3	18.6	15.0	11.5	6.0	8.6
Cxcl10	BT	BT	BT	BT	BT	BT	5.7
Cxcl12^*^	34.3	60.0	39.8	78.1	13.4	3.6	2.9
Cxcl13^**^	16.2	BT	BT	BT	BT	BT	BT
Cxcl16^**^	10.0	BT	BT	3.3	BT	BT	2.9
Cxcl2	BT	BT	BT	BT	BT	BT	8.6
Cxcl9	BT	BT	BT	3.8	BT	BT	BT
Dhh	BT	BT	BT	3.8	13.4	28.9	BT
Dll1	BT	BT	BT	10.0	BT	BT	BT
Dll4	BT	BT	BT	18.1	BT	BT	BT
Eda^**^	5.1	7.7	BT	BT	3.4	2.4	2.9
Edn3^**^	8.4	BT	BT	BT	BT	BT	BT
Efna1^*^	4.2	4.0	3.7	30.2	BT	BT	BT
Efna2	BT	BT	BT	BT	BT	BT	2.9
Efna5^**^	4.4	BT	BT	BT	BT	BT	BT
Efnb1^**^	9.5	14.3	2.5	10.5	6.1	3.6	2.9
Efnb2^*^	3.5	11.0	4.3	15.7	BT	BT	BT
Fgf1^*^	4.9	BT	10.6	BT	10.3	26.5	2.9
Fgf10^**^	9.1	BT	BT	BT	BT	BT	BT
Fgf18^**^	8.4	BT	BT	BT	BT	BT	BT
Fgf7^**^	28.8	6.3	2.5	2.4	BT	8.4	BT
Figf^**^	12.8	BT	BT	3.1	BT	BT	BT
Fstl1^**^	90.9	75.5	47.2	34.5	29.8	10.8	8.6
Gas6^**^	61.5	9.3	18.0	49.8	3.8	6.0	2.9
Gdf10^**^	39.4	4.0	BT	3.1	BT	2.4	2.9
Gdf11^*^	BT	4.2	4.3	BT	BT	BT	2.9
Gmfb^*^	14.2	8.6	5.6	10.5	7.6	15.7	5.7
Gmfg	BT	BT	BT	3.1	BT	BT	28.6
Hbegf^*^	BT	2.3	12.4	17.4	25.2	8.4	5.7
Igf1^**^	56.9	43.9	8.1	17.6	2.7	BT	BT
Igf2^*^	3.5	8.6	7.5	14.3	BT	BT	BT
Il15^*^	BT	4.7	BT	5.0	BT	BT	BT
Il16	BT	BT	BT	3.1	BT	8.4	25.7
Il18^**^	7.1	3.7	BT	BT	BT	BT	BT
Il1b	BT	BT	BT	BT	BT	BT	8.6
Il33^**^	29.4	71.5	BT	5.0	3.4	2.4	2.9
Il6^*^	2.4	2.8	4.3	5.2	BT	BT	BT
Inha^**^	2.0	BT	BT	BT	BT	BT	BT
Inhba	BT	BT	8.1	BT	BT	BT	BT
Jag1^*^	12.4	4.2	20.5	19.3	BT	BT	BT
Jag2	BT	BT	BT	9.5	BT	BT	BT
Lif^**^	BT	3.0	BT	BT	BT	BT	2.9
Ltb	BT	BT	BT	BT	BT	BT	11.4
Mdk^**^	8.2	16.4	BT	BT	3.4	BT	2.9
Metrn^*^	2.7	BT	2.5	BT	14.5	12.0	2.9
Mif^*^	14.8	14.7	19.3	31.0	13.7	18.1	31.4
Nenf^**^	54.2	48.6	28.0	31.0	26.7	19.3	17.1
Ngf^*^	BT	2.6	11.2	BT	BT	BT	BT
Nov^**^	19.2	7.9	6.8	5.0	7.3	15.7	BT
Nppc^**^	BT	2.6	BT	BT	BT	BT	BT
Ntf3^*^	4.0	BT	11.8	5.7	BT	BT	BT
Ntn1^**^	31.2	12.6	BT	6.7	BT	BT	BT
Osm	BT	BT	BT	BT	BT	BT	8.6
Pdgfa^*^	7.3	BT	32.9	9.8	14.1	16.9	5.7
Pdgfb	BT	BT	3.1	28.1	BT	BT	BT
Pdgfc^**^	3.3	BT	BT	BT	BT	BT	2.9
Pgf^*^	2.4	4.7	7.5	BT	BT	BT	BT
Pomc	BT	BT	BT	5.0	BT	BT	2.9
Pthlh^**^	12.6	7.2	BT	BT	BT	BT	BT
Ptn^*^	7.7	4.0	BT	45.7	56.5	BT	5.7
Rspo1^**^	8.4	BT	BT	BT	BT	BT	BT
Rtn4^**^	37.6	33.4	21.7	33.8	24.0	21.7	11.4
Sema3b^*^	15.5	32.7	3.1	3.1	44.3	56.6	BT
Sema3c^**^	38.1	9.1	2.5	3.6	9.2	2.4	BT
Sema3d^**^	13.7	BT	BT	BT	BT	BT	BT
Sema3e^*^	3.5	BT	BT	BT	14.5	BT	BT
Sema3f	BT	BT	2.5	12.6	BT	BT	BT
Sema3g^*^	BT	3.0	BT	18.8	2.7	2.4	BT
Sema4a	BT	BT	BT	BT	BT	BT	5.7
Sema4b^**^	2.0	2.1	BT	BT	BT	BT	BT
Sema4c^*^	6.6	6.3	BT	11.2	5.7	22.9	BT
Sema4d	BT	BT	BT	BT	2.3	BT	8.6
Sema5a^*^	2.7	3.5	6.8	BT	BT	9.6	BT
Sema6a^*^	2.7	18.7	BT	26.2	2.7	2.4	2.9
Sema6b^*^	2.0	2.8	BT	12.4	BT	BT	2.9
Sema6c^*^	BT	3.3	BT	BT	BT	7.2	BT
Sema6d^*^	3.3	3.5	8.1	9.5	25.2	13.3	BT
Sema7a	BT	BT	BT	28.8	8.4	BT	2.9
Sfrp1^**^	45.4	15.0	2.5	4.0	6.9	BT	BT
Sfrp2^**^	45.1	5.1	BT	5.2	3.8	6.0	5.7
Sfrp4^**^	61.7	8.6	3.1	9.8	5.3	6.0	8.6
Sfrp5^*^	18.8	5.8	3.7	4.5	26.0	31.3	5.7
Tgfa	BT	BT	BT	4.0	BT	BT	BT
Tgfb1^*^	2.0	3.3	BT	7.9	BT	BT	17.1
Tgfb2^**^	8.2	2.8	5.6	6.0	BT	BT	BT
Tgfb3^**^	16.8	2.3	16.1	3.1	BT	BT	BT
Tnf	BT	BT	BT	BT	BT	BT	11.4
Tnfsf10	BT	BT	BT	18.6	BT	BT	BT
Tnfsf12^**^	13.3	17.8	6.2	12.6	9.9	9.6	5.7
Tnfsf14	BT	BT	BT	BT	BT	BT	8.6
Tnfsf9^*^	BT	4.0	BT	BT	5.7	BT	BT
Tslp^**^	3.5	BT	BT	BT	BT	BT	2.9
Vegfa^**^	14.2	7.9	8.1	6.2	BT	2.4	2.9
Vegfb^*^	5.5	4.7	6.2	5.7	3.4	7.2	BT
Vegfc^*^	3.3	BT	BT	6.2	BT	BT	BT
Wnt11^**^	6.6	BT	BT	BT	BT	BT	2.9
Wnt2^**^	6.9	BT	BT	BT	BT	BT	BT
Wnt5a^**^	17.5	10.3	BT	BT	BT	BT	BT

The uninjured nerve scRNA-seq dataset (Fig. 2*C*) was analyzed to determine the percentage of cells within a given cell type that detectably expressed (≥2%) the 143 injured nerve ligand mRNAs (Table 2). Cell populations were defined as for the injured nerve analysis except that Schwann cells were divided into myelinating (cluster 8) and non-myelinating (cluster 4) cells. BT = below threshold and indicates that <2% of cells detectably expressed the ligand mRNA. Ligands annotated with one asterisk in the leftmost column were expressed in ≥2% *Pdgfra*-positive mesenchymal cells and two asterisks indicate ligands with the highest expression in either the epineurial/perineurial or endoneurial *Pdgfra*-positive mesenchymal cells.

^*^ >2% expression in *Pdgfra*-positive cells.

^**^ >2% expression and highest expression in *Pdgfra-*positive cells.

**Table 4:** Gene abundance of injured nerve ligand mRNAs in the neonatal nerve scRNA-seq dataset

	Gene abundance (%)					
Gene	Epineurial	Endoneurial/perineurial	VSM/pericytes	Endothelial	Schwann cells	Immune cells
Adm^**^	5.1	4.7	BT	3.0	BT	BT
Agt^*^	2.5	BT	12.2	BT	BT	BT
Angpt1^*^	5.1	BT	11.1	BT	BT	BT
Angpt2^*^	5.5	BT	18.1	6.9	2.0	BT
Apln	BT	BT	BT	29.0	BT	BT
Bdnf	BT	BT	3.6	BT	BT	BT
Bmp1^**^	34.2	21.5	10.9	11.0	10.8	BT
Bmp2	BT	BT	6.7	3.4	BT	8.7
Bmp4^*^	3.4	BT	BT	6.9	BT	BT
Bmp5	BT	BT	13.4	BT	BT	BT
Bmp7^**^	BT	3.9	BT	BT	BT	BT
Ccl11^**^	9.4	49.8	20.0	BT	BT	2.6
Ccl2^*^	BT	10.0	9.9	BT	BT	20.0
Ccl24	BT	BT	BT	BT	BT	27.0
Ccl3	BT	BT	BT	BT	BT	16.5
Ccl5	BT	BT	BT	BT	BT	2.6
Ccl7^**^	3.4	18.5	2.7	BT	BT	14.8
Ccl9	BT	BT	BT	BT	BT	28.7
Clcf1	BT	BT	BT	2.3	BT	BT
Csf1^**^	15.2	4.3	4.8	4.1	2.4	BT
Ctgf^*^	7.1	4.0	7.4	7.4	BT	BT
Cxcl1^*^	BT	12.4	20.6	2.5	BT	2.6
Cxcl10	BT	BT	BT	BT	2.6	BT
Cxcl12^**^	18.9	37.2	23.1	33.8	BT	BT
Cxcl16	BT	BT	BT	4.2	BT	11.3
Cxcl2	BT	BT	BT	BT	BT	4.3
Cxcl9^**^	BT	2.1	BT	BT	BT	BT
Dhh	BT	BT	2.3	7.1	25.6	3.5
Dll1	BT	BT	BT	5.3	BT	BT
Dll4	BT	BT	BT	15.2	BT	BT
Ebi3	BT	BT	BT	BT	BT	13.0
Eda^**^	7.1	9.0	BT	BT	4.4	BT
Edn3^**^	4.1	3.5	BT	BT	BT	BT
Efna1^*^	3.1	4.3	BT	34.0	BT	BT
Efna2^**^	2.9	3.3	BT	BT	2.9	BT
Efna4^**^	2.6	3.6	BT	BT	BT	BT
Efna5^**^	6.0	4.0	BT	BT	2.3	BT
Efnb1^**^	18.3	30.6	6.3	9.7	9.1	2.6
Efnb2^**^	5.4	15.1	4.8	16.3	BT	BT
Fgf1^**^	5.1	5.6	3.6	BT	5.4	3.5
Fgf10^**^	5.7	BT	BT	BT	BT	BT
Fgf18^**^	7.8	BT	BT	BT	BT	BT
Fgf7^**^	21.0	10.3	BT	BT	BT	BT
Figf^**^	3.8	4.8	BT	BT	BT	BT
Fstl1^**^	96.0	92.2	72.9	59.3	43.0	11.3
Gas6^*^	22.4	14.3	7.4	24.6	BT	45.2
Gdf10^**^	8.4	BT	BT	BT	BT	BT
Gdf11^**^	BT	6.6	3.8	2.7	BT	BT
Gmfb^**^	9.5	17.6	6.1	13.8	14.1	9.6
Gmfg	BT	BT	BT	6.4	BT	36.5
Grp	BT	BT	BT	2.1	BT	BT
Hbegf^*^	BT	2.5	14.3	15.0	19.2	3.5
Igf1^**^	56.0	52.0	4.6	16.6	2.0	40.0
Igf2^**^	92.3	89.5	54.0	69.7	11.1	25.2
Il15	BT	BT	BT	BT	BT	4.3
Il16	BT	BT	BT	4.6	BT	12.2
Il18	BT	BT	BT	BT	BT	9.6
Il1b	BT	BT	BT	BT	BT	3.5
Il33^**^	13.7	16.1	BT	BT	BT	BT
Inha^**^	2.6	BT	BT	BT	BT	BT
Inhba^**^	4.1	BT	BT	BT	BT	BT
Inhbb	BT	BT	8.0	BT	BT	BT
Jag1^**^	18.4	19.1	15.5	11.0	BT	BT
Jag2	BT	BT	BT	7.1	BT	BT
Ltb	BT	BT	BT	BT	BT	2.6
Mdk^**^	52.3	68.1	20.2	8.8	20.9	8.7
Metrn^*^	3.4	8.7	10.5	5.0	43.2	10.4
Mif^*^	17.5	26.4	28.8	26.0	32.8	26.1
Nenf^**^	54.3	54.6	39.7	28.8	24.5	BT
Ngf^**^	BT	8.7	8.0	BT	BT	0.0
Nov^**^	23.8	4.3	3.8	3.4	2.8	3.5
Nppc^**^	BT	6.1	BT	BT	BT	BT
Ntf3	BT	BT	2.3	BT	BT	BT
Ntn1^**^	22.2	10.0	BT	BT	BT	BT
Osm	BT	BT	BT	BT	BT	3.5
Pdgfa^*^	3.4	3.6	30.5	3.0	17.8	BT
Pdgfb	BT	BT	2.9	21.9	BT	BT
Pdgfc	BT	BT	3.2	BT	2.2	2.6
Pf4	BT	BT	BT	BT	BT	61.7
Pgf	BT	BT	3.8	BT	BT	BT
Pthlh^**^	7.1	4.0	BT	BT	BT	BT
Ptn^*^	29.3	13.9	6.5	15.9	43.4	4.3
Rspo1^**^	7.8	6.5	BT	BT	BT	BT
Rtn4^*^	31.0	38.2	35.7	40.4	26.8	38.3
Sema3b^*^	5.4	6.5	2.3	2.8	34.7	7.8
Sema3c^**^	51.7	19.2	BT	BT	7.1	3.5
Sema3d^**^	10.4	15.9	BT	BT	BT	BT
Sema3f^*^	2.6	BT	BT	2.8	BT	BT
Sema3g	BT	BT	BT	9.6	7.5	BT
Sema4a	BT	BT	BT	BT	BT	10.4
Sema4b	BT	BT	BT	BT	BT	3.5
Sema4c^*^	5.1	5.7	2.7	8.1	8.5	5.2
Sema4d	BT	BT	BT	BT	BT	7.8
Sema5a^*^	5.4	5.5	12.4	BT	BT	BT
Sema5b^*^	4.4	8.6	14.1	BT	2.6	BT
Sema6a^*^	4.4	7.9	BT	22.8	4.5	BT
Sema6b	BT	BT	BT	2.5	BT	BT
Sema6c^**^	4.9	3.3	2.5	BT	BT	BT
Sema6d^*^	2.0	3.0	4.4	6.4	16.7	8.7
Sema7a	BT	BT	2.1	17.3	2.0	BT
Sfrp1^**^	50.5	48.5	2.9	4.1	28.0	3.5
Sfrp2^**^	17.2	BT	BT	BT	BT	BT
Sfrp4^**^	43.7	5.5	BT	BT	BT	2.6
Sfrp5^**^	5.4	30.7	BT	BT	8.1	2.6
Tgfb1	BT	BT	BT	10.3	BT	20.9
Tgfb2^*^	12.1	2.7	18.3	9.9	BT	BT
Tgfb3^**^	21.5	8.3	10.9	BT	BT	BT
Tnf	BT	BT	BT	BT	BT	6.1
Tnfrsf11b^*^	2.9	BT	BT	3.9	BT	BT
Tnfsf10	BT	BT	BT	6.4	BT	BT
Tnfsf12^**^	8.0	10.8	5.5	6.5	3.2	4.3
Tnfsf9	BT	BT	BT	BT	BT	4.3
Tslp	BT	BT	2.7	2.8	BT	3.5
Vegfa^**^	16.0	3.6	3.8	3.2	BT	BT
Vegfb^**^	4.0	7.7	6.3	5.5	4.3	6.1
Vegfc	BT	BT	BT	8.5	BT	BT
Wnt11	BT	BT	2.7	BT	BT	BT
Wnt2^**^	7.5	BT	BT	BT	BT	BT
Wnt5a^**^	17.0	6.8	BT	BT	BT	BT

The neonatal nerve scRNA-seq dataset (Fig. 2*E*) was analyzed to determine the percentage of cells within a given cell type that detectably expressed (≥2%) the 143 injured nerve ligand mRNAs (Table 2). Cell populations were defined as for the injured nerve analysis. BT = below threshold and indicates that <2% of cells detectably expressed the ligand mRNA. Ligands annotated with one asterisk in the leftmost column were expressed in ≥2% *Pdgfra*-positive mesenchymal cells and two asterisks indicate ligands with the highest expression in either the epineurial/perineurial or endoneurial *Pdgfra*-positive mesenchymal cells.

^*^ >2% expression in *Pdgfra*-positive cells.

^**^ >2% expression and highest expression in *Pdgfra-*positive cells.

### Injured Schwann cells acquire a unique transcriptional phenotype following injury including upregulation of many growth factor genes

To ask about the apparent injury-associated increase in ligand expression, we analyzed the Schwann cells and *Pdgfra*-positive mesenchymal cells in more detail. We first combined transcriptomes from all Schwann cell clusters in the neonatal, uninjured, 3 DPI, and 9 DPI nerve datasets [cluster 6 (Fig. 1*E*), clusters 4 and 8 (Fig. 2*C*), clusters 1, 2, 4, 6, and 9 (Fig. 2*E*)]. We augmented this combined dataset by including Schwann cells from the FAC-sorted 9 DPI dataset from Carr et al. (2019). Once this combined dataset was put through the pipeline, we used the Harmony batch correction method (Korsunsky et al., 2019) to correct for any technical variation. Analysis of this combined dataset indicated that injured nerve Schwann cells were distinct from both developing and adult uninjured Schwann cells. Specifically, the combined dataset included 5331 Schwann cells in seven clusters (Fig. 3*A*,*B*). The differentiating neonatal Schwann cells were present in clusters 0, 1, and 2 with proliferating cells in cluster 2 (Fig. 3*A*,*B*; Extended Data Fig. 3-1*A*). Adult and neonatal myelinating Schwann cells were present in clusters 5 and 6, while the adult uninjured non-myelinating Schwann cells were in cluster 4. By contrast, almost all injured nerve Schwann cells were present in cluster 3.

To better understand these clusters, we performed hierarchical and correlation analyses of average gene expression (Fig. 3*C*). These analyses confirmed that the injured Schwann cells were distinct from the other populations, and indicated that they were more similar to the differentiating neonatal cells (*r* = 0.88 for the comparison between clusters 3 and 0) than to the adult non-myelinating Schwann cells (*r* = 0.76 for the comparison between clusters 3 and 4). To understand these similarities and differences at an individual cell level, we performed single-cell correlation analysis. As comparators for the analysis, we determined average gene expression for uninjured non-myelinating versus neonatal non-myelinating Schwann cells and for uninjured non-myelinating versus 9 DPI Schwann cells. We then compared each single-cell transcriptome with the averaged bulk transcriptomes using Pearson’s correlation and used the resultant correlation coefficients to assign a two-dimensional coordinate to each cell. This analysis (Fig. 3*D*) showed that (1) virtually all 3 and 9 DPI Schwann cells were more similar to the neonatal Schwann cells than to the uninjured non-myelinating cells, (2) most neonatal Schwann cells were more similar to the injured cells than to the uninjured non-myelinating cells, and (3) despite these similarities, there was very little direct overlap between the injured and neonatal cells.

These data indicate that following nerve injury Schwann cells become more like neonatal Schwann cells, but that they are nonetheless distinct. In this regard, it has been reported that this unique injury state might involve acquisition of mesenchymal-like characteristics (Arthur-Farraj et al., 2017; Clements et al., 2017). To explore this idea further, we compared injured nerve Schwann cells and *Pdgfra*-positive mesenchymal cells from the combined 3 and 9 DPI nerve dataset (Fig. 1*E*). Correlation analysis showed that the injured Schwann cells were very distinct from both endoneurial and epineurial cells in the injured nerve (Extended Data Fig. 3-1*B*; *r* = 0.74–0.78). Thus, after injury, Schwann cells acquire a unique transcriptional profile that is similar but not identical to neonatal Schwann cells.

We asked about ligand gene expression in this combined dataset. This analysis showed that nerve injury led to upregulation of a subset of ligand mRNAs in Schwann cells. Specifically, in the combined Schwann cell dataset, 82 of the 143 injured nerve ligands were detectably expressed, but only 28 of these were common to the neonatal, uninjured, and injured Schwann cells (Fig. 3*E*; Table 5). Notably, 36 ligand mRNAs were expressed in ≥3-fold more injured versus uninjured non-myelinating cells (Table 5), with some almost exclusive to the injured cells, including *Artn*, *Bdnf*, *Btc*, *Ccl2*, *Ccl3*, *Clcf1*, *Crlf1*, *Cxcl2*, *Fgf5*, *Gdnf*, *Lif*, *Sema4f*, *Shh*, *Tgfb1*, and *Ucn2* mRNAs (Fig. 3*F*,*G*; Extended Data Fig. 3-1*C*). Other ligand mRNAs were also upregulated following injury but were still expressed by other Schwann cell populations, such as *Bmp1*, *Fgf7*, *Igf1*, and *Pdgfa* (Fig. 3*G*,*H*; Extended Data Fig. 3-1*D*). By contrast, some ligand mRNAs were expressed to some degree in all or most Schwann cell populations, including, for example, *Dhh*, *Mdk*, and *Fgf1* (Fig. 3*H*,*I*; Extended Data Fig. 3-1*D*). Thus, injured nerve Schwann cells acquire a unique, development-like transcriptional state that includes upregulation of growth factors implicated in nerve development, nerve regeneration, and tissue repair, including, for example, GDNF (Trupp et al., 1995; Naveilhan et al., 1997), BDNF (Lindsay, 1988; Leibrock et al., 1989), and PDGFα (Johnston et al., 2016).

**Table 5:** Gene abundance of injured nerve ligand mRNAs in the combined injured, uninjured and neonatal Schwann cell scRNA-seq dataset

	Gene abundance (%)				Fold change
Gene	Neonatal	Uninjured (myelinating)	Uninjured (non-myelinating)	Injured	Injured:uninjured (non-myel.)
Angpt2	2.0	6.4	BT (0.4)	BT (1.7)	4.1
Artn	BT (0.0)	BT (0.0)	BT (0.0)	3.1*	>3.1
Bdnf	BT (0.0)	BT (0.0)	BT (0.0)	9.3*	>9.3
Bmp1	10.9	3.8	4.9	46.1*	9.3
Btc	BT (0.8)	BT (0.0)	BT (0.0)	58.8*	>58.8
Ccl11	BT (0.8)	3.8	4.5	5.6*	1.2
Ccl2	BT (0.7)	BT (0.0)	BT (1.6)	12.7*	7.7
Ccl3	BT (0.0)	BT (0.0)	BT (0.0)	3.9*	>3.9
Ccl7	BT (0.2)	2.6	2.9	6.1*	2.1
Ccl9	BT (0.0)	2.6	BT (0.4)	6.6*	16.1
Clcf1	BT (0.2)	BT (0.0)	BT (0.0)	18.6*	>18.6
Crlf1	BT (1.3)	BT (0.0)	2.5	30.5*	12.4
Csf1	2.4	BT (1.3)	9.1	9.7*	1.1
Ctgf	BT (0.1)	BT (0.0)	BT (0.0)	3.4*	>3.4
Cx3cl1	BT (0.0)	2.6	BT (0.0)	BT (0.2)	-
Cxcl1	BT (1.4)	6.4	11.9	10.7	0.9
Cxcl10	2.6	BT (0.0)	BT (0.4)	5.9*	14.4
Cxcl12	BT (1.4)	2.6	12.3	5.4	0.4
Cxcl2	BT (0.1)	BT (0.0)	BT (0.0)	7.1*	>7.1
Dhh	25.3	28.2	11.9	26.8	2.2
Eda	4.4	2.6	3.7	2.9	0.8
Efna2	2.8	BT (1.3)	BT (0.8)	3.2*	3.9
Efna4	BT (1.0)	BT (1.3)	BT (0.0)	4.9*	>4.9
Efna5	2.2	BT (0.0)	BT (0.0)	BT (0.3)	-
Efnb1	8.9	2.6	7.0	10.0*	1.4
Efnb2	BT (0.6)	BT (0.0)	BT (0.4)	3.9*	9.5
Fgf1	5.4	28.2	10.7	BT (1.5)	0.1
Fgf5	BT (0.0)	BT (0.0)	BT (0.0)	18.3*	>18.3
Fgf7	BT (1.3)	9.0	BT (0.8)	12.9*	15.7
Fstl1	42.6	9.0	30.9	29.0	0.9
Gas6	BT (0.6)	6.4	2.9	2.5	0.9
Gdf11	BT (1.2)	BT (0.0)	BT (0.4)	3.1*	7.4
Gdnf	BT (0.0)	BT (0.0)	BT (0.0)	20.5*	>20.5
Gmfb	14.2	16.7	7.0	21.9*	3.1
Hbegf	19.0	9.0	26.7	39.0*	1.5
Igf1	2.1	BT (1.3)	3.3	15.4*	4.7
Igf2	11.2	BT (1.3)	BT (1.2)	3.6	2.9
Il16	BT (0.7)	7.7	BT (0.4)	BT (0.3)	0.8
Il18	BT (0.3)	BT (0.0)	2.1	BT (0.5)	0.2
Il1b	BT (0.0)	BT (0.0)	BT (0.0)	3.7*	>3.7
Il33	BT (0.2)	2.6	2.9	4.2*	1.5
Jag1	BT (0.9)	BT (1.3)	BT (1.2)	6.9*	5.6
Lif	BT (0.0)	BT (0.0)	BT (0.0)	4.9*	>4.9
Mdk	20.6	BT (1.3)	3.3	25.3*	7.7
Metrn	42.8	11.5	14.0	53.6*	3.8
Mif	32.5	17.9	11.9	26.1	2.2
Nenf	24.3	19.2	27.2	47.3*	1.7
Nov	2.7	14.1	7.8	4.2	0.5
Ntn1	BT (0.6)	BT (0.0)	BT (0.8)	3.2*	3.9
Pdgfa	17.5	15.4	15.6	47.5*	3.0
Pdgfb	BT (0.2)	BT (0.0)	BT (0.0)	2.2*	>2.2
Pdgfc	2.2	BT (1.3)	BT (1.2)	BT (1.0)	0.8
Ptn	42.9	BT (0.0)	57.2	47.3	0.8
Rtn4	26.7	21.8	24.3	70.7*	2.9
Sema3b	34.4	56.4	43.2	51.4	1.2
Sema3c	7.1	BT (1.3)	9.5	19.5*	2.1
Sema3e	BT (1.0)	BT (0.0)	14.4	29.2*	2.0
Sema3f	BT (0.4)	BT (0.0)	2.1	2.9*	1.4
Sema3g	7.5	2.6	BT (1.6)	8.1*	4.9
Sema4b	BT (0.4)	BT (0.0)	BT (0.8)	7.1*	8.6
Sema4c	8.5	21.8	5.3	15.4	2.9
Sema4d	BT (0.9)	BT (0.0)	2.1	BT (1.0)	0.5
Sema4f	BT (0.0)	BT (0.0)	BT (0.0)	17.1*	>17.1
Sema5a	BT (0.9)	10.3	BT (0.0)	BT (1.9)	>1.9
Sema5b	2.6	BT (0.0)	BT (0.0)	BT (0.2)	-
Sema6a	4.5	2.6	2.9	8.0*	2.8
Sema6c	BT (1.3)	7.7	BT (1.2)	2.0	1.6
Sema6d	16.5	11.5	25.5	14.1	0.6
Sema7a	2.1	BT (0.0)	9.5	17.1*	1.8
Sfrp1	27.6	BT (0.0)	7.0	8.0	1.1
Sfrp2	BT (0.4)	5.1	3.7	2.0	0.5
Sfrp4	BT (1.3)	6.4	4.9	8.8*	1.8
Sfrp5	8.1	30.8	28.0	5.1	0.2
Shh	BT (0.0)	BT (0.0)	BT (0.0)	12.0*	>12.0
Tgfb1	BT (0.9)	BT (1.3)	BT (0.8)	9.8*	11.9
Tgfb2	BT (0.6)	BT (0.0)	BT (0.8)	6.1*	7.4
Tgfb3	BT (1.8)	BT (1.3)	BT (1.6)	4.2*	2.6
Tnfsf12	3.2	9.0	9.5	6.3	0.7
Tnfsf9	BT (0.6)	BT (1.3)	5.3	BT (0.7)	0.1
Ucn2	BT (0.1)	BT (0.0)	BT (0.0)	14.1*	>14.1
Vegfa	BT (1.1)	2.6	BT (0.8)	2.4	2.9
Vegfb	4.2	7.7	3.3	7.5	2.3

The combined Schwann cell scRNA-seq dataset (Fig. 3*A,B*) was analyzed to determine the percentage of cells within the different Schwann cell clusters that detectably expressed (≥2%) the 143 injured nerve ligand mRNAs (Table 2). Also shown is the difference, expressed as fold change, in the percentage of positive cells in the injured versus uninjured, non-myelinating Schwann cell clusters. BT = below threshold and indicates that <2% of cells detectably expressed the ligand mRNA. Also shown are the absolute values, since these were used to determine the fold changes. Neonatal includes cells in clusters 0, 1, 2, and 5, uninjured myelinating includes cluster 6 cells, uninjured non-myelinating includes cluster 4 cells, and injured includes cluster 3 cells. Asterisks indicate ligand mRNAs with the highest expression in the injured Schwann cell cluster.

*Injured, highest expression.

### Upregulation of ligands in endoneurial mesenchymal cells following nerve injury

We performed a similar analysis of nerve mesenchymal cells, combining transcriptomes from the neonatal, uninjured and injured nerve *Pdgfra*-positive clusters [clusters 1, 3, 5, and 10 (Fig. 1*E*), clusters 1, 2, 6, and 10 (Fig. 2*C*), clusters 3 and 5 (Fig. 2*E*)], as well as the *Pdgfra*-positive mesenchymal transcriptomes of the FAC-sorted 9 DPI cells from Carr et al. (2019). Once this combined dataset was put through the pipeline, we used Harmony batch correction (Korsunsky et al., 2019) to correct for any technical variation. Analysis of this combined dataset showed that, as published previously (Carr et al., 2019), some mesenchymal populations were transcriptionally altered by injury, while others were largely unaffected. Specifically, the combined dataset included 5416 cells in nine *Pdgfra*-positive clusters (Fig. 4*A*,*B*). The injured and uninjured epineurial cells were coclustered, as were the injured, uninjured, and neonatal perineurial cells (Fig. 4*A*,*B*; Extended Data Fig. 4-1). By contrast, the uninjured, injured, and neonatal endoneurial cells were all segregated from each other. The other segregated clusters included neonatal epineurial cells (cluster 1) and the injured nerve differentiating bridge cells (cluster 0).

We used this combined dataset to ask about injury-induced ligand induction in mesenchymal cells. This analysis indicated that the endoneurial mesenchymal cells were largely responsible for this induction. Specifically, 102 of the 143 injured nerve ligands were detectably expressed in endoneurial mesenchymal cells (Fig. 4*C*), and, of these, 49 were expressed in at least 3-fold more injured versus uninjured cells, with 26 detectably expressed only in the injured cells (Table 6). These upregulated ligand mRNAs included *Angpt1*, *Ccl9*, *Crlf1*, *Cxcl2*, *Inhbb*, *Lif*, *Sema7a*, and *Ngf* (Fig. 4*D*,*E*; Table 6). In addition to this endoneurial cell response, some ligands were highest in the injured bridge cells, such as *Bdnf*, *Cxcl9*, and *Hgf* (Fig. 4*D*; Table 6). By contrast, many ligand mRNAs were expressed to a greater or lesser degree in all nerve mesenchymal cell populations regardless of nerve injury, including for example *Adm*, *Bmp1*, *Ccl11*, *Cxcl12*, *Il33*, *Pthlh*, *Fgf18*, *Pdgfa*, *Tgfb3*, *Vegfa*, and *Wnt5a* (Fig. 4*F*,*G*). Thus, injury induces expression of many ligand mRNAs in endoneurial mesenchymal cells, but many ligands are also expressed under homeostatic conditions in uninjured nerve mesenchymal cells.

**Table 6:** Gene abundance of injured nerve ligand mRNAs in the combined injured, uninjured and neonatal mesenchymal cell scRNA-seq dataset

	Gene abundance (%)								
	Neonatal		Inj/uninjured	Injured/uninjured/neonatal	Uninjured	Injured		Neonatal/injured	Fold change
Gene	Epineurial	Endoneurial	Epineurial	Perineurial	Endoneurial	Endoneurial	Differentiating	Proliferating	Injured:uninjuredendoneurial
Adm	5.2	4.8	17.3	17.0	9.0	35.1	7.6	10.1	3.9
Agt	BT (1.7)	BT (0.5)	3.3	BT (0.0)	BT (1.9)	10.7	BT (1.7)	3.9	5.6
Angpt1	3.7	BT (0.5)	11.6	5.2	BT (0.7)	23.9	24.6	20.8	33.6
Angpt2	3.4	BT (0.0)	2.0	BT (1.3)	BT (0.5)	BT (1.0)	5.1	2.9	2.0
Angpt4	BT (0.5)	BT (0.0)	4.9	BT (1.3)	BT (0.2)	BT (0.4)	2.0	2.6	1.7
Apln	BT (0.7)	2.1	BT (0.5)	BT (0.7)	BT (0.7)	5.3	BT (1.9)	6.5	7.5
Bdnf	BT (0.6)	BT (1.1)	BT (0.2)	BT (0.7)	BT (0.0)	BT (1.9)	5.2	BT (1.6)	>1.9
Bmp1	34.3	12.7	51.4	52.9	16.4	37.2	52.3	47.6	2.3
Bmp2	BT (0.2)	BT (0.0)	2.9	BT (0.7)	BT (0.9)	3.1	BT (0.7)	3.9	3.3
Bmp4	2.6	2.6	11.7	BT (0.7)	6.6	BT (1.0)	BT (0.3)	BT (1.0)	0.1
Bmp5	BT (0.4)	BT (1.6)	BT (1.8)	BT (1.3)	6.6	4.0	BT (1.1)	BT (1.6)	0.6
Bmp7	BT (1.7)	6.3	BT (1.5)	8.5	12.1	30.1	BT (1.2)	5.9	2.5
Btc	BT (0.4)	BT (0.0)	BT (1.1)	BT (0.0)	BT (0.2)	BT (1.6)	BT (1.5)	4.6	6.9
Ccl11	18.0	70.9	42.6	19.0	89.3	85.9	21.4	40.4	1.0
Ccl19	BT (0.1)	BT (0.0)	4.9	BT (0.0)	BT (0.0)	BT (0.0)	BT (0.5)	BT (0.0)	-
Ccl2	BT (0.7)	19.8	14.4	10.5	16.1	81.6	25.6	49.5	5.1
Ccl3	BT (0.1)	BT (0.0)	2.0	2.0	BT (0.0)	5.2	5.8	3.6	>5.2
Ccl5	BT (0.2)	BT (0.0)	2.2	BT (1.3)	BT (0.2)	3.0	2.5	3.3	12.7
Ccl7	6.0	29.1	12.7	10.5	36.0	77.3	23.8	44.6	2.1
Ccl9	BT (0.2)	2.9	5.2	2.6	16.8	72.1	12.0	26.1	4.3
Clcf1	BT (0.8)	BT (0.5)	BT (1.6)	BT (1.3)	BT (0.9)	4.4	5.0	5.2	4.6
Crlf1	BT (0.2)	BT (0.0)	BT (1.1)	5.2	BT (0.0)	26.1	3.4	11.1	>26.1
Csf1	12.2	3.4	40.9	11.8	10.9	31.0	24.0	30.9	2.8
Ctgf	7.3	BT (1.9)	18.9	19.0	2.4	37.2	29.2	25.4	15.7
Cx3cl1	BT (0.0)	BT (0.8)	BT (0.9)	BT (0.7)	BT (1.7)	14.3	8.6	4.2	8.6
Cxcl1	1.9	23.3	20.1	17.6	53.1	60.7	35.9	37.8	1.1
Cxcl10	BT (0.4)	BT (1.3)	BT (0.9)	BT (0.7)	BT (1.7)	9.6	3.5	6.5	5.8
Cxcl12	17.3	63.2	42.8	10.5	61.1	52.9	50.2	48.5	0.9
Cxcl13	BT (0.0)	BT (0.3)	17.6	BT (0.0)	BT (1.2)	BT (0.4)	BT (1.0)	BT (0.7)	0.3
Cxcl16	BT (0.8)	BT (1.3)	10.3	3.9	BT (1.7)	5.1	8.2	4.6	3.0
Cxcl2	BT (0.1)	BT (1.3)	7.1	6.5	BT (0.5)	24.7	12.8	20.2	52.2
Cxcl9	BT (1.0)	BT (1.9)	BT (1.4)	BT (0.0)	BT (0.5)	2.2	7.6	7.5	4.6
Dhh	BT (1.3)	2.4	BT (0.0)	BT (0.7)	BT (0.2)	BT (0.5)	BT (0.8)	2.3	2.3
Eda	8.2	10.6	4.0	19.6	7.8	9.4	7.7	7.5	1.2
Edn3	5.3	BT (0.5)	6.6	6.5	BT (1.7)	BT (0.3)	BT (1.6)	BT (1.0)	0.2
Efna1	4.1	3.7	3.2	5.9	4.0	7.8	3.9	4.2	1.9
Efna2	3.6	3.7	2.4	BT (1.3)	BT (1.2)	4.4	4.9	5.2	3.7
Efna4	4.2	BT (1.9)	2.5	6.5	BT (0.7)	6.1	9.0	3.9	8.6
Efna5	6.1	4.2	4.7	11.1	BT (1.2)	5.2	2.0	4.9	4.4
Efnb1	27.0	25.9	9.5	39.2	14.2	37.8	31.7	32.6	2.7
Efnb2	9.5	17.2	3.4	23.5	10.9	41.9	19.7	20.5	3.8
Fgf1	7.0	3.7	5.7	15.7	BT (0.9)	4.6	9.9	7.2	4.9
Fgf10	4.0	BT (0.0)	13.6	BT (0.0)	BT (0.0)	BT (0.7)	3.9	BT (1.6)	-
Fgf18	6.6	BT (0.0)	11.7	4.6	BT (1.9)	BT (1.5)	5.9	6.2	0.8
Fgf5	BT (1.9)	BT (0.0)	BT (0.5)	BT (0.0)	BT (0.2)	BT (0.1)	2.1	2.0	0.6
Fgf7	21.3	4.0	27.8	23.5	6.6	35.9	24.2	25.1	5.4
Figf	6.1	BT (0.8)	21.2	5.9	2.1	9.7	10.2	15.6	4.5
Fstl1	96.2	92.3	96.9	86.3	76.1	94.7	96.1	90.6	1.2
Gas6	26.6	BT (0.5)	51.4	88.2	9.7	11.1	38.7	20.5	1.1
Gdf10	5.0	BT (0.3)	42.0	3.3	4.3	4.5	17.8	12.1	1.1
Gdf11	4.0	7.1	BT (1.6)	3.3	4.3	9.8	5.3	4.6	2.3
Gmfb	13.7	18.3	15.2	28.1	9.2	34.4	25.9	30.6	3.7
Gnrh1	BT (0.8)	BT (0.8)	BT (1.9)	3.3	BT (0.5)	2.2	BT (1.5)	BT (1.6)	4.6
Hbegf	2.3	2.1	BT (1.4)	BT (1.3)	2.4	4.9	9.3	13.4	2.1
Hgf	BT (0.4)	BT (0.0)	BT (1.2)	BT (0.7)	BT (0.2)	BT (0.8)	6.0	6.2	3.5
Igf1	44.2	82.8	75.8	17.6	45.0	85.1	84.1	63.8	1.9
Igf2	91.9	92.3	4.7	20.9	8.8	9.0	26.1	25.1	1.0
Il15	BT (0.6)	BT (1.3)	BT (1.4)	9.8	4.7	9.3	4.2	2.6	2.0
Il16	BT (0.4)	BT (0.5)	2.2	BT (0.7)	BT (1.2)	3.1	BT (1.3)	BT (1.0)	2.7
Il18	BT (0.3)	BT (0.3)	9.4	BT (0.7)	4.0	5.5	5.2	5.2	1.4
Il1b	BT (0.0)	BT (0.0)	2.3	BT (0.7)	BT (0.0)	7.4	5.9	7.2	>7.4
Il33	17.2	13.8	32.7	56.2	72.7	63.7	40.8	44.6	0.9
Il6	BT (0.2)	2.1	3.7	BT (0.0)	2.8	8.1	10.0	8.8	2.8
Inha	2.0	BT (1.3)	BT (1.9)	2.0	BT (1.2)	2.3	2.3	2.3	2.0
Inhba	3.4	BT (0.5)	2.1	6.5	BT (1.4)	12.8	15.8	23.5	9.0
Inhbb	BT (0.0)	BT (0.0)	BT (1.3)	2.6	BT (0.0)	12.8	BT (1.6)	2.3	>12.8
Jag1	22.3	13.8	13.4	25.5	4.5	16.7	23.4	23.1	3.7
Lif	BT (1.3)	BT (1.1)	2.4	BT (0.0)	3.1	11.6	2.1	6.2	3.8
Mdk	58.8	70.9	16.1	30.1	16.4	54.6	65.9	45.6	3.3
Metrn	5.2	10.3	3.1	5.9	BT (1.2)	13.3	5.1	23.5	11.2
Mif	21.1	30.4	18.6	15.0	14.9	42.2	42.5	54.7	2.8
Nenf	54.7	62.7	55.7	49.0	49.8	59.7	60.5	59.6	1.2
Ngf	2.3	13.5	BT (1.2)	8.5	2.6	7.7	BT (1.7)	6.8	2.9
Nov	16.7	5.6	30.0	BT (0.7)	8.1	9.7	17.6	22.5	1.2
Nppc	2.1	7.7	BT (0.3)	BT (0.0)	2.6	6.3	8.3	4.2	2.4
Ntf3	BT (1.3)	BT (0.5)	4.6	BT (0.0)	BT (0.7)	BT (1.4)	5.3	3.6	1.9
Ntn1	21.7	BT (0.5)	24.8	61.4	12.8	25.0	11.1	18.6	2.0
Pdgfa	4.0	2.6	6.5	9.8	BT (1.7)	7.9	11.0	25.1	4.8
Pdgfc	BT (0.7)	BT (0.3)	6.0	BT (0.0)	BT (0.2)	BT (1.4)	7.2	8.1	5.8
Pf4	BT (0.8)	BT (1.1)	2.0	BT (1.3)	BT (0.0)	4.0	3.7	4.9	>4.0
Pgf	BT (1.4)	BT (1.1)	2.0	3.3	5.0	12.7	2.5	3.9	2.6
Pomc	BT (0.3)	BT (1.1)	BT (0.9)	BT (0.7)	BT (1.4)	3.1	BT (1.2)	BT (1.3)	2.2
Pthlh	5.7	6.3	9.8	20.3	7.6	13.8	13.4	11.1	1.8
Ptn	21.1	21.7	15.5	2.6	4.0	11.1	56.8	45.6	2.7
Rspo1	11.5	BT (0.0)	3.2	20.9	BT (1.2)	BT (0.5)	BT (0.5)	BT (0.3)	0.5
Rspo3	BT (0.4)	BT (0.0)	3.1	BT (1.3)	BT (0.0)	BT (0.7)	11.6	6.2	-
Rtn4	35.1	36.2	47.9	43.8	33.9	68.6	60.5	72.3	2.0
Sema3b	5.2	9.3	14.0	11.1	33.4	11.3	5.0	6.5	0.3
Sema3c	43.0	14.8	50.5	18.3	9.2	8.6	23.0	27.7	0.9
Sema3d	18.0	3.7	11.4	45.1	BT (0.7)	BT (1.8)	6.3	7.8	2.5
Sema3e	BT (0.7)	BT (0.3)	3.7	BT (0.0)	BT (0.2)	BT (0.1)	BT (1.0)	2.9	0.6
Sema3f	2.2	BT (0.5)	BT (1.1)	2.6	BT (0.7)	4.5	2.0	3.3	6.3
Sema3g	BT (0.3)	BT (1.3)	BT (0.6)	BT (0.0)	3.1	2.7	BT (1.0)	2.0	0.9
Sema4a	BT (0.8)	BT (1.9)	BT (0.6)	4.6	BT (1.7)	3.3	BT (1.3)	BT (1.6)	2.0
Sema4b	BT (1.7)	BT (0.3)	BT (1.5)	2.0	2.1	2.9	2.6	2.3	1.3
Sema4c	5.8	5.8	5.9	13.1	6.4	11.7	9.3	10.1	1.8
Sema5a	4.7	7.4	BT (1.8)	4.6	3.6	3.6	6.1	5.9	1.0
Sema5b	5.6	9.5	BT (0.2)	BT (0.0)	BT (1.2)	BT (1.1)	BT (1.6)	5.2	0.9
Sema6a	6.2	9.8	3.1	7.8	19.0	14.6	3.2	7.5	0.8
Sema6b	BT (0.1)	BT (0.3)	2.5	BT (0.0)	2.8	7.1	BT (1.3)	7.8	2.5
Sema6c	4.3	4.8	5.4	BT (1.3)	3.3	3.4	8.0	6.2	1.0
Sema6d	2.2	3.2	3.7	7.8	3.6	11.2	3.7	9.4	3.2
Sema7a	BT (0.3)	BT (0.5)	BT (1.3)	BT (0.7)	BT (0.9)	19.4	11.0	21.5	20.5
Sfrp1	60.0	29.1	40.0	54.2	15.2	32.9	50.0	32.6	2.2
Sfrp2	11.4	BT (0.3)	47.8	3.9	5.5	7.1	21.7	18.6	1.3
Sfrp4	33.6	BT (1.9)	72.4	32.7	9.0	50.7	56.8	39.1	5.6
Sfrp5	26.7	2.6	2.6	94.8	5.9	2.6	6.3	10.4	0.4
Tgfb1	BT (0.6)	BT (0.3)	2.4	3.3	3.3	11.1	10.0	15.3	3.3
Tgfb2	10.3	BT (1.1)	10.5	3.3	2.8	3.7	17.0	12.1	1.3
Tgfb3	20.6	3.4	16.8	19.0	2.4	9.0	36.1	19.2	3.8
Tnf	BT (0.0)	BT (0.3)	BT (1.7)	BT (0.7)	BT (0.0)	2.5	BT (0.7)	2.6	>2.5
Tnfrsf11b	BT (1.9)	BT (0.0)	4.6	8.5	BT (0.5)	BT (0.7)	4.4	10.7	1.4
Tnfsf10	BT (0.0)	BT (0.0)	BT (0.4)	2.0	BT (1.7)	BT (0.7)	BT (0.2)	BT (1.0)	0.4
Tnfsf12	9.6	11.6	15.7	15.0	17.8	17.3	14.6	14.7	1.0
Tnfsf8	BT (0.3)	BT (0.0)	BT (0.8)	10.5	BT (0.0)	2.2	5.7	3.3	>2.2
Tnfsf9	BT (0.5)	BT (1.6)	BT (1.4)	BT (1.3)	4.0	5.9	4.5	7.2	1.5
Tslp	BT (1.2)	2.4	4.0	2.6	BT (0.2)	4.8	BT (1.2)	2.9	20.2
Vegfa	11.0	4.8	25.0	3.9	8.3	18.2	29.6	27.0	2.2
Vegfb	5.2	9.0	5.4	9.8	4.7	7.1	8.3	11.4	1.5
Vegfc	BT (0.7)	BT (0.0)	2.2	BT (0.7)	BT (0.2)	BT (0.7)	2.2	5.5	2.9
Wnt11	BT (1.2)	BT (0.0)	6.2	BT (0.0)	BT (0.5)	BT (0.5)	BT (0.9)	2.9	1.2
Wnt2	6.0	BT (0.3)	9.1	BT (0.0)	BT (0.2)	BT (1.5)	4.6	4.9	6.3
Wnt5a	15.5	4.0	19.1	7.8	10.4	13.9	13.0	14.0	1.3

The combined mesenchymal cell dataset (Fig. 4*A,B*) was analyzed to determine the percentage of cells within the different mesenchymal cell clusters that detectably expressed (≥2%) the 143 injured nerve ligand mRNAs (Table 2). Also shown is the difference, expressed as fold change, in the percentage of positive cells in the injured versus uninjured endoneurial mesenchymal cells. BT = below threshold and indicates that <2% of cells detectably expressed the ligand mRNA. Also shown are the absolute values, since these were used to determine the fold changes. Neonatal epineurial includes cluster 1 cells, neonatal endoneurial includes cluster 6 cells, inj/uninjured epineurial includes clusters 2 and 5 cells, neonatal/uninjured/injured perineurial includes cluster 8 cells, uninjured endoneurial includes cluster 4 cells, injured endoneurial includes cluster 3 cells, injured differentiating includes cluster 0 cells, and neonatal/injured proliferating includes cluster 7 cells.

### Identification of growth factor receptors on peripheral sympathetic and sensory neurons

To determine which of these ligands are likely to be important for axonal growth, we characterized growth factor receptors on sensory and sympathetic neurons which project their axons via the sciatic nerve. To do this, we coupled cell-surface proteomics and transcriptome profiling on purified neuronal cultures. For sensory neurons, we cultured E15 rat DRG neurons for 9 d in medium containing NGF. Immunostaining showed that these cultures were comprised of relatively pure βIII-tubulin-positive neurons with 2–3% contaminating S100β-positive Schwann cells (Fig. 5*A*; Extended Data Fig. 5-1*A*). For sympathetic neurons, we isolated cells from the neonatal rat SCG and cultured them for 6 d in NGF. These cultures contained βIII-tubulin-positive neurons and low percentages of Fibronectin-positive fibroblasts and S100β-positive Schwann cells (Fig. 5*A*; Extended Data Fig. 5-1*A*).

Initially, we characterized the neuronal cell-surface proteomes, taking advantage of the fact that many cell-surface proteins are glycosylated. Specifically, we performed periodate oxidation of cell-surface glycans, bound modified proteins on a hydrazide resin after cell lysis, digested the bound proteins with trypsin and PNGase F, and identified peptides by mass spectrometry. For each sample, we analyzed three independent biological replicates. This analysis identified 608 and 271 unique proteins on sensory and sympathetic neurons, respectively, with 219 of these common to both populations (Extended Data Fig. 5-1*B*; Table 7). The lower number of unique proteins on sympathetic neurons is likely due to decreased protein that was isolated (samples averaged ≈1100 vs 300 μg/ml for sensory vs sympathetic neurons). PANTHER classification identified most of these proteins as receptors, transporters and hydrolases, indicating appropriate enrichment for cell-surface proteins (Extended Data Fig. 5-1*C*). We then used the ligand-receptor database and manual curation to identify 102 proteins as receptors of various types, including G-protein-coupled receptors, receptor tyrosine kinases and phosphatases, cytokine receptors, and ligand-gated ion channels (Fig. 5*B*; Table 8). Among these were well-characterized receptors found on both types of neurons such as TrkA (encoded by *Ntrk1*), BMP receptor 2, RET, gp130 (encoded by *Il6st*), and IGF2 receptor, receptors identified only on sensory neurons such as GFRα3 and receptors identified only on sympathetic neurons such as ALK and GFRα2.

**Table 7:** Proteins identified in sensory (DRG) and sympathetic (SCG) neurons using mass spectrometry

Sensory neurons(608)		Sympatheticneurons (271)		Sensory and sympatheticintersect (219)
Abca1		11/3R		Abca1
Abca5		Abca1		Ache
Abca7		AceIII		Acp2
Ache		Ache		Adam22
Acp2		Acp2		Adam23
Actb		Adam22		Adgre5
Actg1		Adam23		Adgrl1
Acvr2a		Adgre1		Adgrl2
Adam10		Adgre5		Ahsg
Adam11		Adgrl1		Alcam
Adam19		Adgrl2		Ano6
Adam22		Ahsg		Aplp1
Adam23		Alcam		Apmap
Adam9		Alk		Asah1
Adcy6		Angpt2		Atp1b1
Adcy9		Ano6		Atp6ap1
Adgrb3		Anpep		B3glct
Adgre5		Aplp1		Bcam
Adgrl1		Apmap		Bgn
Adgrl2		Asah1		Bmpr2
Adgrl3		Aspm		Bsg
Agrn		Atp1b1		Bst2
Ahsg		Atp6ap1		Cacna2d1
Alcam		B3glct		Cadm1
Alpl		Bcam		Cadm2
Alpl1		Bgn		Cadm4
Ano6		Bmpr2		CatL
Aplp1		Bsg		Cd151
Aplp2		Bst2		Cd200
Apmap		Cacna2d1		Cd276
Arse		Cadm1		Cd320
Asah1		Cadm2		Cd47
Asph		Cadm4		Cd59
Astn2		CatL		Cd63
Atg9a		Cd151		Cdh2
Atp1a1		Cd200		Celsr3
Atp1b1		Cd276		Chl1
Atp1b3		Cd320		Clmp
Atp5a1		Cd47		Clu
Atp6ap1		Cd59		Cntn1
Atraid		Cd63		Cntn2
Atrn		Cdh17		Cntnap1
Atrnl1		Cdh2		Col1a1
Avil		Cdig2		Col5a2
B3gat3		Cdk5r2		Colgalt1
B3glct		Celsr3		Cpd
Bace1		Chl1		Cpe
Bcam		Clmp		Ctsa
Bgn		Clu		Ctsc
Bmper		Cnnm2		Ctsd
Bmpr2		Cntn1		Ctsl
Brinp1		Cntn2		Ctsz
Brinp2		Cntnap1		Dpp10
Bscl2		Col1a1		Dpp6
Bsg		Col2a1		Ece1
Bst2		Col5a2		Efna5
Btd		Colgalt1		Efnb2
C11orf24		Cp		Emb
Cacna1b		Cpd		Enpp4
Cacna1c		Cpe		Entpd2
Cacna2d1		Cst3		Ephb2
Cacna2d2		Ctsa		Ero1a
Cacng8		Ctsc		Fam234b
Cadm1		Ctsd		Fn1
Cadm2		Ctsl		Gaa
Cadm3		Ctsz		Gabbr1
Cadm4		Cyp4f17		Gba
Calm1		Cyp4f40		Gdpd5
Calm2		Dbh		Ggt7
Calu		Dio1		Glg1
Cant1		Dkk3		Gns
Car11		Dopey1		Gpc1
Casc4		Dpp10		Grik3
Casd1		Dpp6		Grm7
CatL		Ece1		Hexa
Cd151		Ecel1		Hs2st1
Cd164		Efna5		Hsp90b1
Cd200		Efnb2		Hyou1
Cd24		Emb		Icam1
Cd276		Enpp4		Igf2r
Cd320		Entpd2		Iglon5
Cd44		Ephb2		Igsf3
Cd47		Ero1a		Il6st
Cd55		F2r		Impad1
Cd59		Fam234b		Insr
Cd63		Fcrl2		Islr2
Cd81		Fn1		Itga1
Cdh18		Folr2		Itga3
Cdh2		Gaa		Itga5
Cdh4		Gabbr1		Itga6
Celsr2		Gba		Itgam
Celsr3		Gdpd5		Itgav
Cemip		Gfra2		Itgb1
Chl1		Ggt7		L1cam
Chpf2		Glg1		Lamb1
Chst3		Gnas		Lamc1
Clcn5		Gns		Lamp1
Clcn6		Gpc1		Ldlr
Clmp		Grik3		Lgals3bp
Clptm1		Grm7		Lnpep
Clu		H2-Q10		LOC100912445
Cntfr		H2-Q7		LOC679087
Cntn1		Hexa		Lrp1
Cntn2		Hs2st1		Lrp11
Cntn3		Hsp90b1		Lrrc8b
Cntn4		Hyou1		Lsamp
Cntn6		Icam1		Ly6h
Cntnap1		Igf2r		Man2a2
Cntnap4		Iglon5		Mcam
Col12a1		Igsf3		Mcoln1
Col18a1		Il6st		Mdga1
Col1a1		Impad1		Megf8
Col5a1		Insr		Megf9
Col5a2		Islr2		Mmp15
Colgalt1		Itga1		Ncam1
Colgalt2		Itga3		Ncam2
Cpd		Itga5		Ncstn
Cpe		Itga6		Negr1
Cpm		Itga8		Nell1
Cr1l		Itgam		Neo1
Creld1		Itgav		Nfasc
Crtac1		Itgb1		Npc1
Csmd1		L1cam		Nptn
Csmd2		Lama1		Nrcam
Cspg5		Lamb1		Nrp1
Ctsa		Lamc1		Nrxn1
Ctsc		Lamp1		Nrxn3
Ctsd		Ldlr		Ntrk1
Ctsl		Lgals3bp		Olfm1
Ctsz		Lnpep		Ostm1
Cxadr		LOC100912445		P2rx4
Daf1		LOC286987		P4htm
Dchs1		LOC679087		Panx1
Dgcr2		Lrp1		Pcdh1
Disp2		Lrp11		Pcdh17
Dnase2		Lrrc8b		Pcdh9
Dpp10		Lsamp		Pcdhgc3
Dpp6		Ly6h		Pcyox1
Dpp7		Man2a2		pE4_antigen
Dpysl2		Mcam		Plbd2
Dpysl3		Mcoln1		Pld3
Ece1		Mdga1		Plod1
Ece2		Megf8		Plod3
Edem3		Megf9		Plxna1
Edil3		Mlnr		Plxna3
Eef1a1		Mmp15		Plxna4
Efna1		Mrc1		Plxnb1
Efna3		Mtor		Plxnb2
Efna5		Ncam1		Plxnc1
Efnb1		Ncam2		Ppt1
Efnb2		Ncstn		Prnp
Efnb3		Negr1		Ptk7
Elfn1		Nell1		Ptprg
Elfn2		Neo1		Pttg1ip
Emb		Nfasc		PVR
Enpp4		Nkain3		Pvrl1
Enpp5		Npc1		Pvrl2
Entpd2		Nptn		Rbm12b
Epdr1		Nrcam		Ret
Epha2		Nrp1		rt1-E
Ephb2		Nrxn1		Scarb2
Ero1a		Nrxn3		Scn2b
Extl3		Nt5e		Scn3a
F11r		Ntng1		Scn9a
F2rl2		Ntrk1		Sdk2
Fam189b		Olfm1		Sel1l
Fam234b		Ostm1		Sema4c
Fat1		P2rx4		Sema4d
Fat3		P4htm		Sez6l2
Fat4		Panx1		Sgce
Fbn2		Pcdh1		Slc12a7
Fkbp10		Pcdh17		Slc2a1
Fkbp9		Pcdh9		Slc2a13
Flrt1		Pcdhgc3		Slc2a3
Fn1		Pcyox1		Slc39a6
Foxred2		pE4_antigen		Slc44a2
Fras1		Plbd2		Slc6a15
Fstl1		Pld3		Slco3a1
Gaa		Plod1		Slit1
Gabbr1		Plod3		Slit2
Gabbr2		Plxna1		Sorcs2
Gabra2		Plxna3		Sort1
Gabrb3		Plxna4		Spock2
Galnt9		Plxnb1		Ssr2
Gapdh		Plxnb2		Stt3a
Gapdh-ps2		Plxnc1		Stt3b
Gba		Pon1		Suco
Gdpd5		Ppt1		Sulf2
Gfra3		Prnp		Sv2a
Ggh		Ptk7		Sv2b
Ggt5		Ptprg		Sv2c
Ggt7		Ptprm		Tage4
Gla		Pttg1ip		Tenm3
Glg1		PVR		Tenm4
Gnao1		Pvrl1		Tfrc
Gnptab		Pvrl2		Thbs1
Gns		Rbm12b		Thsd7a
Gpc1		Ret		Thy1
Gpm6b		RGD1560108		Timp1
Gpr158		RT1-A2b		Tm9sf3
Gria2		RT1-Ak		Tmed4
Grik3		RT1-Aw2		Tmed7
Grin1		rt1-E		Tmed9
Grm7		RT1.A1		Tmeff1
Grn		Rt1.L		Tmem106b
Hexa		Scarb2		Tmem132a
Hist1h2ba		Scn2b		Tmem63b
Hist1h2bd		Scn3a		Tmem63c
Hist1h2bh		Scn9a		Tmem87a
Hist1h2bk		Scube1		Tmem87b
Hist1h2bl		Sdk2		Tpp1
Hist1h2bo		Sel1l		Trpv2
Hist1h2bq		Sema4c		Tspan3
Hist2h2be		Sema4d		Tspan6
Hist3h2ba		Sez6l2		Tspan8
Hist3h2bb		Sgce		Ttyh3
Hnrnpa1		Slc12a7		Unc5b
Hs2st1		Slc2a1		Unc5c
Hs6st1		Slc2a13		Vwa7
Hsp70		Slc2a3		
Hsp90ab1		Slc39a6		
Hsp90b1		Slc44a2		
Hspa13		Slc6a15		
Hspa2		Slc6a2		
Hspa8		Slco3a1		
Hyou1		Slit1		
Icam1		Slit2		
Icam5		Sorcs1		
Ids		Sorcs2		
Idua		Sorcs3		
Ifnar1		Sort1		
Igf1r		Spock2		
Igf2r		Ssr2		
Igfbpl1		Stab1		
Iglon5		Stt3a		
Igsf3		Stt3b		
Ikbip		Suco		
Il1rapl1		Sulf2		
Il6st		Sv2a		
Impad1		Sv2b		
Insr		Sv2c		
Islr2		Tage4		
Itfg1		Tenm3		
Itga1		Tenm4		
Itga3		Tfrc		
Itga4		Thbs1		
Itga5		Thsd7a		
Itga6		Thy1		
Itga7		Timp1		
Itga9		Tll2		
Itgal		Tm9sf3		
Itgam		Tmed4		
Itgav		Tmed7		
Itgb1		Tmed9		
Itgb8		Tmeff1		
Jag1		Tmem106b		
Jkamp		Tmem132a		
Kcnc4		Tmem63b		
Kdelc2		Tmem63c		
Kiaa0319		Tmem87a		
Kirrel3		Tmem87b		
L1cam		Tpp1		
Lama4		Trpv2		
Lama5		TSLC1		
Lamb1		Tspan3		
Lamc1		Tspan6		
Lamp1		Tspan8		
Lamp2		Ttyh3		
Ldlr		Unc5b		
Lgals3bp		Unc5c		
Lgi2		Vwa7		
Lifr				
Lingo1				
Lingo2				
Lman2l				
Lnpep				
LOC100294508				
LOC100359563				
LOC100360413				
LOC100360548				
LOC100364116				
LOC100909441				
LOC100909911				
LOC100911252				
LOC100912445				
LOC100912446				
LOC102549061				
LOC102549957				
LOC314016				
LOC679087				
LOC685186				
Lphn3				
Lppr1				
Lrfn1				
Lrfn4				
Lrfn5				
Lrig2				
Lrp1				
Lrp11				
Lrp3				
Lrp8				
Lrrc8a				
Lrrc8b				
Lrrn1				
Lsamp				
Ly6h				
M6pr				
Man2a2				
Man2b1				
Mcam				
Mcoln1				
Mdga1				
Mdga2				
Megf8				
Sensoryneurons(608)				
Megf9				
Mfge8				
Mme				
Mmp15				
Mpz				
Mpzl1				
Mrc2				
Mst1r				
Myh7b				
Naglu				
Ncam1				
Ncam2				
Ncan				
Ncln				
Ncstn				
Negr1				
Nell1				
Neo1				
Neu1				
Nfasc				
Nid2				
NKCC1				
Nomo1				
Npc1				
Npr3				
Nptn				
Nptx1				
Nptx2				
Nptxr				
Nrcam				
Nrp1				
Nrxn1				
Nrxn2				
Nrxn3				
Ntm				
Ntng2				
Ntrk1				
Ntrk2				
Olfm1				
Ostm1				
P2rx3				
P2rx4				
P3h1				
P3h4				
P4ha1				
P4htm				
Panx1				
Pcdh1				
Pcdh17				
Pcdh19				
Pcdh7				
Pcdh9				
Pcdha6				
Pcdha8				
Pcdhb2				
Pcdhb3				
Pcdhga11				
Pcdhga3				
Pcdhga5				
Pcdhga7				
Pcdhgb4				
Pcdhgb5				
Pcdhgb6				
Pcdhgb7				
Pcdhgb8				
Pcdhgc3				
Pcsk2				
Pcyox1				
pE4_antigen				
Pgap1				
Piezo2				
Pigs				
Pigt				
Pla2g15				
Plaur				
Plbd2				
Pld3				
Plod1				
Plod2				
Plod3				
Plxdc2				
Plxna1				
Plxna2				
Plxna3				
Plxna4				
Plxnb1				
Plxnb2				
Plxnc1				
Plxnd1				
Pm20d1				
Podxl				
Podxl2				
Pofut2				
Pol				
Pomgnt2				
Postn				
Ppia				
Ppib				
Ppil1				
Ppt1				
Prdx2				
Prnp				
Prph				
Psap				
Ptger2				
SensoryNeurons(608)				
Ptgfrn				
Ptk7				
Ptprf				
Ptprg				
Ptprn				
Ptpro2				
Ptprs				
Pttg1ip				
PVR				
Pvrl1				
Pvrl2				
Pxdn				
Qpct				
ratASCT1				
Rbm12b				
Rcn1				
Ret				
RGD1562725				
RGD1563124				
RGD1563349				
RGD1565368				
Rgma				
Ror2				
Rps16				
Rps20				
Rps27a				
Rps27a-ps6				
rt1-E				
Rtn4r				
Rtn4rl1				
Scarb2				
Scg3				
Scn10a				
Scn2b				
Scn3a				
Scn7a				
Scn9a				
Sdk2				
Sel1l				
Sema3c				
Sema3f				
Sema3g				
Sema4b				
Sema4c				
Sema4d				
Sema4f				
Sema6a				
Sema6d				
Sema7a				
Serpinh1				
Sez6l2				
Sgcb				
Sgce				
Shisa7				
Siae				
Sil1				
Sirpa				
Slc12a2				
Slc12a4				
Slc12a7				
Slc12a9				
Slc1a2				
Slc1a4				
Slc22a23				
Slc24a2				
Slc24a3				
Slc25a31				
Slc25a4				
Slc25a5				
Slc2a1				
Slc2a13				
Slc2a3				
Slc35a5				
Slc38a2				
Slc39a10				
Slc39a14				
Slc39a6				
Slc39a8				
Slc3a2				
Slc44a1				
Slc44a2				
Slc46a1				
Slc4a1				
Slc52a2				
Slc6a15				
Slc6a17				
Slc6a8				
Slc7a1				
Slc8a1				
Slco3a1				
Slit1				
Slit2				
Slitrk2				
Slitrk3				
Sorcs2				
Sort1				
Spock2				
Spock3				
Sppl2a				
Sppl2b				
Ssr2				
St8sia1				
St8sia3				
Stt3a				
Stt3b				
Sensoryneurons(608)				
Suco				
Sulf2				
Sun1				
Sun2				
Sv2a				
Sv2b				
Sv2c				
Syp				
Sypl1				
Tage4				
Tapbp				
Tctn1				
Tctn2				
Tenm2				
Tenm3				
Tenm4				
Tfrc				
Tgfb2				
Tgfbr3				
Thbs1				
Thsd7a				
Thsd7b				
Thy1				
Timp1				
Tm2d1				
Tm9sf3				
Tmed4				
Tmed7				
Tmed9				
Tmeff1				
Tmem106b				
Tmem132a				
Tmem132c				
Tmem132e				
Tmem158				
Tmem181				
Tmem2				
Tmem200c				
Tmem231				
Tmem255a				
Tmem63b				
Tmem63c				
Tmem87a				
Tmem87b				
Tmem9b				
Tmtc4				
Tmx3				
Tor1aip2				
Tor2a				
Tpbg				
Tpcn1				
Tpp1				
Trhde				
Trpv2				
Tspan13				
Tspan3				
Tspan6				
Tspan7				
Tspan8				
Ttyh3				
Tuba1a				
Tuba1b				
Tuba1c				
Tuba3a				
Tubb2a				
Tubb2b				
Tubb3				
Tubb4b				
Tubb5				
Txndc15				
Uba52				
Ubb				
Ubc				
Uggt1				
Unc5b				
Unc5c				
Ust				
Vstm2a				
Vstm5				
Vwa7				
Wbscr17				
Ywhag				
Ywhah				
Z043_117466				

Gene symbols of proteins identified using cell-surface capture mass spectrometry on sensory neurons (column 1), sympathetic neurons (column 2), and both neuron types (column 3; intersect). Total numbers of proteins are indicated. Proteins included in this list were annotated by the terms “cell membrane” and/or “secreted” by the UniProtKB database (http://uniprot.org) and were verified by manual curation.

**Table 8:** Receptors identified on sensory (DRG) and sympathetic (SCG) neurons using mass spectrometry and microarrays

Mass spectrometry		
DRGs (42)	SCGs (13)	DRGs and SCGs (47)
Acvr2a	Adgre1	Adgre5
Adgrb3	Alk	Adgrl1
Adgrl3	F2r	Adgrl2
Cd44	Fcrl2	Bcam
Celsr2	Folr2	Bmpr2
Cntfr	Gfra2	Cd320
Epha3	Itga8	Cd63
F2rl2	Mrc1	Celsr3
Gabra2	Mlnr	Ephb2
Gabrb3	Ntng1	Gabbr1
Gfra3	Ptprm	Grik3
Gpr158	Sorcs1	Grm7
Gria2	Sorcs3	Icam1
Grin1		Igf2r
Ifnar1		Il6st
Igf1r		Insr
Itfg1		Itga1
Itga4		Itga3
Itga7		Itga5
Itga9		Itga6
Itgal		Itgam
Itgb8		Itgav
Lifr		Itgb1
Lingo1		Ldlr
Mrc2		Lrp1
Mst1r		Mcam
Npr3		Neo1
Ntng2		Nptn
Ntrk2		Nrp1
P2rx3		Ntrk1
Plaur		P2rx4
Plxna2		Plxna1
Plxnd1		Plxna3
Ptger2		Plxna4
Ptprf		Plxnb1
Ptprn		Plxnb2
Ptprs		Plxnc1
Ror2		Ptprg
Rtn4r		PVR
Rtn4rl1		Pvrl1
Sirpa		Pvrl2
Tgfbr3		Ret
		Sorcs2
		Sort1
		Spock2
		Unc5b
		Unc5c
Microarrays		
DRGs (321)	SCGs (297)	
Acvr1	Acvr1	
Acvr1b	Acvr1b	
Acvr1c	Acvr2a	
Acvr2a	Acvr2b	
Acvr2b	Acvrl1	
Acvrl1	Adcyap1r1	
Adcyap1r1	Adipor1	
Adipor1	Adipor2	
Adipor2	Adora2a	
Adora2a	Adra1b	
Microarrays		
DRGs (321)	SCGs (297)	
Adra1a	Adrb2	
Adra1b	Ager	
Adrb2	Alk	
Ager	Amfr	
Alk	Aplnr	
Amfr	Avpr1a	
Amhr2	Avpr2	
Aplnr	Axl	
Ar	Bdkrb2	
Avpr1a	Bmpr1a	
Avpr2	Bmpr1b	
Axl	Bmpr2	
Bdkrb2	C3ar1	
Bmpr1a	C5ar1	
Bmpr1b	Calcrl	
Bmpr2	Cckar	
Btn1a1	Cckbr	
C3ar1	Ccr10	
C5ar1	Ccr4	
Calcr	Ccr7	
Calcrl	Ccr8	
Cckar	Cd14	
Cckbr	Cd27	
Ccr10	Cd33	
Ccr4	Cd4	
Ccr7	Cd44	
Ccr8	Cd5l	
Cd14	Cd7	
Cd27	Cd74	
Cd33	Cntfr	
Cd4	Crhr1	
Cd40	Crhr2	
Cd44	Crlf1	
Cd5l	Crlf2	
Cd7	Csf1r	
Cd74	Csf2ra	
Cntfr	Csf2rb	
Crhr1	Csf3r	
Crhr2	Ctf1	
Crlf1	Cx3cr1	
Crlf2	Cxcr1	
Csf1r	Cxcr3	
Csf2ra	Cxcr4	
Csf2rb	Dcc	
Csf3r	Ddr1	
Ctf1	Derl1	
Cx3cr1	Dip2a	
Cxcr1	Edar	
Cxcr2	Ednra	
Cxcr3	Ednrb	
Cxcr4	Egfr	
Cxcr5	Eng	
Dcc	Epha1	
Ddr1	Epha2	
Derl1	Epha3	
Dip2a	Epha4	
Edar	Epha5	
Ednra	Epha7	
Ednrb	Ephb1	
Egfr	Ephb2	
Eng	Ephb3	
Microarrays		
DRGs (321)	SCGs (297)	
Epha1	Ephb4	
Epha2	Epor	
Epha3	Eps15l1	
Epha4	Erbb2	
Epha5	Erbb3	
Epha7	Esr2	
Ephb1	F2r	
Ephb2	F2rl1	
Ephb3	F2rl2	
Ephb4	F2rl3	
Epor	Fas	
Eps15l1	Fgfr1	
Erbb2	Fgfr2	
Erbb3	Fgfr3	
Erbb4	Fgfr4	
Esr2	Fgfrl1	
F2r	Flt1	
F2rl1	Flt3	
F2rl2	Flt4	
F2rl3	Folr1	
Fas	Fshr	
Fgfr1	Fzd1	
Fgfr2	Fzd2	
Fgfr3	Fzd4	
Fgfr4	Fzd5	
Fgfrl1	Fzd9	
Flt1	Gabbr1	
Flt3	Galr1	
Flt4	Galr2	
Folr1	Gcgr	
Fshr	Gfra2	
Fzd1	Gfra3	
Fzd2	Gfra4	
Fzd4	Ghr	
Fzd5	Ghrhr	
Fzd9	Ghsr	
Gabbr1	Gipr	
Galr1	Glp1r	
Galr2	Gosr1	
Gcgr	Grik5	
Gfra2	Grin2a	
Gfra3	Grin2b	
Gfra4	Grin2c	
Ghr	Grin2d	
Ghrhr	Gucy2c	
Ghsr	Hcrtr1	
Gipr	Hcrtr2	
Glp1r	Hnf4a	
Glp2r	Hpn	
Gnrhr	Ifnar1	
Gosr1	Ifnar2	
Grik5	Ifngr1	
Grin2a	Ifngr2	
Grin2b	Igf1r	
Grin2c	Igf2r	
Grin2d	Igfbp1	
Gucy2c	Igfbp2	
Hcrtr1	Igfbp3	
Hcrtr2	Igfbp4	
Hnf4a	Igfbp5	
Hpn	Igfbp6	
Microarrays		
DRGs (321)	SCGs (297)	
Ifnar1	Igfbp7	
Ifnar2	Il10ra	
Ifngr1	Il10rb	
Ifngr2	Il12rb1	
Igf1r	Il15ra	
Igf2r	Il17ra	
Igfbp1	Il17rc	
Igfbp2	Il18r1	
Igfbp3	Il18rap	
Igfbp4	Il1r1	
Igfbp5	Il1r2	
Igfbp6	Il1rap	
Igfbp7	Il1rl1	
Il10ra	Il1rl2	
Il10rb	Il20rb	
Il12rb1	Il21r	
Il12rb2	Il22ra1	
Il13ra2	Il22ra2	
Il15ra	Il27ra	
Il17ra	Il2ra	
Il17rb	Il2rb	
Il17rc	Il2rg	
Il18r1	Il3ra	
Il18rap	Il4r	
Il1r1	Il6r	
Il1r2	Il6st	
Il1rap	Il7r	
Il1rl1	Insr	
Il1rl2	Irs1	
Il20rb	Itga2	
Il21r	Itga2b	
Il22ra1	Itga5	
Il22ra2	Itga9	
Il27ra	Itgal	
Il2ra	Itgav	
Il2rb	Itgb1	
Il2rg	Itgb2	
Il3ra	Itgb3	
Il4r	Itgb5	
Il6r	Itgb6	
Il6st	Itgb8	
Il7r	Itpr3	
Il9r	Kit	
Insr	Ldlr	
Irs1	Lgals3bp	
Itga2	Lgr5	
Itga2b	Lifr	
Itga5	Lingo1	
Itga9	Loxl2	
Itgal	Lrp1	
Itgav	Lrp2	
Itgb1	Lrp5	
Itgb2	Lrp6	
Itgb3	Lsr	
Itgb5	Ltbr	
Itgb6	Marco	
Itgb8	Mc1r	
Itpr3	Mc2r	
Kdr	Mc3r	
Kit	Mc4r	
Ldlr	Mc5r	
Microarrays		
DRGs (321)	SCGs (297)	
Lgals3bp	Mchr1	
Lgr5	Met	
Lifr	Mpl	
Lingo1	Mst1r	
Loxl2	Ncoa3	
Lrp1	Ncor1	
Lrp2	Neo1	
Lrp5	Ngfr	
Lrp6	Notch1	
Lsr	Notch2	
Ltbr	Notch3	
Marco	Npffr1	
Mc1r	Npffr2	
Mc2r	Npr1	
Mc3r	Npr2	
Mc4r	Npr3	
Mc5r	Npy1r	
Mchr1	Npy2r	
Met	Npy5r	
Mpl	Nr3c1	
Mrgprx2	Nrp1	
Mst1r	Nrp2	
Mtnr1b	Ntng1	
Ncoa3	Ntng2	
Ncor1	Ntrk1	
Neo1	Ntrk2	
Ngfr	Ntrk3	
Notch1	Ntsr1	
Notch2	Oprl1	
Notch3	Osmr	
Npffr1	Oxtr	
Npffr2	Pdgfa	
Npr1	Pdgfra	
Npr2	Pdgfrb	
Npr3	Pgr	
Npy1r	Plaur	
Npy2r	Plgrkt	
Npy5r	Plxna1	
Nr3c1	Plxna2	
Nrp1	Plxna3	
Nrp2	Plxna4	
Ntng1	Plxnb1	
Ntng2	Plxnc1	
Ntrk1	Plxnd1	
Ntrk2	Procr	
Ntrk3	Prokr1	
Ntsr1	Prokr2	
Oprl1	Ptch1	
Osmr	Ptch2	
Oxtr	Pth1r	
Pdgfa	Pth2r	
Pdgfra	Ptprk	
Pdgfrb	Ptprs	
Pgr	Ptprz1	
Plaur	Ret	
Plgrkt	Robo3	
Plxna1	Ror1	
Plxna2	Ror2	
Plxna3	Rorb	
Plxna4	Rtn4r	
Plxnb1	Rtn4rl1	
Microarrays		
DRGs (321)	SCGs (297)	
Plxnc1	Rxrg	
Plxnd1	Ryr1	
Prlhr	Ryr2	
Prlr	Sctr	
Procr	Sdc4	
Prokr1	Sfrp1	
Prokr2	Sfrp2	
Ptch1	Slc1a5	
Ptch2	Sorcs3	
Pth1r	Sort1	
Pth2r	Sstr1	
Ptprh	Sstr2	
Ptprk	Sstr3	
Ptprs	Sstr4	
Ptprz1	Sstr5	
Ret	Tek	
Robo3	Tgfbr2	
Ror1	Tgfbr3	
Ror2	Thbd	
Rorb	Thra	
Rtn4r	Thrap3	
Rtn4rl1	Tnfrsf10b	
Rxfp1	Tnfrsf11a	
Rxrg	Tnfrsf11b	
Ryr1	Tnfrsf12a	
Ryr2	Tnfrsf13c	
Sctr	Tnfrsf14	
Sdc4	Tnfrsf17	
Sfrp1	Tnfrsf18	
Sfrp2	Tnfrsf1a	
Slc1a5	Tnfrsf1b	
Sorcs3	Tnfrsf25	
Sort1	Tnfrsf4	
Sstr1	Tnfrsf8	
Sstr2	Tnfrsf9	
Sstr3	Tshr	
Sstr4	Unc5b	
Sstr5	Unc5c	
Tek	Uts2r	
Tgfbr2	Vipr1	
Tgfbr3	Vldlr	
Thbd	Vtn	
Thra	Xcr1	
Thrap3		
Tnfrsf10b		
Tnfrsf11a		
Tnfrsf11b		
Tnfrsf12a		
Tnfrsf13c		
Tnfrsf14		
Tnfrsf17		
Tnfrsf18		
Tnfrsf1a		
Tnfrsf1b		
Tnfrsf25		
Tnfrsf4		
Tnfrsf8		
Tnfrsf9		
Tshr		
Unc5b		
Unc5c		
Microarrays		
DRGs (321)	SCGs (297)	
Uts2r		
Vipr1		
Vipr2		
Vldlr		
Vtn		
Xcr1		

Gene symbols of proteins identified by mass spectrometry exclusively on sensory neurons (DRGs), exclusively on sympathetic neurons (SCGs), or on both neuron types, which were identified from the protein lists shown in Table 7. Also shown are receptor mRNAs identified by microarrays as expressed by DRG and SCG neurons, defined using the updated ligand-receptor database (modified from Yuzwa et al., 2016). Only receptor mRNAs that had expression exceeding the cutoffs for each neuron type (DRGs; *Itgam*, 87% and SCGs; *Sorcs3*, 81%) are included. The total numbers of receptors in each column are indicated.

Cell-surface proteomics is relatively insensitive, and it ha5s previously been shown that more sensitive transcriptomic profiling can also be used to identify biologically relevant paracrine interactions (Johnston et al., 2016; Yuzwa et al., 2016; Voronova et al., 2017). We therefore complemented the proteomics by analyzing six and four independent biological replicates of cultured DRG and SCG RNA, respectively, on Affymetrix GeneChip Rat Gene 2.0 ST Arrays. To analyze these data, we defined an expression cutoff based on the proteomics data. Specifically, we identified the cell-surface receptor proteins with the lowest mRNA expression on the microarray for each neuron type and used those as the cutoffs. For sensory and sympathetic neurons, these were *Itgam* and *Sorcs3* mRNAs, respectively (expressed at 87% and 81% of total mRNAs; Extended Data Fig. 5-1*D*). When these thresholds were applied to the microarray data, there were 321 and 297 receptor mRNAs in sensory and sympathetic neurons, respectively (Table 8). Importantly, there was good correspondence between the proteomics and microarray data; *TrkA/Ntrk1*, *Bmpr2*, *Ret*, and *Igf2r* mRNAs were similarly expressed in both populations of neurons; *Rtn4r*, *Gfra3*, and *Acvr2a* mRNAs were enriched in sensory neurons (2.8-, 10-, and 3.1-fold enriched, respectively); and *Alk* mRNA was 9-fold enriched in sympathetic neurons (*p* < 0.05 FDR for differences).

### Computational modeling predicts that ligands deriving from multiple types of nerve cells, including mesenchymal cells, act on peripheral neurons

We performed computational modeling with the 143 injured nerve ligands and the sensory and sympathetic neuron receptors we had defined to predict how the injured nerve environment might regulate peripheral axon biology. This modeling predicted 122 and 125 potential unidirectional paracrine interactions between the injured nerve and sympathetic and sensory neurons, respectively (Fig. 5*C*,*D*; Table 9). Of these, cell-surface receptor protein expression was detected for 49 and 60 sympathetic and sensory neuron predicted interactions (Fig. 5*C*,*D*, blue boxes). Many predicted interactions involved known peripheral nerve ligands such as the neurotrophin and GDNF families. Notably, all but three ligands (GNRH1, CXCL1, and CXCL2) were predicted to act on both sympathetic and sensory neurons. The receptors for these predicted interactions were also largely the same, except for several sensory neuron receptors; the Erbb4 receptor for EGF/neuregulin family ligands, KDR for the VEGF family, ACVR1C for the activin/BMP family, and the CXCR5 chemokine receptor (Table 9). Thus, the injured nerve is predicted to produce ligands that act on both sympathetic and sensory neurons, largely through the same receptors.

**Table 9:** Ligand-receptor modeling between injured nerve ligands and sympathetic neurons (SCGs), sensory neurons (DRGs), motor neurons (MNs), and retinal ganglion cells (RGCs)

SCGs			
Source cell	Ligand	Target cell	Receptor
Injured nerve	ADM	SCGs	CALCRL
Injured nerve	ANGPT1	SCGs	TEK
Injured nerve	ANGPT2	SCGs	TEK
Injured nerve	ANGPT4	SCGs	TEK
Injured nerve	APLN	SCGs	APLNR
Injured nerve	ARTN	SCGs	GFRA3
Injured nerve	ARTN	SCGs	RET
Injured nerve	BDNF	SCGs	NTRK2
Injured nerve	BDNF	SCGs	SORT1
Injured nerve	BDNF	SCGs	NGFR
Injured nerve	BMP2	SCGs	BMPR1A
Injured nerve	BMP2	SCGs	BMPR1B
Injured nerve	BMP2	SCGs	BMPR2
Injured nerve	BMP2	SCGs	ENG
Injured nerve	BMP2	SCGs	NEO1
Injured nerve	BMP4	SCGs	BMPR1A
Injured nerve	BMP4	SCGs	BMPR1B
Injured nerve	BMP4	SCGs	BMPR2
Injured nerve	BMP4	SCGs	NEO1
Injured nerve	BMP5	SCGs	BMPR1A
Injured nerve	BMP7	SCGs	ACVR1
Injured nerve	BMP7	SCGs	ACVR2A
Injured nerve	BMP7	SCGs	ACVR2B
Injured nerve	BMP7	SCGs	BMPR1A
Injured nerve	BMP7	SCGs	BMPR1B
Injured nerve	BMP7	SCGs	BMPR2
Injured nerve	BMP7	SCGs	NEO1
Injured nerve	BTC	SCGs	EGFR
Injured nerve	BTC	SCGs	ERBB2
Injured nerve	CCK	SCGs	CCKAR
Injured nerve	CCK	SCGs	CCKBR
Injured nerve	CCL11	SCGs	CXCR3
Injured nerve	CCL19	SCGs	CCR10
Injured nerve	CCL19	SCGs	CCR7
Injured nerve	CCL2	SCGs	CCR10
Injured nerve	CCL25	SCGs	CCR10
Injured nerve	CCL3	SCGs	CCR4
Injured nerve	CCL5	SCGs	CCR4
Injured nerve	CCL5	SCGs	CXCR3
Injured nerve	CCL5	SCGs	SDC4
Injured nerve	CCL7	SCGs	CCR10
Injured nerve	CCL7	SCGs	CXCR3
Injured nerve	CLCF1	SCGs	CNTFR
Injured nerve	CLCF1	SCGs	IL6ST
Injured nerve	CLCF1	SCGs	LIFR
Injured nerve	CRLF1	SCGs	CNTFR
Injured nerve	CRLF1	SCGs	IL6ST
Injured nerve	CRLF1	SCGs	LIFR
Injured nerve	CSF1	SCGs	CSF1R
Injured nerve	CX3CL1	SCGs	CX3CR1
Injured nerve	CXCL10	SCGs	CXCR3
Injured nerve	CXCL12	SCGs	CCR4
Injured nerve	CXCL12	SCGs	CXCR4
Injured nerve	CXCL13	SCGs	CCR10
Injured nerve	CXCL13	SCGs	CXCR3
Injured nerve	CXCL9	SCGs	CXCR3
Injured nerve	DHH	SCGs	PTCH1
Injured nerve	DHH	SCGs	PTCH2
Injured nerve	DLL1	SCGs	NOTCH1
Injured nerve	DLL1	SCGs	NOTCH2
SCGs			
Source cell	Ligand	Target cell	Receptor
Injured nerve	DLL1	SCGs	NOTCH3
Injured nerve	DLL4	SCGs	NOTCH1
Injured nerve	EBI3	SCGs	IL27RA
Injured nerve	EDA	SCGs	EDAR
Injured nerve	EDN3	SCGs	EDNRA
Injured nerve	EDN3	SCGs	EDNRB
Injured nerve	EFNA1	SCGs	EPHA1
Injured nerve	EFNA1	SCGs	EPHA3
Injured nerve	EFNA2	SCGs	EPHA3
Injured nerve	EFNA4	SCGs	EPHA3
Injured nerve	EFNA4	SCGs	EPHA5
Injured nerve	EFNA5	SCGs	EPHA3
Injured nerve	EFNA5	SCGs	EPHA2
Injured nerve	EFNA5	SCGs	EPHA7
Injured nerve	EFNA5	SCGs	EPHA5
Injured nerve	EFNB1	SCGs	EPHB2
Injured nerve	EFNB2	SCGs	EPHB1
Injured nerve	EFNB2	SCGs	EPHA4
Injured nerve	EFNB2	SCGs	EPHA3
Injured nerve	EFNB2	SCGs	EPHB4
Injured nerve	EFNB2	SCGs	EPHB2
Injured nerve	FGF1	SCGs	FGFR1
Injured nerve	FGF1	SCGs	FGFR2
Injured nerve	FGF1	SCGs	FGFR3
Injured nerve	FGF1	SCGs	FGFR4
Injured nerve	FGF10	SCGs	FGFR2
Injured nerve	FGF18	SCGs	FGFR4
Injured nerve	FGF5	SCGs	FGFR1
Injured nerve	FGF5	SCGs	FGFR3
Injured nerve	FGF7	SCGs	FGFR2
Injured nerve	FGF7	SCGs	NRP1
Injured nerve	FIGF	SCGs	FLT4
Injured nerve	FIGF	SCGs	NRP1
Injured nerve	FIGF	SCGs	NRP2
Injured nerve	FSTL1	SCGs	CD14
Injured nerve	FSTL1	SCGs	DIP2A
Injured nerve	GAS6	SCGs	AXL
Injured nerve	GDF11	SCGs	ACVR1B
Injured nerve	GDF11	SCGs	ACVR2B
Injured nerve	GDNF	SCGs	GFRA2
Injured nerve	GDNF	SCGs	RET
Injured nerve	HBEGF	SCGs	EGFR
Injured nerve	HGF	SCGs	MET
Injured nerve	IGF1	SCGs	IGF1R
Injured nerve	IGF1	SCGs	IGFBP1
Injured nerve	IGF1	SCGs	IGFBP2
Injured nerve	IGF1	SCGs	IGFBP3
Injured nerve	IGF1	SCGs	IGFBP4
Injured nerve	IGF1	SCGs	IGFBP5
Injured nerve	IGF1	SCGs	IGFBP6
Injured nerve	IGF1	SCGs	IGFBP7
Injured nerve	IGF1	SCGs	INSR
Injured nerve	IGF2	SCGs	IGF1R
Injured nerve	IGF2	SCGs	IGF2R
Injured nerve	IGF2	SCGs	INSR
Injured nerve	IL15	SCGs	IL15RA
Injured nerve	IL15	SCGs	IL2RB
Injured nerve	IL15	SCGs	IL2RG
Injured nerve	IL16	SCGs	CD4
Injured nerve	IL16	SCGs	GRIN2A
Injured nerve	IL16	SCGs	GRIN2B
SCGs			
Source cell	Ligand	Target cell	Receptor
Injured nerve	IL16	SCGs	GRIN2C
Injured nerve	IL16	SCGs	GRIN2D
Injured nerve	IL18	SCGs	IL18R1
Injured nerve	IL18	SCGs	IL18RAP
Injured nerve	IL1B	SCGs	IL1R1
Injured nerve	IL1B	SCGs	IL1R2
Injured nerve	IL1B	SCGs	IL1RAP
Injured nerve	IL33	SCGs	IL1RL1
Injured nerve	IL6	SCGs	IL6R
Injured nerve	IL6	SCGs	IL6ST
Injured nerve	INHA	SCGs	ACVR2A
Injured nerve	INHA	SCGs	TGFBR3
Injured nerve	INHBA	SCGs	ACVR1B
Injured nerve	INHBA	SCGs	ACVR2A
Injured nerve	INHBA	SCGs	ACVR2B
Injured nerve	INHBB	SCGs	ACVR1
Injured nerve	INHBB	SCGs	ACVR1B
Injured nerve	INHBB	SCGs	ACVR2A
Injured nerve	INHBB	SCGs	ACVR2B
Injured nerve	JAG1	SCGs	NOTCH1
Injured nerve	JAG1	SCGs	NOTCH2
Injured nerve	JAG1	SCGs	NOTCH3
Injured nerve	JAG2	SCGs	NOTCH1
Injured nerve	JAG2	SCGs	NOTCH2
Injured nerve	JAG2	SCGs	NOTCH3
Injured nerve	LIF	SCGs	IL6ST
Injured nerve	LIF	SCGs	LIFR
Injured nerve	LTB	SCGs	LTBR
Injured nerve	MDK	SCGs	ALK
Injured nerve	MDK	SCGs	LRP1
Injured nerve	MDK	SCGs	LRP2
Injured nerve	MDK	SCGs	PTPRZ1
Injured nerve	MIF	SCGs	CD74
Injured nerve	NGF	SCGs	NGFR
Injured nerve	NGF	SCGs	NTRK1
Injured nerve	NGF	SCGs	SORCS3
Injured nerve	NGF	SCGs	SORT1
Injured nerve	NOV	SCGs	NOTCH1
Injured nerve	NPPC	SCGs	NPR2
Injured nerve	NPPC	SCGs	NPR3
Injured nerve	NTF3	SCGs	NGFR
Injured nerve	NTF3	SCGs	NTRK1
Injured nerve	NTF3	SCGs	NTRK2
Injured nerve	NTF3	SCGs	NTRK3
Injured nerve	NTN1	SCGs	DCC
Injured nerve	NTN1	SCGs	NEO1
Injured nerve	NTN1	SCGs	UNC5B
Injured nerve	NTN1	SCGs	UNC5C
Injured nerve	OSM	SCGs	IL6ST
Injured nerve	OSM	SCGs	LIFR
Injured nerve	OSM	SCGs	OSMR
Injured nerve	PDGFA	SCGs	PDGFRA
Injured nerve	PDGFB	SCGs	PDGFRA
Injured nerve	PDGFB	SCGs	PDGFRB
Injured nerve	PDGFC	SCGs	PDGFRA
Injured nerve	PF4	SCGs	CXCR3
Injured nerve	PF4	SCGs	LDLR
Injured nerve	PF4	SCGs	THBD
Injured nerve	PGF	SCGs	FLT1
Injured nerve	PGF	SCGs	NRP1
Injured nerve	PGF	SCGs	NRP2
SCGs			
Source cell	Ligand	Target cell	Receptor
Injured nerve	POMC	SCGs	MC1R
Injured nerve	POMC	SCGs	MC2R
Injured nerve	POMC	SCGs	MC3R
Injured nerve	POMC	SCGs	MC4R
Injured nerve	POMC	SCGs	MC5R
Injured nerve	PTHLH	SCGs	PTH1R
Injured nerve	PTN	SCGs	ALK
Injured nerve	PTN	SCGs	PTPRS
Injured nerve	PTN	SCGs	PTPRZ1
Injured nerve	RSPO1	SCGs	LGR5
Injured nerve	RTN4	SCGs	LINGO1
Injured nerve	RTN4	SCGs	RTN4R
Injured nerve	RTN4	SCGs	RTN4RL1
Injured nerve	SEMA3B	SCGs	PLXNA1
Injured nerve	SEMA3B	SCGs	PLXNA2
Injured nerve	SEMA3B	SCGs	PLXNA3
Injured nerve	SEMA3B	SCGs	PLXNA4
Injured nerve	SEMA3B	SCGs	PLXND1
Injured nerve	SEMA3C	SCGs	PLXNA1
Injured nerve	SEMA3C	SCGs	PLXNA2
Injured nerve	SEMA3C	SCGs	PLXNA3
Injured nerve	SEMA3C	SCGs	PLXNA4
Injured nerve	SEMA3C	SCGs	PLXND1
Injured nerve	SEMA3D	SCGs	PLXNA1
Injured nerve	SEMA3D	SCGs	PLXNA2
Injured nerve	SEMA3D	SCGs	PLXNA3
Injured nerve	SEMA3D	SCGs	PLXNA4
Injured nerve	SEMA3D	SCGs	PLXND1
Injured nerve	SEMA3E	SCGs	PLXNA1
Injured nerve	SEMA3E	SCGs	PLXNA2
Injured nerve	SEMA3E	SCGs	PLXNA3
Injured nerve	SEMA3E	SCGs	PLXNA4
Injured nerve	SEMA3E	SCGs	PLXND1
Injured nerve	SEMA3F	SCGs	PLXNA1
Injured nerve	SEMA3F	SCGs	PLXNA2
Injured nerve	SEMA3F	SCGs	PLXNA3
Injured nerve	SEMA3F	SCGs	PLXNA4
Injured nerve	SEMA3F	SCGs	PLXND1
Injured nerve	SEMA3G	SCGs	PLXNA1
Injured nerve	SEMA3G	SCGs	PLXNA2
Injured nerve	SEMA3G	SCGs	PLXNA3
Injured nerve	SEMA3G	SCGs	PLXNA4
Injured nerve	SEMA3G	SCGs	PLXND1
Injured nerve	SEMA4A	SCGs	PLXNB1
Injured nerve	SEMA4A	SCGs	PLXNC1
Injured nerve	SEMA4A	SCGs	PLXND1
Injured nerve	SEMA4B	SCGs	PLXNB1
Injured nerve	SEMA4B	SCGs	PLXNC1
Injured nerve	SEMA4C	SCGs	PLXNB1
Injured nerve	SEMA4C	SCGs	PLXNC1
Injured nerve	SEMA4D	SCGs	PLXNB1
Injured nerve	SEMA4D	SCGs	PLXNC1
Injured nerve	SEMA4F	SCGs	PLXNB1
Injured nerve	SEMA4F	SCGs	PLXNC1
Injured nerve	SEMA5A	SCGs	PLXNA3
Injured nerve	SEMA5A	SCGs	PLXNA4
Injured nerve	SEMA5A	SCGs	PLXNC1
Injured nerve	SEMA5B	SCGs	PLXNA3
Injured nerve	SEMA5B	SCGs	PLXNA4
Injured nerve	SEMA5B	SCGs	PLXNC1
Injured nerve	SEMA6A	SCGs	PLXNA1
SCGs			
Source cell	Ligand	Target cell	Receptor
Injured nerve	SEMA6A	SCGs	PLXNA2
Injured nerve	SEMA6B	SCGs	PLXNA1
Injured nerve	SEMA6C	SCGs	PLXNA1
Injured nerve	SEMA6D	SCGs	PLXNA1
Injured nerve	SEMA7A	SCGs	PLXNC1
Injured nerve	SHH	SCGs	PTCH1
Injured nerve	SHH	SCGs	PTCH2
Injured nerve	TGFA	SCGs	EGFR
Injured nerve	TGFA	SCGs	ERBB2
Injured nerve	TGFB1	SCGs	ACVRL1
Injured nerve	TGFB1	SCGs	ENG
Injured nerve	TGFB1	SCGs	TGFBR2
Injured nerve	TGFB1	SCGs	TGFBR3
Injured nerve	TGFB2	SCGs	TGFBR2
Injured nerve	TGFB2	SCGs	TGFBR3
Injured nerve	TGFB3	SCGs	ACVRL1
Injured nerve	TGFB3	SCGs	TGFBR2
Injured nerve	TNF	SCGs	TNFRSF1A
Injured nerve	TNF	SCGs	TNFRSF1B
Injured nerve	TNFSF10	SCGs	TNFRSF10B
Injured nerve	TNFSF12	SCGs	TNFRSF12A
Injured nerve	TNFSF12	SCGs	TNFRSF25
Injured nerve	TNFSF14	SCGs	LTBR
Injured nerve	TNFSF14	SCGs	TNFRSF14
Injured nerve	TNFSF8	SCGs	TNFRSF8
Injured nerve	TNFSF9	SCGs	TNFRSF9
Injured nerve	TSLP	SCGs	CRLF2
Injured nerve	TSLP	SCGs	IL7R
Injured nerve	UCN2	SCGs	CRHR2
Injured nerve	VEGFA	SCGs	FLT1
Injured nerve	VEGFA	SCGs	NRP1
Injured nerve	VEGFA	SCGs	NRP2
Injured nerve	VEGFB	SCGs	FLT1
Injured nerve	VEGFB	SCGs	NRP1
Injured nerve	VEGFC	SCGs	FLT4
Injured nerve	VEGFC	SCGs	NRP1
Injured nerve	VEGFC	SCGs	NRP2
Injured nerve	WNT11	SCGs	FZD4
Injured nerve	WNT2	SCGs	FZD1
Injured nerve	WNT2	SCGs	FZD9
Injured nerve	WNT5A	SCGs	FZD2
Injured nerve	WNT5A	SCGs	FZD5
Injured nerve Injured Nerve	WNT5A WNT5A	SCGs SCGs	ROR1 ROR2
DRGs			
Source cell	Ligand	Target cell	Receptor
Injured nerve	ADM	DRGs	CALCRL
Injured nerve	ANGPT1	DRGs	TEK
Injured nerve	ANGPT2	DRGs	TEK
Injured nerve	ANGPT4	DRGs	TEK
Injured nerve	APLN	DRGs	APLNR
Injured nerve	ARTN	DRGs	GFRA3
Injured nerve	ARTN	DRGs	RET
Injured nerve	BDNF	DRGs	NGFR
Injured nerve	BDNF	DRGs	NTRK2
Injured nerve	BDNF	DRGs	SORT1
Injured nerve	BMP2	DRGs	BMPR1A
Injured nerve	BMP2	DRGs	BMPR1B
Injured nerve	BMP2	DRGs	BMPR2
Injured nerve	BMP2	DRGs	ENG
Injured nerve	BMP2	DRGs	NEO1
DRGs			
Source cell	Ligand	Target cell	Receptor
Injured nerve	BMP4	DRGs	BMPR1A
Injured nerve	BMP4	DRGs	BMPR1B
Injured nerve	BMP4	DRGs	BMPR2
Injured nerve	BMP4	DRGs	NEO1
Injured nerve	BMP5	DRGs	BMPR1A
Injured nerve	BMP7	DRGs	ACVR1
Injured nerve	BMP7	DRGs	ACVR2A
Injured nerve	BMP7	DRGs	ACVR2B
Injured nerve	BMP7	DRGs	BMPR1A
Injured nerve	BMP7	DRGs	BMPR1B
Injured nerve	BMP7	DRGs	BMPR2
Injured nerve	BMP7	DRGs	NEO1
Injured nerve	BTC	DRGs	EGFR
Injured nerve	BTC	DRGs	ERBB2
Injured nerve	BTC	DRGs	ERBB4
Injured nerve	CCK	DRGs	CCKAR
Injured nerve	CCK	DRGs	CCKBR
Injured nerve	CCL11	DRGs	CXCR3
Injured nerve	CCL19	DRGs	CCR10
Injured nerve	CCL19	DRGs	CCR7
Injured nerve	CCL2	DRGs	CCR10
Injured nerve	CCL25	DRGs	CCR10
Injured nerve	CCL3	DRGs	CCR4
Injured nerve	CCL5	DRGs	CCR4
Injured nerve	CCL5	DRGs	CXCR3
Injured nerve	CCL5	DRGs	SDC4
Injured nerve	CCL7	DRGs	CCR10
Injured nerve	CCL7	DRGs	CXCR3
Injured nerve	CLCF1	DRGs	CNTFR
Injured nerve	CLCF1	DRGs	IL6ST
Injured nerve	CLCF1	DRGs	LIFR
Injured nerve	CRLF1	DRGs	CNTFR
Injured nerve	CRLF1	DRGs	IL6ST
Injured nerve	CRLF1	DRGs	LIFR
Injured nerve	CSF1	DRGs	CSF1R
Injured nerve	CX3CL1	DRGs	CX3CR1
Injured nerve	CXCL1	DRGs	CXCR2
Injured nerve	CXCL10	DRGs	CXCR3
Injured nerve	CXCL12	DRGs	CCR4
Injured nerve	CXCL12	DRGs	CXCR4
Injured nerve	CXCL13	DRGs	CCR10
Injured nerve	CXCL13	DRGs	CXCR3
Injured nerve	CXCL13	DRGs	CXCR5
Injured nerve	CXCL2	DRGs	CXCR2
Injured nerve	CXCL9	DRGs	CXCR3
Injured nerve	DHH	DRGs	PTCH1
Injured nerve	DHH	DRGs	PTCH2
Injured nerve	DLL1	DRGs	NOTCH1
Injured nerve	DLL1	DRGs	NOTCH2
Injured nerve	DLL1	DRGs	NOTCH3
Injured nerve	DLL4	DRGs	NOTCH1
Injured nerve	EBI3	DRGs	IL27RA
Injured nerve	EDA	DRGs	EDAR
Injured nerve	EDN3	DRGs	EDNRA
Injured nerve	EDN3	DRGs	EDNRB
Injured nerve	EFNA1	DRGs	EPHA1
Injured nerve	EFNA1	DRGs	EPHA3
Injured nerve	EFNA2	DRGs	EPHA3
Injured nerve	EFNA4	DRGs	EPHA3
Injured nerve	EFNA4	DRGs	EPHA5
Injured nerve	EFNA5	DRGs	EPHA3
DRGs			
Source cell	Ligand	Target cell	Receptor
Injured nerve	EFNA5	DRGs	EPHA2
Injured nerve	EFNA5	DRGs	EPHA7
Injured nerve	EFNA5	DRGs	EPHA5
Injured nerve	EFNB1	DRGs	EPHB2
Injured nerve	EFNB2	DRGs	EPHB1
Injured nerve	EFNB2	DRGs	EPHA4
Injured nerve	EFNB2	DRGs	EPHA3
Injured nerve	EFNB2	DRGs	EPHB4
Injured nerve	EFNB2	DRGs	EPHB2
Injured nerve	FGF1	DRGs	FGFR1
Injured nerve	FGF1	DRGs	FGFR2
Injured nerve	FGF1	DRGs	FGFR3
Injured nerve	FGF1	DRGs	FGFR4
Injured nerve	FGF10	DRGs	FGFR2
Injured nerve	FGF18	DRGs	FGFR4
Injured nerve	FGF5	DRGs	FGFR1
Injured nerve	FGF5	DRGs	FGFR3
Injured nerve	FGF7	DRGs	FGFR2
Injured nerve	FGF7	DRGs	NRP1
Injured nerve	FIGF	DRGs	FLT4
Injured nerve	FIGF	DRGs	KDR
Injured nerve	FIGF	DRGs	NRP1
Injured nerve	FIGF	DRGs	NRP2
Injured nerve	FSTL1	DRGs	CD14
Injured nerve	FSTL1	DRGs	DIP2A
Injured nerve	GAS6	DRGs	AXL
Injured nerve	GDF11	DRGs	ACVR1B
Injured nerve	GDF11	DRGs	ACVR1C
Injured nerve	GDF11	DRGs	ACVR2B
Injured nerve	GDNF	DRGs	GFRA2
Injured nerve	GDNF	DRGs	RET
Injured nerve	GNRH1	DRGs	GNRHR
Injured nerve	HBEGF	DRGs	EGFR
Injured nerve	HBEGF	DRGs	ERBB4
Injured nerve	HGF	DRGs	MET
Injured nerve	IGF1	DRGs	IGF1R
Injured nerve	IGF1	DRGs	IGFBP1
Injured nerve	IGF1	DRGs	IGFBP2
Injured nerve	IGF1	DRGs	IGFBP3
Injured nerve	IGF1	DRGs	IGFBP4
Injured nerve	IGF1	DRGs	IGFBP5
Injured nerve	IGF1	DRGs	IGFBP6
Injured nerve	IGF1	DRGs	IGFBP7
Injured nerve	IGF1	DRGs	INSR
Injured nerve	IGF2	DRGs	IGF1R
Injured nerve	IGF2	DRGs	IGF2R
Injured nerve	IGF2	DRGs	INSR
Injured nerve	IL15	DRGs	IL15RA
Injured nerve	IL15	DRGs	IL2RB
Injured nerve	IL15	DRGs	IL2RG
Injured nerve	IL16	DRGs	CD4
Injured nerve	IL16	DRGs	GRIN2A
Injured nerve	IL16	DRGs	GRIN2B
Injured nerve	IL16	DRGs	GRIN2C
Injured nerve	IL16	DRGs	GRIN2D
Injured nerve	IL18	DRGs	IL18R1
Injured nerve	IL18	DRGs	IL18RAP
Injured nerve	IL1B	DRGs	IL1R1
Injured nerve	IL1B	DRGs	IL1R2
Injured nerve	IL1B	DRGs	IL1RAP
Injured nerve	IL33	DRGs	IL1RL1
DRGs			
Source cell	Ligand	Target cell	Receptor
Injured nerve	IL6	DRGs	IL6R
Injured nerve	IL6	DRGs	IL6ST
Injured nerve	INHA	DRGs	ACVR2A
Injured nerve	INHA	DRGs	TGFBR3
Injured nerve	INHBA	DRGs	ACVR1B
Injured nerve	INHBA	DRGs	ACVR2A
Injured nerve	INHBA	DRGs	ACVR2B
Injured nerve	INHBB	DRGs	ACVR1
Injured nerve	INHBB	DRGs	ACVR1B
Injured nerve	INHBB	DRGs	ACVR1C
Injured nerve	INHBB	DRGs	ACVR2A
Injured nerve	INHBB	DRGs	ACVR2B
Injured nerve	JAG1	DRGs	NOTCH1
Injured nerve	JAG1	DRGs	NOTCH2
Injured nerve	JAG1	DRGs	NOTCH3
Injured nerve	JAG2	DRGs	NOTCH1
Injured nerve	JAG2	DRGs	NOTCH2
Injured nerve	JAG2	DRGs	NOTCH3
Injured nerve	LIF	DRGs	IL6ST
Injured nerve	LIF	DRGs	LIFR
Injured nerve	LTB	DRGs	LTBR
Injured nerve	MDK	DRGs	ALK
Injured nerve	MDK	DRGs	LRP1
Injured nerve	MDK	DRGs	LRP2
Injured nerve	MDK	DRGs	PTPRZ1
Injured nerve	MIF	DRGs	CD74
Injured nerve	NGF	DRGs	NGFR
Injured nerve	NGF	DRGs	NTRK1
Injured nerve	NGF	DRGs	SORCS3
Injured nerve	NGF	DRGs	SORT1
Injured nerve	NOV	DRGs	NOTCH1
Injured nerve	NPPC	DRGs	NPR2
Injured nerve	NPPC	DRGs	NPR3
Injured nerve	NTF3	DRGs	NGFR
Injured nerve	NTF3	DRGs	NTRK1
Injured nerve	NTF3	DRGs	NTRK2
Injured nerve	NTF3	DRGs	NTRK3
Injured nerve	NTN1	DRGs	DCC
Injured nerve	NTN1	DRGs	NEO1
Injured nerve	NTN1	DRGs	UNC5B
Injured nerve	NTN1	DRGs	UNC5C
Injured nerve	OSM	DRGs	IL6ST
Injured nerve	OSM	DRGs	LIFR
Injured nerve	OSM	DRGs	OSMR
Injured nerve	PDGFA	DRGs	PDGFRA
Injured nerve	PDGFB	DRGs	PDGFRA
Injured nerve	PDGFB	DRGs	PDGFRB
Injured nerve	PDGFC	DRGs	PDGFRA
Injured nerve	PF4	DRGs	CXCR3
Injured nerve	PF4	DRGs	LDLR
Injured nerve	PF4	DRGs	THBD
Injured nerve	PGF	DRGs	FLT1
Injured nerve	PGF	DRGs	NRP1
Injured nerve	PGF	DRGs	NRP2
Injured nerve	POMC	DRGs	MC1R
Injured nerve	POMC	DRGs	MC2R
Injured nerve	POMC	DRGs	MC3R
Injured nerve	POMC	DRGs	MC4R
Injured nerve	POMC	DRGs	MC5R
Injured nerve	PTHLH	DRGs	PTH1R
Injured nerve	PTN	DRGs	ALK
DRGs			
Source cell	Ligand	Target cell	Receptor
Injured nerve	PTN	DRGs	PTPRS
Injured nerve	PTN	DRGs	PTPRZ1
Injured nerve	RSPO1	DRGs	LGR5
Injured nerve	RTN4	DRGs	LINGO1
Injured nerve	RTN4	DRGs	RTN4R
Injured nerve	RTN4	DRGs	RTN4RL1
Injured nerve	SEMA3B	DRGs	PLXNA1
Injured nerve	SEMA3B	DRGs	PLXNA2
Injured nerve	SEMA3B	DRGs	PLXNA3
Injured nerve	SEMA3B	DRGs	PLXNA4
Injured nerve	SEMA3B	DRGs	PLXND1
Injured nerve	SEMA3C	DRGs	PLXNA1
Injured nerve	SEMA3C	DRGs	PLXNA2
Injured nerve	SEMA3C	DRGs	PLXNA3
Injured nerve	SEMA3C	DRGs	PLXNA4
Injured nerve	SEMA3C	DRGs	PLXND1
Injured nerve	SEMA3D	DRGs	PLXNA1
Injured nerve	SEMA3D	DRGs	PLXNA2
Injured nerve	SEMA3D	DRGs	PLXNA3
Injured nerve	SEMA3D	DRGs	PLXNA4
Injured nerve	SEMA3D	DRGs	PLXND1
Injured nerve	SEMA3E	DRGs	PLXNA1
Injured nerve	SEMA3E	DRGs	PLXNA2
Injured nerve	SEMA3E	DRGs	PLXNA3
Injured nerve	SEMA3E	DRGs	PLXNA4
Injured nerve	SEMA3E	DRGs	PLXND1
Injured nerve	SEMA3F	DRGs	PLXNA1
Injured nerve	SEMA3F	DRGs	PLXNA2
Injured nerve	SEMA3F	DRGs	PLXNA3
Injured nerve	SEMA3F	DRGs	PLXNA4
Injured nerve	SEMA3F	DRGs	PLXND1
Injured nerve	SEMA3G	DRGs	PLXNA1
Injured nerve	SEMA3G	DRGs	PLXNA2
Injured nerve	SEMA3G	DRGs	PLXNA3
Injured nerve	SEMA3G	DRGs	PLXNA4
Injured nerve	SEMA3G	DRGs	PLXND1
Injured nerve	SEMA4A	DRGs	PLXNB1
Injured nerve	SEMA4A	DRGs	PLXNC1
Injured nerve	SEMA4A	DRGs	PLXND1
Injured nerve	SEMA4B	DRGs	PLXNB1
Injured nerve	SEMA4B	DRGs	PLXNC1
Injured nerve	SEMA4C	DRGs	PLXNB1
Injured nerve	SEMA4C	DRGs	PLXNC1
Injured nerve	SEMA4D	DRGs	PLXNB1
Injured nerve	SEMA4D	DRGs	PLXNC1
Injured nerve	SEMA4F	DRGs	PLXNB1
Injured nerve	SEMA4F	DRGs	PLXNC1
Injured nerve	SEMA5A	DRGs	PLXNA3
Injured nerve	SEMA5A	DRGs	PLXNA4
Injured nerve	SEMA5A	DRGs	PLXNC1
Injured nerve	SEMA5B	DRGs	PLXNA3
Injured nerve	SEMA5B	DRGs	PLXNA4
Injured nerve	SEMA5B	DRGs	PLXNC1
Injured nerve	SEMA6A	DRGs	PLXNA1
Injured nerve	SEMA6A	DRGs	PLXNA2
Injured nerve	SEMA6B	DRGs	PLXNA1
Injured nerve	SEMA6C	DRGs	PLXNA1
Injured nerve	SEMA6D	DRGs	PLXNA1
Injured nerve	SEMA7A	DRGs	PLXNC1
Injured nerve	SHH	DRGs	PTCH1
Injured nerve	SHH	DRGs	PTCH2
DRGs			
Source cell	Ligand	Target cell	Receptor
Injured nerve	TGFA	DRGs	EGFR
Injured nerve	TGFA	DRGs	ERBB2
Injured nerve	TGFB1	DRGs	ACVRL1
Injured nerve	TGFB1	DRGs	ENG
Injured nerve	TGFB1	DRGs	TGFBR2
Injured nerve	TGFB1	DRGs	TGFBR3
Injured nerve	TGFB2	DRGs	TGFBR2
Injured nerve	TGFB2	DRGs	TGFBR3
Injured nerve	TGFB3	DRGs	ACVRL1
Injured nerve	TGFB3	DRGs	TGFBR2
Injured nerve	TNF	DRGs	TNFRSF1A
Injured nerve	TNF	DRGs	TNFRSF1B
Injured nerve	TNFSF10	DRGs	TNFRSF10B
Injured nerve	TNFSF12	DRGs	TNFRSF12A
Injured nerve	TNFSF12	DRGs	TNFRSF25
Injured nerve	TNFSF14	DRGs	LTBR
Injured nerve	TNFSF14	DRGs	TNFRSF14
Injured nerve	TNFSF8	DRGs	TNFRSF8
Injured nerve	TNFSF9	DRGs	TNFRSF9
Injured nerve	TSLP	DRGs	CRLF2
Injured nerve	TSLP	DRGs	IL7R
Injured nerve	UCN2	DRGs	CRHR2
Injured nerve	VEGFA	DRGs	FLT1
Injured nerve	VEGFA	DRGs	KDR
Injured nerve	VEGFA	DRGs	NRP1
Injured nerve	VEGFA	DRGs	NRP2
Injured nerve	VEGFB	DRGs	FLT1
Injured nerve	VEGFB	DRGs	NRP1
Injured nerve	VEGFC	DRGs	FLT4
Injured nerve	VEGFC	DRGs	KDR
Injured nerve	VEGFC	DRGs	NRP1
Injured nerve	VEGFC	DRGs	NRP2
Injured nerve	WNT11	DRGs	FZD4
Injured nerve	WNT2	DRGs	FZD1
Injured nerve	WNT2	DRGs	FZD9
Injured nerve	WNT5A	DRGs	FZD2
Injured nerve	WNT5A	DRGs	FZD5
Injured nerve	WNT5A	DRGs	ROR1
Injured nerve	WNT5A	DRGs	ROR2
MNs			
Source cell	Ligand	Target cell	Receptor
Injured nerve	ADM	MNs	CALCRL
Injured nerve	ANGPT1	MNs	TEK
Injured nerve	ANGPT2	MNs	TEK
Injured nerve	ANGPT4	MNs	TEK
Injured nerve	APLN	MNs	APLNR
Injured nerve	ARTN	MNs	GFRA3
Injured nerve	ARTN	MNs	RET
Injured nerve	BDNF	MNs	NGFR
Injured nerve	BDNF	MNs	NTRK2
Injured nerve	BDNF	MNs	SORT1
Injured nerve	BMP2	MNs	BMPR1A
Injured nerve	BMP2	MNs	BMPR1B
Injured nerve	BMP2	MNs	BMPR2
Injured nerve	BMP2	MNs	ENG
Injured nerve	BMP2	MNs	NEO1
Injured nerve	BMP4	MNs	BMPR1A
Injured nerve	BMP4	MNs	BMPR1B
Injured nerve	BMP4	MNs	BMPR2
Injured nerve	BMP4	MNs	NEO1
Injured nerve	BMP5	MNs	BMPR1A
MNs			
Source cell	Ligand	Target cell	Receptor
Injured nerve	BMP7	MNs	ACVR1
Injured nerve	BMP7	MNs	ACVR2A
Injured nerve	BMP7	MNs	ACVR2B
Injured nerve	BMP7	MNs	BMPR1A
Injured nerve	BMP7	MNs	BMPR1B
Injured nerve	BMP7	MNs	BMPR2
Injured nerve	BMP7	MNs	NEO1
Injured nerve	BTC	MNs	EGFR
Injured nerve	BTC	MNs	ERBB2
Injured nerve	BTC	MNs	ERBB4
Injured nerve	CCK	MNs	CCKAR
Injured nerve	CCK	MNs	CCKBR
Injured nerve	CCL11	MNs	CCR5
Injured nerve	CCL11	MNs	CXCR3
Injured nerve	CCL19	MNs	CCR10
Injured nerve	CCL2	MNs	CCR10
Injured nerve	CCL2	MNs	CCR2
Injured nerve	CCL25	MNs	CCR10
Injured nerve	CCL25	MNs	CCR9
Injured nerve	CCL3	MNs	CCR5
Injured nerve	CCL5	MNs	CCR5
Injured nerve	CCL5	MNs	CXCR3
Injured nerve	CCL5	MNs	SDC4
Injured nerve	CCL7	MNs	CCR10
Injured nerve	CCL7	MNs	CCR2
Injured nerve	CCL7	MNs	CCR5
Injured nerve	CCL7	MNs	CXCR3
Injured nerve	CLCF1	MNs	CNTFR
Injured nerve	CLCF1	MNs	IL6ST
Injured nerve	CLCF1	MNs	LIFR
Injured nerve	CRLF1	MNs	CNTFR
Injured nerve	CRLF1	MNs	IL6ST
Injured nerve	CRLF1	MNs	LIFR
Injured nerve	CSF1	MNs	CSF1R
Injured nerve	CX3CL1	MNs	CX3CR1
Injured nerve	CXCL10	MNs	CXCR3
Injured nerve	CXCL12	MNs	CXCR4
Injured nerve	CXCL13	MNs	CCR10
Injured nerve	CXCL13	MNs	CXCR3
Injured nerve	CXCL13	MNs	CXCR5
Injured nerve	CXCL9	MNs	CXCR3
Injured nerve	DHH	MNs	PTCH1
Injured nerve	DHH	MNs	PTCH2
Injured nerve	DLL1	MNs	NOTCH1
Injured nerve	DLL1	MNs	NOTCH2
Injured nerve	DLL1	MNs	NOTCH3
Injured nerve	DLL4	MNs	NOTCH1
Injured nerve	EBI3	MNs	IL27RA
Injured nerve	EDN3	MNs	EDNRA
Injured nerve	EDN3	MNs	EDNRB
Injured nerve	EFNA1	MNs	EPHA1
Injured nerve	EFNA1	MNs	EPHA3
Injured nerve	EFNA2	MNs	EPHA3
Injured nerve	EFNA4	MNs	EPHA3
Injured nerve	EFNA4	MNs	EPHA5
Injured nerve	EFNA5	MNs	EPHA3
Injured nerve	EFNA5	MNs	EPHA2
Injured nerve	EFNA5	MNs	EPHA7
Injured nerve	EFNA5	MNs	EPHA5
Injured nerve	EFNB1	MNs	EPHB2
Injured nerve	EFNB2	MNs	EPHB1
MNs			
Source cell	Ligand	Target cell	Receptor
Injured nerve	EFNB2	MNs	EPHA4
Injured nerve	EFNB2	MNs	EPHA3
Injured nerve	EFNB2	MNs	EPHB4
Injured nerve	EFNB2	MNs	EPHB2
Injured nerve	FGF1	MNs	FGFR1
Injured nerve	FGF1	MNs	FGFR2
Injured nerve	FGF1	MNs	FGFR3
Injured nerve	FGF1	MNs	FGFR4
Injured nerve	FGF10	MNs	FGFR2
Injured nerve	FGF18	MNs	FGFR4
Injured nerve	FGF5	MNs	FGFR1
Injured nerve	FGF5	MNs	FGFR3
Injured nerve	FGF7	MNs	FGFR2
Injured nerve	FGF7	MNs	NRP1
Injured nerve	FIGF	MNs	FLT4
Injured nerve	FIGF	MNs	KDR
Injured nerve	FIGF	MNs	NRP1
Injured nerve	FIGF	MNs	NRP2
Injured nerve	FSTL1	MNs	CD14
Injured nerve	FSTL1	MNs	DIP2A
Injured nerve	GAS6	MNs	AXL
Injured nerve	GDF11	MNs	ACVR1B
Injured nerve	GDF11	MNs	ACVR1C
Injured nerve	GDF11	MNs	ACVR2B
Injured nerve	GDNF	MNs	GFRA1
Injured nerve	GDNF	MNs	GFRA2
Injured nerve	GDNF	MNs	RET
Injured nerve	GNRH1	MNs	GNRHR
Injured nerve	GRP	MNs	GRPR
Injured nerve	HBEGF	MNs	EGFR
Injured nerve	HBEGF	MNs	ERBB4
Injured nerve	HGF	MNs	MET
Injured nerve	IGF1	MNs	IGF1R
Injured nerve	IGF1	MNs	IGFBP1
Injured nerve	IGF1	MNs	IGFBP2
Injured nerve	IGF1	MNs	IGFBP3
Injured nerve	IGF1	MNs	IGFBP4
Injured nerve	IGF1	MNs	IGFBP5
Injured nerve	IGF1	MNs	IGFBP6
Injured nerve	IGF1	MNs	IGFBP7
Injured nerve	IGF1	MNs	INSR
Injured nerve	IGF2	MNs	IGF1R
Injured nerve	IGF2	MNs	IGF2R
Injured nerve	IGF2	MNs	INSR
Injured nerve	IL15	MNs	IL15RA
Injured nerve	IL15	MNs	IL2RB
Injured nerve	IL15	MNs	IL2RG
Injured nerve	IL16	MNs	CD4
Injured nerve	IL16	MNs	GRIN2A
Injured nerve	IL16	MNs	GRIN2B
Injured nerve	IL16	MNs	GRIN2C
Injured nerve	IL16	MNs	GRIN2D
Injured nerve	IL18	MNs	IL18R1
Injured nerve	IL18	MNs	IL18RAP
Injured nerve	IL1B	MNs	IL1R1
Injured nerve	IL1B	MNs	IL1R2
Injured nerve	IL1B	MNs	IL1RAP
Injured nerve	IL33	MNs	IL1RL1
Injured nerve	IL6	MNs	IL6ST
Injured nerve	INHA	MNs	ACVR2A
Injured nerve	INHA	MNs	TGFBR3
MNs			
Source cell	Ligand	Target cell	Receptor
Injured nerve	INHBA	MNs	ACVR1B
Injured nerve	INHBA	MNs	ACVR2A
Injured nerve	INHBA	MNs	ACVR2B
Injured nerve	INHBB	MNs	ACVR1
Injured nerve	INHBB	MNs	ACVR1B
Injured nerve	INHBB	MNs	ACVR1C
Injured nerve	INHBB	MNs	ACVR2A
Injured nerve	INHBB	MNs	ACVR2B
Injured nerve	JAG1	MNs	NOTCH1
Injured nerve	JAG1	MNs	NOTCH2
Injured nerve	JAG1	MNs	NOTCH3
Injured nerve	JAG2	MNs	NOTCH1
Injured nerve	JAG2	MNs	NOTCH2
Injured nerve	JAG2	MNs	NOTCH3
Injured nerve	LIF	MNs	IL6ST
Injured nerve	LIF	MNs	LIFR
Injured nerve	LTB	MNs	LTBR
Injured nerve	MDK	MNs	ALK
Injured nerve	MDK	MNs	LRP1
Injured nerve	MDK	MNs	PTPRZ1
Injured nerve	MIF	MNs	CD74
Injured nerve	NGF	MNs	NGFR
Injured nerve	NGF	MNs	NTRK1
Injured nerve	NGF	MNs	SORCS3
Injured nerve	NGF	MNs	SORT1
Injured nerve	NOV	MNs	NOTCH1
Injured nerve	NPPC	MNs	NPR2
Injured nerve	NPPC	MNs	NPR3
Injured nerve	NTF3	MNs	NGFR
Injured nerve	NTF3	MNs	NTRK1
Injured nerve	NTF3	MNs	NTRK2
Injured nerve	NTF3	MNs	NTRK3
Injured nerve	NTN1	MNs	DCC
Injured nerve	NTN1	MNs	NEO1
Injured nerve	NTN1	MNs	UNC5B
Injured nerve	NTN1	MNs	UNC5C
Injured nerve	OSM	MNs	IL6ST
Injured nerve	OSM	MNs	LIFR
Injured nerve	OSM	MNs	OSMR
Injured nerve	PDGFA	MNs	PDGFRA
Injured nerve	PDGFB	MNs	PDGFRA
Injured nerve	PDGFB	MNs	PDGFRB
Injured nerve	PDGFC	MNs	PDGFRA
Injured nerve	PF4	MNs	CXCR3
Injured nerve	PF4	MNs	LDLR
Injured nerve	PF4	MNs	THBD
Injured nerve	PGF	MNs	FLT1
Injured nerve	PGF	MNs	NRP1
Injured nerve	PGF	MNs	NRP2
Injured nerve	POMC	MNs	MC1R
Injured nerve	POMC	MNs	MC2R
Injured nerve	POMC	MNs	MC3R
Injured nerve	POMC	MNs	MC5R
Injured nerve	PTHLH	MNs	PTH1R
Injured nerve	PTN	MNs	ALK
Injured nerve	PTN	MNs	PTPRS
Injured nerve	PTN	MNs	PTPRZ1
Injured nerve	RSPO1	MNs	LGR5
Injured nerve	RTN4	MNs	LINGO1
Injured nerve	RTN4	MNs	RTN4R
Injured nerve	RTN4	MNs	RTN4RL1
MNs			
Source cell	Ligand	Target cell	Receptor
Injured nerve	SEMA3B	MNs	PLXNA1
Injured nerve	SEMA3B	MNs	PLXNA2
Injured nerve	SEMA3B	MNs	PLXNA3
Injured nerve	SEMA3B	MNs	PLXNA4
Injured nerve	SEMA3B	MNs	PLXNB2
Injured nerve	SEMA3B	MNs	PLXND1
Injured nerve	SEMA3C	MNs	PLXNA1
Injured nerve	SEMA3C	MNs	PLXNA2
Injured nerve	SEMA3C	MNs	PLXNA3
Injured nerve	SEMA3C	MNs	PLXNA4
Injured nerve	SEMA3C	MNs	PLXNB2
Injured nerve	SEMA3C	MNs	PLXND1
Injured nerve	SEMA3D	MNs	PLXNA1
Injured nerve	SEMA3D	MNs	PLXNA2
Injured nerve	SEMA3D	MNs	PLXNA3
Injured nerve	SEMA3D	MNs	PLXNA4
Injured nerve	SEMA3D	MNs	PLXNB2
Injured nerve	SEMA3D	MNs	PLXND1
Injured nerve	SEMA3E	MNs	PLXNA1
Injured nerve	SEMA3E	MNs	PLXNA2
Injured nerve	SEMA3E	MNs	PLXNA3
Injured nerve	SEMA3E	MNs	PLXNA4
Injured nerve	SEMA3E	MNs	PLXNB2
Injured nerve	SEMA3E	MNs	PLXND1
Injured nerve	SEMA3F	MNs	PLXNA1
Injured nerve	SEMA3F	MNs	PLXNA2
Injured nerve	SEMA3F	MNs	PLXNA3
Injured nerve	SEMA3F	MNs	PLXNA4
Injured nerve	SEMA3F	MNs	PLXNB2
Injured nerve	SEMA3F	MNs	PLXND1
Injured nerve	SEMA3G	MNs	PLXNA1
Injured nerve	SEMA3G	MNs	PLXNA2
Injured nerve	SEMA3G	MNs	PLXNA3
Injured nerve	SEMA3G	MNs	PLXNA4
Injured nerve	SEMA3G	MNs	PLXNB2
Injured nerve	SEMA3G	MNs	PLXND1
Injured nerve	SEMA4A	MNs	PLXNB1
Injured nerve	SEMA4A	MNs	PLXNB2
Injured nerve	SEMA4A	MNs	PLXNC1
Injured nerve	SEMA4A	MNs	PLXND1
Injured nerve	SEMA4B	MNs	PLXNB1
Injured nerve	SEMA4B	MNs	PLXNB2
Injured nerve	SEMA4B	MNs	PLXNC1
Injured nerve	SEMA4C	MNs	PLXNB1
Injured nerve	SEMA4C	MNs	PLXNB2
Injured nerve	SEMA4C	MNs	PLXNC1
Injured nerve	SEMA4D	MNs	PLXNB1
Injured nerve	SEMA4D	MNs	PLXNB2
Injured nerve	SEMA4D	MNs	PLXNC1
Injured nerve	SEMA4F	MNs	PLXNB1
Injured nerve	SEMA4F	MNs	PLXNB2
Injured nerve	SEMA4F	MNs	PLXNC1
Injured nerve	SEMA5A	MNs	PLXNA3
Injured nerve	SEMA5A	MNs	PLXNA4
Injured nerve	SEMA5A	MNs	PLXNC1
Injured nerve	SEMA5B	MNs	PLXNA3
Injured nerve	SEMA5B	MNs	PLXNA4
Injured nerve	SEMA5B	MNs	PLXNC1
Injured nerve	SEMA6A	MNs	PLXNA1
Injured nerve	SEMA6A	MNs	PLXNA2
Injured nerve	SEMA6B	MNs	PLXNA1
MNs			
Source cell	Ligand	Target cell	Receptor
Injured nerve	SEMA6C	MNs	PLXNA1
Injured nerve	SEMA6D	MNs	PLXNA1
Injured nerve	SEMA7A	MNs	PLXNC1
Injured nerve	SHH	MNs	PTCH1
Injured nerve	SHH	MNs	PTCH2
Injured nerve	TGFA	MNs	EGFR
Injured nerve	TGFA	MNs	ERBB2
Injured nerve	TGFB1	MNs	ACVRL1
Injured nerve	TGFB1	MNs	ENG
Injured nerve	TGFB1	MNs	TGFBR1
Injured nerve	TGFB1	MNs	TGFBR2
Injured nerve	TGFB1	MNs	TGFBR3
Injured nerve	TGFB2	MNs	TGFBR1
Injured nerve	TGFB2	MNs	TGFBR2
Injured nerve	TGFB2	MNs	TGFBR3
Injured nerve	TGFB3	MNs	ACVRL1
Injured nerve	TGFB3	MNs	TGFBR1
Injured nerve	TGFB3	MNs	TGFBR2
Injured nerve	TNF	MNs	TNFRSF1A
Injured nerve	TNF	MNs	TNFRSF1B
Injured nerve	TNFSF10	MNs	TNFRSF10B
Injured nerve	TNFSF12	MNs	TNFRSF12A
Injured nerve	TNFSF12	MNs	TNFRSF25
Injured nerve	TNFSF14	MNs	LTBR
Injured nerve	TNFSF14	MNs	TNFRSF14
Injured nerve	TNFSF8	MNs	TNFRSF8
Injured nerve	TNFSF9	MNs	TNFRSF9
Injured nerve	TSLP	MNs	CRLF2
Injured nerve	TSLP	MNs	IL7R
Injured nerve	UCN2	MNs	CRHR2
Injured nerve	VEGFA	MNs	FLT1
Injured nerve	VEGFA	MNs	KDR
Injured nerve	VEGFA	MNs	NRP1
Injured nerve	VEGFA	MNs	NRP2
Injured nerve	VEGFB	MNs	FLT1
Injured nerve	VEGFB	MNs	NRP1
Injured nerve	VEGFC	MNs	FLT4
Injured nerve	VEGFC	MNs	KDR
Injured nerve	VEGFC	MNs	NRP1
Injured nerve	VEGFC	MNs	NRP2
Injured nerve	WNT11	MNs	FZD4
Injured nerve	WNT2	MNs	FZD1
Injured nerve	WNT2	MNs	FZD9
Injured nerve	WNT5A	MNs	FZD2
Injured nerve	WNT5A	MNs	FZD5
Injured nerve	WNT5A	MNs	ROR1
Injured nerve	WNT5A	MNs	ROR2
RGCs			
Source cell	Ligand	Target cell	Receptor
Injured nerve	ADM	RGCs	CALCRL
Injured nerve	ANGPT1	RGCs	TEK
Injured nerve	ANGPT2	RGCs	TEK
Injured nerve	ANGPT4	RGCs	TEK
Injured nerve	APLN	RGCs	APLNR
Injured nerve	ARTN	RGCs	GFRA3
Injured nerve	ARTN	RGCs	RET
Injured nerve	BDNF	RGCs	NGFR
Injured nerve	BDNF	RGCs	NTRK2
Injured nerve	BDNF	RGCs	SORT1
Injured nerve	BMP2	RGCs	BMPR1A
Injured nerve	BMP2	RGCs	BMPR1B
RGCs			
Source cell	Ligand	Target cell	Receptor
Injured nerve	BMP2	RGCs	BMPR2
Injured nerve	BMP2	RGCs	ENG
Injured nerve	BMP2	RGCs	NEO1
Injured nerve	BMP4	RGCs	BMPR1A
Injured nerve	BMP4	RGCs	BMPR1B
Injured nerve	BMP4	RGCs	BMPR2
Injured nerve	BMP4	RGCs	NEO1
Injured nerve	BMP5	RGCs	BMPR1A
Injured nerve	BMP7	RGCs	ACVR1
Injured nerve	BMP7	RGCs	ACVR2A
Injured nerve	BMP7	RGCs	ACVR2B
Injured nerve	BMP7	RGCs	BMPR1A
Injured nerve	BMP7	RGCs	BMPR1B
Injured nerve	BMP7	RGCs	BMPR2
Injured nerve	BMP7	RGCs	NEO1
Injured nerve	BTC	RGCs	EGFR
Injured nerve	BTC	RGCs	ERBB2
Injured nerve	BTC	RGCs	ERBB4
Injured nerve	CCK	RGCs	CCKAR
Injured nerve	CCK	RGCs	CCKBR
Injured nerve	CCL11	RGCs	CCR5
Injured nerve	CCL11	RGCs	CXCR3
Injured nerve	CCL19	RGCs	CCR10
Injured nerve	CCL2	RGCs	CCR1
Injured nerve	CCL2	RGCs	CCR10
Injured nerve	CCL2	RGCs	CCR2
Injured nerve	CCL2	RGCs	DARC
Injured nerve	CCL25	RGCs	CCR10
Injured nerve	CCL25	RGCs	CCR9
Injured nerve	CCL3	RGCs	CCR1
Injured nerve	CCL3	RGCs	CCR4
Injured nerve	CCL3	RGCs	CCR5
Injured nerve	CCL5	RGCs	CCR1
Injured nerve	CCL5	RGCs	CCR4
Injured nerve	CCL5	RGCs	CCR5
Injured nerve	CCL5	RGCs	CXCR3
Injured nerve	CCL5	RGCs	DARC
Injured nerve	CCL5	RGCs	SDC4
Injured nerve	CCL7	RGCs	CCR1
Injured nerve	CCL7	RGCs	CCR10
Injured nerve	CCL7	RGCs	CCR2
Injured nerve	CCL7	RGCs	CCR5
Injured nerve	CCL7	RGCs	CXCR3
Injured nerve	CCL7	RGCs	DARC
Injured nerve	CCL9	RGCs	CCR1
Injured nerve	CLCF1	RGCs	CNTFR
Injured nerve	CLCF1	RGCs	IL6ST
Injured nerve	CLCF1	RGCs	LIFR
Injured nerve	CRLF1	RGCs	CNTFR
Injured nerve	CRLF1	RGCs	IL6ST
Injured nerve	CRLF1	RGCs	LIFR
Injured nerve	CSF1	RGCs	CSF1R
Injured nerve	CX3CL1	RGCs	CX3CR1
Injured nerve	CXCL1	RGCs	CXCR2
Injured nerve	CXCL1	RGCs	DARC
Injured nerve	CXCL10	RGCs	CXCR3
Injured nerve	CXCL12	RGCs	CCR4
Injured nerve	CXCL12	RGCs	CXCR4
Injured nerve	CXCL13	RGCs	CCR10
Injured nerve	CXCL13	RGCs	CXCR3
Injured nerve	CXCL13	RGCs	CXCR5
RGCs			
Source cell	Ligand	Target cell	Receptor
Injured nerve	CXCL16	RGCs	CXCR6
Injured nerve	CXCL2	RGCs	CXCR2
Injured nerve	CXCL9	RGCs	CXCR3
Injured nerve	DHH	RGCs	PTCH1
Injured nerve	DHH	RGCs	PTCH2
Injured nerve	DLL1	RGCs	NOTCH1
Injured nerve	DLL1	RGCs	NOTCH2
Injured nerve	DLL1	RGCs	NOTCH3
Injured nerve	DLL4	RGCs	NOTCH1
Injured nerve	EBI3	RGCs	IL27RA
Injured nerve	EDA	RGCs	EDAR
Injured nerve	EDN3	RGCs	EDNRA
Injured nerve	EDN3	RGCs	EDNRB
Injured nerve	EFNA1	RGCs	EPHA1
Injured nerve	EFNA1	RGCs	EPHA3
Injured nerve	EFNA2	RGCs	EPHA3
Injured nerve	EFNA4	RGCs	EPHA3
Injured nerve	EFNA4	RGCs	EPHA5
Injured nerve	EFNA5	RGCs	EPHA3
Injured nerve	EFNA5	RGCs	EPHA2
Injured nerve	EFNA5	RGCs	EPHA7
Injured nerve	EFNA5	RGCs	EPHA5
Injured nerve	EFNB1	RGCs	EPHB2
Injured nerve	EFNB2	RGCs	EPHB1
Injured nerve	EFNB2	RGCs	EPHA4
Injured nerve	EFNB2	RGCs	EPHA3
Injured nerve	EFNB2	RGCs	EPHB4
Injured nerve	EFNB2	RGCs	EPHB2
Injured nerve	FGF1	RGCs	FGFR1
Injured nerve	FGF1	RGCs	FGFR2
Injured nerve	FGF1	RGCs	FGFR3
Injured nerve	FGF1	RGCs	FGFR4
Injured nerve	FGF10	RGCs	FGFR2
Injured nerve	FGF18	RGCs	FGFR4
Injured nerve	FGF5	RGCs	FGFR1
Injured nerve	FGF5	RGCs	FGFR3
Injured nerve	FGF7	RGCs	FGFR2
Injured nerve	FGF7	RGCs	NRP1
Injured nerve	FIGF	RGCs	FLT4
Injured nerve	FIGF	RGCs	KDR
Injured nerve	FIGF	RGCs	NRP1
Injured nerve	FIGF	RGCs	NRP2
Injured nerve	FSTL1	RGCs	CD14
Injured nerve	FSTL1	RGCs	DIP2A
Injured nerve	GAS6	RGCs	AXL
Injured nerve	GDF11	RGCs	ACVR1B
Injured nerve	GDF11	RGCs	ACVR1C
Injured nerve	GDF11	RGCs	ACVR2B
Injured nerve	GDNF	RGCs	GFRA1
Injured nerve	GDNF	RGCs	GFRA2
Injured nerve	GDNF	RGCs	RET
Injured nerve	HBEGF	RGCs	EGFR
Injured nerve	HBEGF	RGCs	ERBB4
Injured nerve	HGF	RGCs	MET
Injured nerve	IGF1	RGCs	IGF1R
Injured nerve	IGF1	RGCs	IGFBP1
Injured nerve	IGF1	RGCs	IGFBP2
Injured nerve	IGF1	RGCs	IGFBP3
Injured nerve	IGF1	RGCs	IGFBP4
Injured nerve	IGF1	RGCs	IGFBP5
Injured nerve	IGF1	RGCs	IGFBP6
RGCs			
Source cell	Ligand	Target cell	Receptor
Injured nerve	IGF1	RGCs	IGFBP7
Injured nerve	IGF1	RGCs	INSR
Injured nerve	IGF2	RGCs	IGF1R
Injured nerve	IGF2	RGCs	IGF2R
Injured nerve	IGF2	RGCs	INSR
Injured nerve	IL15	RGCs	IL15RA
Injured nerve	IL15	RGCs	IL2RB
Injured nerve	IL15	RGCs	IL2RG
Injured nerve	IL16	RGCs	CD4
Injured nerve	IL16	RGCs	GRIN2A
Injured nerve	IL16	RGCs	GRIN2B
Injured nerve	IL16	RGCs	GRIN2C
Injured nerve	IL16	RGCs	GRIN2D
Injured nerve	IL18	RGCs	IL18RAP
Injured nerve	IL1B	RGCs	IL1R1
Injured nerve	IL1B	RGCs	IL1RAP
Injured nerve	IL33	RGCs	IL1RL1
Injured nerve	IL6	RGCs	IL6ST
Injured nerve	INHA	RGCs	ACVR2A
Injured nerve	INHA	RGCs	TGFBR3
Injured nerve	INHBA	RGCs	ACVR1B
Injured nerve	INHBA	RGCs	ACVR2A
Injured nerve	INHBA	RGCs	ACVR2B
Injured nerve	INHBB	RGCs	ACVR1
Injured nerve	INHBB	RGCs	ACVR1B
Injured nerve	INHBB	RGCs	ACVR1C
Injured nerve	INHBB	RGCs	ACVR2A
Injured nerve	INHBB	RGCs	ACVR2B
Injured nerve	JAG1	RGCs	NOTCH1
Injured nerve	JAG1	RGCs	NOTCH2
Injured nerve	JAG1	RGCs	NOTCH3
Injured nerve	JAG2	RGCs	NOTCH1
Injured nerve	JAG2	RGCs	NOTCH2
Injured nerve	JAG2	RGCs	NOTCH3
Injured nerve	LIF	RGCs	IL6ST
Injured nerve	LIF	RGCs	LIFR
Injured nerve	LTB	RGCs	LTBR
Injured nerve	MDK	RGCs	ALK
Injured nerve	MDK	RGCs	LRP1
Injured nerve	MDK	RGCs	LRP2
Injured nerve	MDK	RGCs	PTPRZ1
Injured nerve	MIF	RGCs	CD74
Injured nerve	NGF	RGCs	NGFR
Injured nerve	NGF	RGCs	NTRK1
Injured nerve	NGF	RGCs	SORCS3
Injured nerve	NGF	RGCs	SORT1
Injured nerve	NOV	RGCs	NOTCH1
Injured nerve	NPPC	RGCs	NPR2
Injured nerve	NPPC	RGCs	NPR3
Injured nerve	NTF3	RGCs	NGFR
Injured nerve	NTF3	RGCs	NTRK1
Injured nerve	NTF3	RGCs	NTRK2
Injured nerve	NTF3	RGCs	NTRK3
Injured nerve	NTN1	RGCs	DCC
Injured nerve	NTN1	RGCs	NEO1
Injured nerve	NTN1	RGCs	UNC5B
Injured nerve	NTN1	RGCs	UNC5C
Injured nerve	OSM	RGCs	IL6ST
Injured nerve	OSM	RGCs	LIFR
Injured nerve	OSM	RGCs	OSMR
Injured nerve	PDGFA	RGCs	PDGFRA
RGCs			
Source cell	Ligand	Target cell	Receptor
Injured nerve	PDGFB	RGCs	PDGFRA
Injured nerve	PDGFB	RGCs	PDGFRB
Injured nerve	PDGFC	RGCs	PDGFRA
Injured nerve	PF4	RGCs	CXCR3
Injured nerve	PF4	RGCs	DARC
Injured nerve	PF4	RGCs	LDLR
Injured nerve	PF4	RGCs	THBD
Injured nerve	PGF	RGCs	FLT1
Injured nerve	PGF	RGCs	NRP1
Injured nerve	PGF	RGCs	NRP2
Injured nerve	POMC	RGCs	MC1R
Injured nerve	POMC	RGCs	MC3R
Injured nerve	POMC	RGCs	MC4R
Injured nerve	POMC	RGCs	MC5R
Injured nerve	PTHLH	RGCs	PTH1R
Injured nerve	PTN	RGCs	ALK
Injured nerve	PTN	RGCs	PTPRS
Injured nerve	PTN	RGCs	PTPRZ1
Injured nerve	RSPO1	RGCs	LGR5
Injured nerve	RTN4	RGCs	LINGO1
Injured nerve	RTN4	RGCs	RTN4R
Injured nerve	RTN4	RGCs	RTN4RL1
Injured nerve	SEMA3B	RGCs	PLXNA1
Injured nerve	SEMA3B	RGCs	PLXNA2
Injured nerve	SEMA3B	RGCs	PLXNA3
Injured nerve	SEMA3B	RGCs	PLXNA4
Injured nerve	SEMA3B	RGCs	PLXNB2
Injured nerve	SEMA3B	RGCs	PLXND1
Injured nerve	SEMA3C	RGCs	PLXNA1
Injured nerve	SEMA3C	RGCs	PLXNA2
Injured nerve	SEMA3C	RGCs	PLXNA3
Injured nerve	SEMA3C	RGCs	PLXNA4
Injured nerve	SEMA3C	RGCs	PLXNB2
Injured nerve	SEMA3C	RGCs	PLXND1
Injured nerve	SEMA3D	RGCs	PLXNA1
Injured nerve	SEMA3D	RGCs	PLXNA2
Injured nerve	SEMA3D	RGCs	PLXNA3
Injured nerve	SEMA3D	RGCs	PLXNA4
Injured nerve	SEMA3D	RGCs	PLXNB2
Injured nerve	SEMA3D	RGCs	PLXND1
Injured nerve	SEMA3E	RGCs	PLXNA1
Injured nerve	SEMA3E	RGCs	PLXNA2
Injured nerve	SEMA3E	RGCs	PLXNA3
Injured nerve	SEMA3E	RGCs	PLXNA4
Injured nerve	SEMA3E	RGCs	PLXNB2
Injured nerve	SEMA3E	RGCs	PLXND1
Injured nerve	SEMA3F	RGCs	PLXNA1
Injured nerve	SEMA3F	RGCs	PLXNA2
Injured nerve	SEMA3F	RGCs	PLXNA3
Injured nerve	SEMA3F	RGCs	PLXNA4
Injured nerve	SEMA3F	RGCs	PLXNB2
Injured nerve	SEMA3F	RGCs	PLXND1
Injured nerve	SEMA3G	RGCs	PLXNA1
Injured nerve	SEMA3G	RGCs	PLXNA2
Injured nerve	SEMA3G	RGCs	PLXNA3
Injured nerve	SEMA3G	RGCs	PLXNA4
Injured nerve	SEMA3G	RGCs	PLXNB2
Injured nerve	SEMA3G	RGCs	PLXND1
Injured nerve	SEMA4A	RGCs	PLXNB1
Injured nerve	SEMA4A	RGCs	PLXNB2
Injured nerve	SEMA4A	RGCs	PLXNC1
RGCs			
Source cell	Ligand	Target cell	Receptor
Injured nerve	SEMA4A	RGCs	PLXND1
Injured nerve	SEMA4B	RGCs	PLXNB1
Injured nerve	SEMA4B	RGCs	PLXNB2
Injured nerve	SEMA4B	RGCs	PLXNC1
Injured nerve	SEMA4C	RGCs	PLXNB1
Injured nerve	SEMA4C	RGCs	PLXNB2
Injured nerve	SEMA4C	RGCs	PLXNC1
Injured nerve	SEMA4D	RGCs	PLXNB1
Injured nerve	SEMA4D	RGCs	PLXNB2
Injured nerve	SEMA4D	RGCs	PLXNC1
Injured nerve	SEMA4F	RGCs	PLXNB1
Injured nerve	SEMA4F	RGCs	PLXNB2
Injured nerve	SEMA4F	RGCs	PLXNC1
Injured nerve	SEMA5A	RGCs	PLXNA3
Injured nerve	SEMA5A	RGCs	PLXNA4
Injured nerve	SEMA5A	RGCs	PLXNC1
Injured nerve	SEMA5B	RGCs	PLXNA3
Injured nerve	SEMA5B	RGCs	PLXNA4
Injured nerve	SEMA5B	RGCs	PLXNC1
Injured nerve	SEMA6A	RGCs	PLXNA1
Injured nerve	SEMA6A	RGCs	PLXNA2
Injured nerve	SEMA6B	RGCs	PLXNA1
Injured nerve	SEMA6C	RGCs	PLXNA1
Injured nerve	SEMA6D	RGCs	PLXNA1
Injured nerve	SEMA7A	RGCs	PLXNC1
Injured nerve	SHH	RGCs	PTCH1
Injured nerve	SHH	RGCs	PTCH2
Injured nerve	TGFA	RGCs	EGFR
Injured nerve	TGFA	RGCs	ERBB2
Injured nerve	TGFB1	RGCs	ACVRL1
Injured nerve	TGFB1	RGCs	ENG
Injured nerve	TGFB1	RGCs	TGFBR1
Injured nerve	TGFB1	RGCs	TGFBR2
Injured nerve	TGFB1	RGCs	TGFBR3
Injured nerve	TGFB2	RGCs	TGFBR1
Injured nerve	TGFB2	RGCs	TGFBR2
Injured nerve	TGFB2	RGCs	TGFBR3
Injured nerve	TGFB3	RGCs	ACVRL1
Injured nerve	TGFB3	RGCs	TGFBR1
Injured nerve	TGFB3	RGCs	TGFBR2
Injured nerve	TNF	RGCs	TNFRSF1A
Injured nerve	TNF	RGCs	TNFRSF1B
Injured nerve	TNFSF10	RGCs	TNFRSF10B
Injured nerve	TNFSF12	RGCs	TNFRSF12A
Injured nerve	TNFSF12	RGCs	TNFRSF25
Injured nerve	TNFSF14	RGCs	LTBR
Injured nerve	TNFSF14	RGCs	TNFRSF14
Injured nerve	TNFSF8	RGCs	TNFRSF8
Injured nerve	TNFSF9	RGCs	TNFRSF9
Injured nerve	TSLP	RGCs	CRLF2
Injured nerve	TSLP	RGCs	IL7R
Injured nerve	UCN2	RGCs	CRHR2
Injured nerve	VEGFA	RGCs	FLT1
Injured nerve	VEGFA	RGCs	KDR
Injured nerve	VEGFA	RGCs	NRP1
Injured nerve	VEGFA	RGCs	NRP2
Injured nerve	VEGFB	RGCs	FLT1
Injured nerve	VEGFB	RGCs	NRP1
Injured nerve	VEGFC	RGCs	FLT4
Injured nerve	VEGFC	RGCs	KDR
Injured nerve	VEGFC	RGCs	NRP1
RGCs			
Source cell	Ligand	Target cell	Receptor
Injured nerve	VEGFC	RGCs	NRP2
Injured nerve	WNT11	RGCs	FZD4
Injured nerve	WNT2	RGCs	FZD1
Injured nerve	WNT2	RGCs	FZD9
Injured nerve	WNT5A	RGCs	FZD2
Injured nerve	WNT5A	RGCs	FZD5
Injured nerve	WNT5A	RGCs	ROR1
Injured nerve	WNT5A	RGCs	ROR2

**Figure 6.**
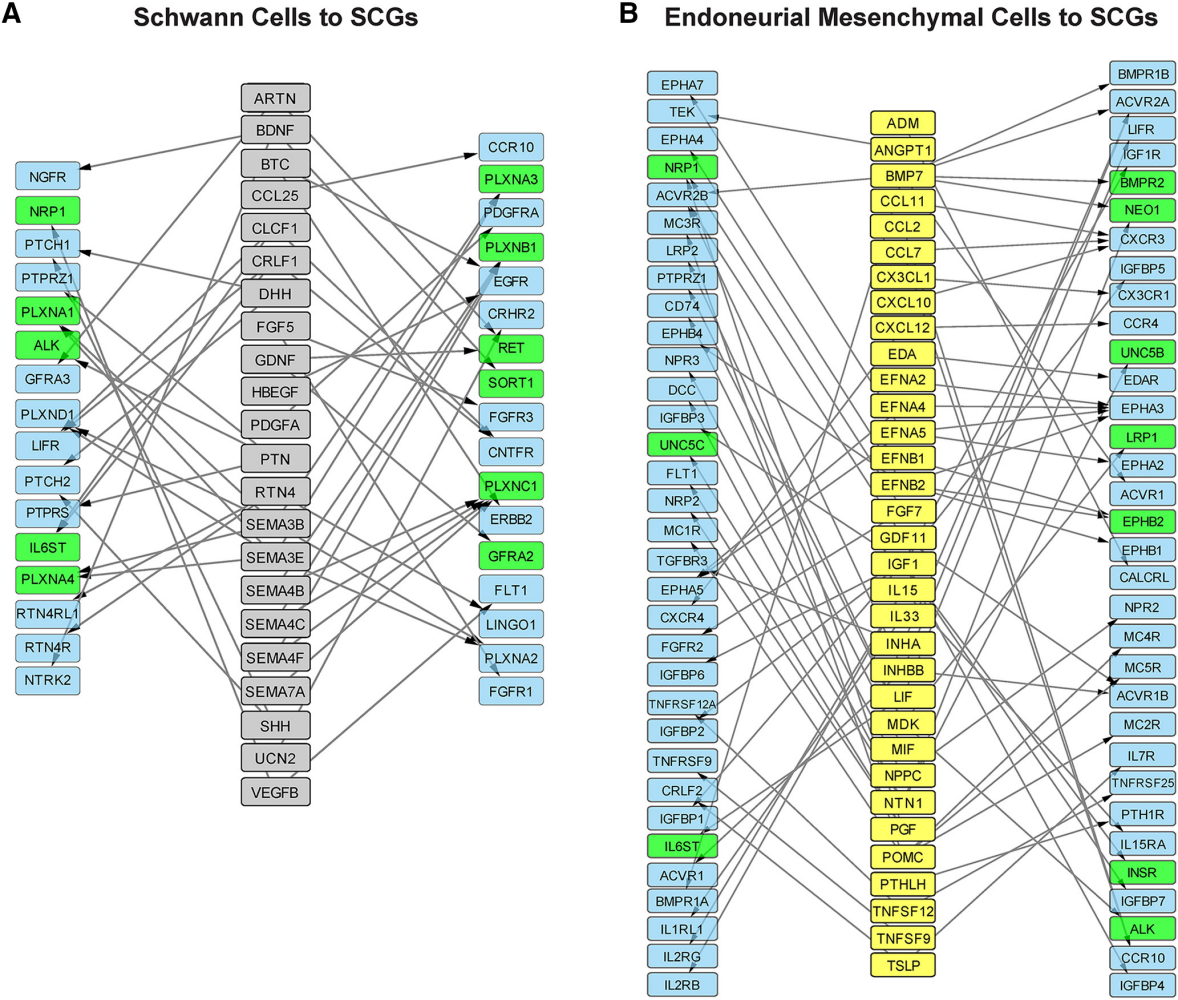
Predicted unidirectional ligand-receptor interactions between injured sciatic nerve Schwann cells or endoneurial mesenchymal cells and sympathetic neurons (see also Extended Data Fig. 6-1). Models showing predicted unidirectional interactions between the ligands most highly expressed by injured nerve Schwann cells (***A***) or endoneurial mesenchymal cells (***B***) and their receptors on cultured sympathetic neurons (SCGs). Ligands are shown in the central columns in ***A***, ***B*** and are color coded as in Figure 5 (Schwann cell ligands in gray and endoneurial mesenchymal cell ligands in yellow). Receptors are shown on either side of the ligand column and also include coreceptors that are well-characterized components of receptor complexes. Receptors that were observed at both the transcriptomic and proteomic levels are colored green while those defined only at the transcriptomic level are colored blue. Arrows indicate directionality of interactions. Note that many ligands interact with multiple receptors and, conversely, that multiple ligands are sometimes predicted to share receptors.

**Figure 7.**
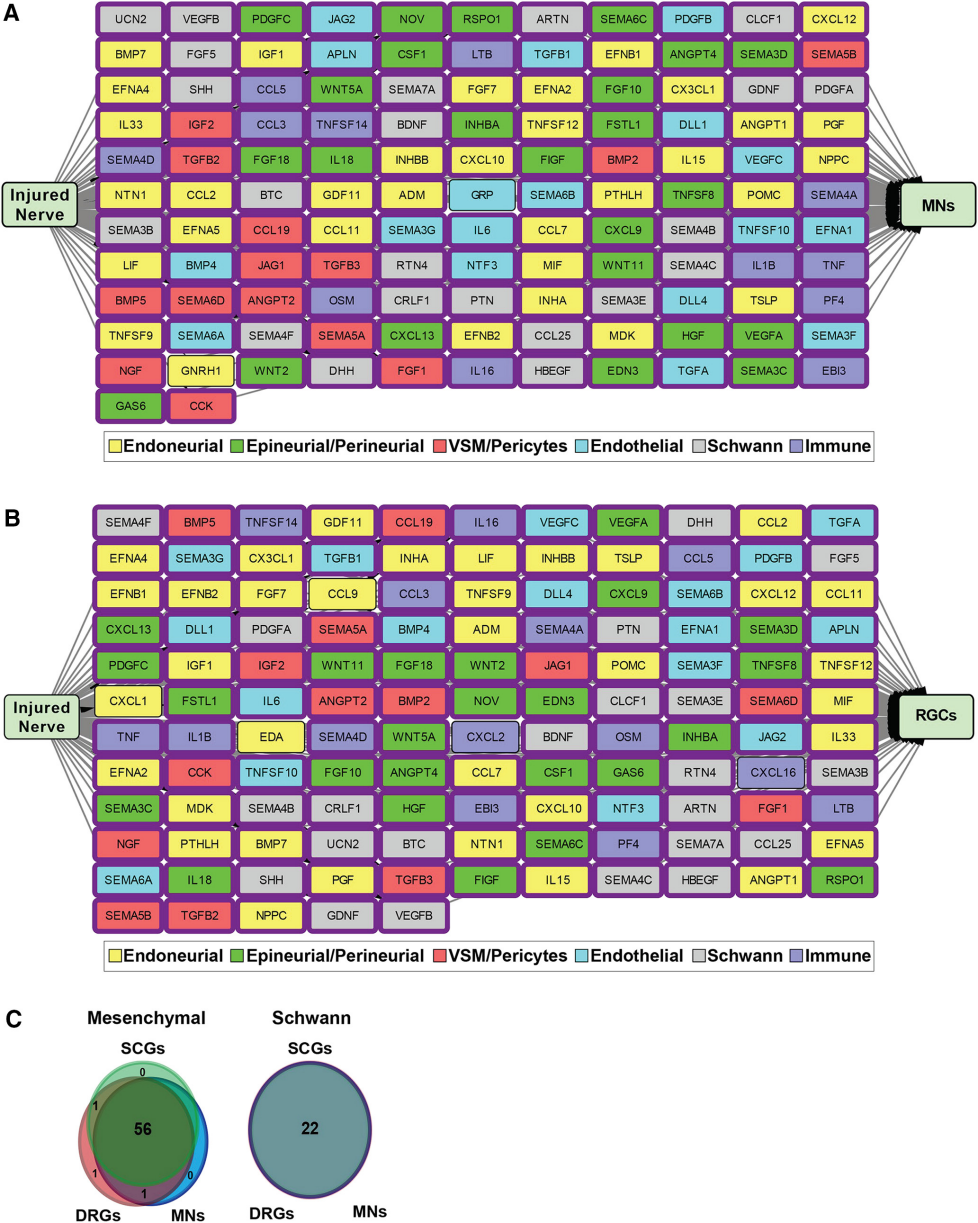
Modeling the potential paracrine interactions between ligands of the injured sciatic nerve and receptors on motor neurons and RGCs. ***A***, ***B***, Models showing predicted unidirectional paracrine interaction networks between the 143 injured nerve ligands and receptors expressed by motor neurons (***A***, MNs) and RGCs (***B***), as defined at the transcriptional level. Interactions were predicted by computational modeling using the ligand-receptor database, and then manually curated for well-validated interactions. Nodes represent ligands that are color coded to identify the injured nerve cell type with the highest expression of the ligand mRNA based on the scRNA-seq analysis. Arrows indicate directionality of interactions. Predicted interactions that were shared among all four injured nerve-neuron models are indicated by a purple box around the corresponding ligand nodes. ***C***, Venn diagrams showing the overlap of predicted ligand-receptor interactions between injured nerve mesenchymal cells (left) or Schwann cells (right) and sympathetic neurons (SCGs), sensory neurons (DRGs), and motor neurons (MNs).

Predicted unidirectional ligand-receptor interactions between the injured sciatic nerve ligands and receptors on sympathetic (SCGs) neurons, sensory (DRGs) neurons, motor neurons (MNs), and retinal ganglion cells (RGCs). Directionality of predicted paracrine interactions are indicated by the source cell (ligand) and target cell (receptor) column designations. Some ligands are predicted to act on more than one receptor. The interactions shown here accompany the models presented in Figures 5-7 and Extended Data Figure 6-1.

10.1523/ENEURO.0066-20.2020.f6-1 Extended Data Figure 6-1 Predicted unidirectional ligand-receptor interactions between injured sciatic nerve Schwann cells or endoneurial mesenchymal cells and sensory neurons. Models showing predicted unidirectional interactions between the ligands most highly expressed by injured nerve Schwann cells (***A***) or endoneurial mesenchymal cells (***B***) and their receptors on cultured sensory neurons (DRGs). Ligands are shown in the central columns in ***A***, ***B*** and are color coded as in Figure 5 (Schwann cell ligands in grey and endoneurial mesenchymal cell ligands in yellow). Receptors are shown on either side of the ligand column and also include coreceptors that are well-characterized components of receptor complexes. Receptors that were observed at both the transcriptomic and proteomic levels are colored green while those defined only at the transcriptomic level are colored blue. Arrows indicate directionality of interactions. Note that many ligands interact with multiple receptors and, conversely, that multiple ligands are sometimes predicted to share receptors. Download Figure 6-1, TIF file.

We then used the scRNA-seq data to define the nerve cell types that made the ligands involved in the predicted interactions (Figs. 5*C*,*D*, 6*A*,*B*; Extended Data Fig. 6-1*A*,*B*). Almost half (57) of 122 shared predicted sympathetic and sensory neuron interactions involved ligands that were expressed in the highest proportions in *Pdgfra*-positive mesenchymal cells including FGF10, FGF18, HGF, SEMA3D, BMP7, IL33, and PTHLH (asterisks in the models denote ligands made by 4-fold more of the indicated cell relative to all other cell types). Moreover, 33 of these were highest in the endoneurial mesenchymal cells. Another 22 ligands were highest in Schwann cells, including ARTN, BTC, DHH, FGF5, GDNF, SEMA3B, SHH, and UCN2. The remaining 43 predicted interactions were split almost equally between endothelial cells (17), VSM/pericyte cells (14), and immune cells (12), and included well-characterized nerve ligands such as NGF, NT3 (*Ntf3*), and IGF2. Notably, while some of these ligands were predicted to signal via dedicated receptors (Fig. 6*A*,*B*; Extended Data Fig. 6-1*A*,*B*; Table 9), many others shared receptors or coreceptors, suggesting the potential for convergent signaling. As examples, CLCF1, CRLF1, and LIF were all predicted to share the receptor components LIFR and gp130, and ANGPT1, ANGPT2, and ANGPT4 were all predicted to act by interacting with TEK.

### Modeling predicts that many nerve ligands have the capacity to act on both PNS and CNS neurons

This analysis predicts that peripheral nerve cells produce ligands that could act on at least two populations of peripheral neurons. To test the idea that this might reflect a ligand environment that is generally supportive of axonal growth, we asked about motor neurons, which also project axons via the sciatic nerve, and RGCs, CNS neurons that normally do not regenerate in the CNS, but will regenerate into peripheral nerve grafts (Politis and Spencer, 1986; for review, see Benowitz et al., 2017).

For motor neurons, we analyzed previously published microarray data from microdissected P7 mouse lumbar motor neurons (Kaplan et al., 2014), identifying receptor mRNAs using the same thresholding cutoff as for the sensory neurons (top 87% of mRNAs). Of 322 receptor mRNAs defined in the motor neuron dataset using this approach, 272 were also expressed by sensory and sympathetic neurons (Table 10). Computational modeling with the motor neuron receptors and the 143 injured nerve ligands showed that of the 122 shared sympathetic and sensory neuron interactions, 121 were also predicted for motor neurons, with the endoneurial ligand EDA the only exception (Fig. 7*A*,*C*; Table 9). There were also two predicted nerve to motor neuron interactions involving GRP-GRPR and GNRH1-GNRHR that were not shared with both sympathetic and sensory neurons.

**Table 10:** Receptors identified on motor neurons and RGCs using microarrays and RNA-seq

MNs (322)	DRGs. SCGs,and MNs (272)	RGCs (320)	DRGs, SCGs,MNs, andRGCs (258)
Acvr1	Acvr1	Acvr1	Acvr1
Acvr1b	Acvr1b	Acvr1b	Acvr1b
Acvr1c	Acvr2a	Acvr1c	Acvr2a
Acvr2a	Acvr2b	Acvr2a	Acvr2b
Acvr2b	Acvrl1	Acvr2b	Acvrl1
Acvrl1	Adcyap1r1	Acvrl1	Adcyap1r1
Adcyap1r1	Adipor1	Adcyap1r1	Adipor1
Adipor1	Adipor2	Adipor1	Adipor2
Adipor2	Adora2a	Adipor2	Adora2a
Adora2a	Adra1b	Adora2a	Adra1b
Adra1a	Adrb2	Adra1a	Adrb2
Adra1b	Ager	Adra1b	Ager
Adrb2	Alk	Adrb2	Alk
Ager	Amfr	Ager	Amfr
Alk	Aplnr	Alk	Aplnr
Amfr	Avpr1a	Amfr	Avpr1a
Amhr2	Axl	Amhr2	Axl
Aplnr	Bdkrb2	Aplnr	Bdkrb2
Ar	Bmpr1a	Ar	Bmpr1a
Avpr1a	Bmpr1b	Avpr1a	Bmpr1b
Avpr1b	Bmpr2	Avpr2	Bmpr2
Avpr2	C3ar1	Axl	C3ar1
Axl	C5ar1	Bdkrb2	C5ar1
Bdkrb2	Calcrl	Bmpr1a	Calcrl
Bmpr1a	Cckar	Bmpr1b	Cckar
Bmpr1b	Cckbr	Bmpr2	Cckbr
Bmpr2	Ccr10	Btn1a1	Ccr10
Btn1a1	Cd14	C3ar1	Cd14
C3ar1	Cd4	C5ar1	Cd4
C5ar1	Cd44	Calcr	Cd44
Calcr	Cd5l	Calcrl	Cd5l
Calcrl	Cd7	Cckar	Cd74
Cckar	Cd74	Cckbr	Cntfr
Cckbr	Cntfr	Ccr1	Crhr1
Ccr1	Crhr1	Ccr10	Crhr2
Ccr10	Crhr2	Ccr2	Crlf1
Ccr2	Crlf1	Ccr4	Crlf2
Ccr3	Crlf2	Ccr5	Csf1r
Ccr4	Csf1r	Ccr9	Csf2ra
Ccr5	Csf2ra	Cd14	Csf2rb
Ccr6	Csf2rb	Cd27	Csf3r
Ccr7	Csf3r	Cd33	Ctf1
Ccr8	Ctf1	Cd4	Cx3cr1
Ccr9	Cx3cr1	Cd40	Cxcr3
Cd14	Cxcr3	Cd44	Cxcr4
Cd27	Cxcr4	Cd5l	Dcc
Cd33	Dcc	Cd74	Ddr1
Cd4	Ddr1	Cntfr	Derl1
Cd40	Derl1	Cr2	Dip2a
Cd44	Dip2a	Crhr1	Ednra
Cd5l	Ednra	Crhr2	Ednrb
Cd7	Ednrb	Crlf1	Egfr
Cd74	Egfr	Crlf2	Eng
Cntfr	Eng	Csf1r	Epha1
Cr2	Epha1	Csf2ra	Epha2
Crhr1	Epha2	Csf2rb	Epha3
Crhr2	Epha3	Csf3r	Epha4
Crlf1	Epha4	Ctf1	Epha5
Crlf2	Epha5	Cx3cr1	Epha7
Csf1r	Epha7	Cxcr2	Ephb1
Csf2ra	Ephb1	Cxcr3	Ephb2
Csf2rb	Ephb2	Cxcr4	Ephb3
Csf3r	Ephb3	Cxcr5	Ephb4
Ctf1	Ephb4	Cxcr6	Epor
Cx3cr1	Epor	Darc	Eps15l1
Cxcr2	Eps15l1	Dcc	Erbb2
Cxcr3	Erbb2	Ddr1	Erbb3
Cxcr4	Erbb3	Derl1	Esr2
Cxcr5	Esr2	Dip2a	F2r
Cxcr6	F2r	Dpp4	Fgfr1
Dcc	Fas	Edar	Fgfr2
Ddr1	Fgfr1	Ednra	Fgfr3
Derl1	Fgfr2	Ednrb	Fgfr4
Dip2a	Fgfr3	Egfr	Fgfrl1
Dpp4	Fgfr4	Eng	Flt1
Edar	Fgfrl1	Epha1	Flt3
Ednra	Flt1	Epha2	Flt4
Ednrb	Flt3	Epha3	Folr1
Egfr	Flt4	Epha4	Fzd1
Eng	Folr1	Epha5	Fzd2
Epha1	Fshr	Epha7	Fzd4
Epha2	Fzd1	Ephb1	Fzd5
Epha3	Fzd2	Ephb2	Fzd9
Epha5	Fzd4	Ephb3	Gabbr1
Ephb1	Fzd5	Ephb4	Galr2
Ephb2	Fzd9	Epor	Gcgr
Ephb3	Gabbr1	Eps15l1	Gfra2
Epor	Galr2	Erbb2	Gfra3
Eps15l1	Gcgr	Erbb3	Gfra4
Erbb2	Gfra2	Erbb4	Ghr
Erbb3	Gfra3	Esr1	Gipr
Erbb4	Gfra4	Esr2	Gosr1
Esr1	Ghr	F2r	Grik5
Esr2	Ghrhr	F2rl1	Grin2a
F2r	Gipr	F2rl3	Grin2b
F2rl1	Glp1r	Fgfr1	Grin2c
F2rl2	Gosr1	Fgfr2	Grin2d
F2rl3	Grik5	Fgfr3	Gucy2c
Fas	Grin2a	Fgfr4	Hcrtr1
Fgfr1	Grin2b	Fgfrl1	Hcrtr2
Fgfr2	Grin2c	Flt1	Hnf4a
Fgfr3	Grin2d	Flt3	Hpn
Fgfr4	Gucy2c	Flt4	Ifnar1
Fgfrl1	Hcrtr1	Folr1	Ifnar2
Flt1	Hcrtr2	Fzd1	Ifngr1
Flt3	Hnf4a	Fzd2	Ifngr2
Flt4	Hpn	Fzd4	Igf1r
Folr1	Ifnar1	Fzd5	Igf2r
Fshr	Ifnar2	Fzd8	Igfbp1
Fzd1	Ifngr1	Fzd9	Igfbp2
Fzd2	Ifngr2	Gabbr1	Igfbp3
Fzd4	Igf1r	Galr1	Igfbp4
Fzd5	Igf2r	Galr2	Igfbp5
Fzd8	Igfbp1	Gcgr	Igfbp6
Fzd9	Igfbp2	Gfra1	Igfbp7
Gabbr1	Igfbp3	Gfra2	Il10ra
Galr1	Igfbp4	Gfra3	Il10rb
Galr2	Igfbp5	Gfra4	Il12rb1
Gcgr	Igfbp6	Ghr	Il15ra
Gfra1	Igfbp7	Ghsr	Il17ra
Gfra2	Il10ra	Gipr	Il17rc
Gfra3	Il10rb	Glp2r	Il18rap
Gfra4	Il12rb1	Gosr1	Il1r1
Ghr	Il15ra	Gpr151	Il1rap
Ghrhr	Il17ra	Grik5	Il1rl1
Ghsr	Il17rc	Grin2a	Il1rl2
Gipr	Il18r1	Grin2b	Il21r
Glp1r	Il18rap	Grin2c	Il27ra
Gnrhr	Il1r1	Grin2d	Il2rb
Gosr1	Il1r2	Gucy2c	Il2rg
Gpr151	Il1rap	Hcrtr1	Il3ra
Grik5	Il1rl1	Hcrtr2	Il6st
Grin2a	Il1rl2	Hnf4a	Il7r
Grin2b	Il21r	Hpn	Insr
Grin2c	Il22ra1	Ifnar1	Irs1
Grin2d	Il27ra	Ifnar2	Itga2
Grpr	Il2ra	Ifngr1	Itga2b
Gucy2c	Il2rb	Ifngr2	Itga5
Hcrtr1	Il2rg	Igf1r	Itga9
Hcrtr2	Il3ra	Igf2r	Itgal
Hnf4a	Il6st	Igfbp1	Itgav
Hpn	Il7r	Igfbp2	Itgb1
Hrh4	Insr	Igfbp3	Itgb3
Ifnar1	Irs1	Igfbp4	Itgb5
Ifnar2	Itga2	Igfbp5	Itgb6
Ifngr1	Itga2b	Igfbp6	Itgb8
Ifngr2	Itga5	Igfbp7	Itpr3
Igf1r	Itga9	Il10ra	Kit
Igf2r	Itgal	Il10rb	Ldlr
Igfbp1	Itgav	Il12rb1	Lgals3bp
Igfbp2	Itgb1	Il12rb2	Lgr5
Igfbp3	Itgb3	Il13ra1	Lifr
Igfbp4	Itgb5	Il15ra	Lingo1
Igfbp5	Itgb6	Il17ra	Lrp1
Igfbp6	Itgb8	Il17rb	Lrp5
Igfbp7	Itpr3	Il17rc	Lrp6
Il10ra	Kit	Il18rap	Lsr
Il10rb	Ldlr	Il1r1	Ltbr
Il12rb1	Lgals3bp	Il1rap	Mc1r
Il12rb2	Lgr5	Il1rl1	Mc3r
Il13ra1	Lifr	Il1rl2	Mc5r
Il13ra2	Lingo1	Il20ra	Mchr1
Il15ra	Lrp1	Il20rb	Met
Il17ra	Lrp5	Il21r	Mst1r
Il17rb	Lrp6	Il27ra	Ncoa3
Il17rc	Lsr	Il2rb	Ncor1
Il18r1	Ltbr	Il2rg	Neo1
Il18rap	Marco	Il3ra	Ngfr
Il1r1	Mc1r	Il6st	Notch1
Il1r2	Mc2r	Il7r	Notch2
Il1rap	Mc3r	Il9r	Notch3
Il1rl1	Mc5r	Insr	Npr1
Il1rl2	Mchr1	Irs1	Npr2
Il20ra	Met	Itga2	Npr3
Il20rb	Mpl	Itga2b	Npy1r
Il21r	Mst1r	Itga5	Npy5r
Il22ra1	Ncoa3	Itga9	Nr3c1
Il22ra2	Ncor1	Itgal	Nrp1
Il27ra	Neo1	Itgav	Nrp2
Il2ra	Ngfr	Itgb1	Ntng1
Il2rb	Notch1	Itgb2	Ntng2
Il2rg	Notch2	Itgb3	Ntrk1
Il3ra	Notch3	Itgb5	Ntrk2
Il5ra	Npffr2	Itgb6	Ntrk3
Il6st	Npr1	Itgb8	Ntsr1
Il7r	Npr2	Itpr3	Oprl1
Il9r	Npr3	Kdr	Osmr
Insr	Npy1r	Kit	Oxtr
Irs1	Npy2r	Ldlr	Pdgfa
Itga2	Npy5r	Lepr	Pdgfra
Itga2b	Nr3c1	Lgals3bp	Pdgfrb
Itga5	Nrp1	Lgr5	Pgr
Itga9	Nrp2	Lhcgr	Plaur
Itgal	Ntng1	Lifr	Plgrkt
Itgav	Ntng2	Lingo1	Plxna1
Itgb1	Ntrk1	Loxl2	Plxna2
Itgb2	Ntrk2	Lrp1	Plxna3
Itgb3	Ntrk3	Lrp2	Plxna4
Itgb5	Ntsr1	Lrp5	Plxnb1
Itgb6	Oprl1	Lrp6	Plxnc1
Itgb8	Osmr	Lsr	Plxnd1
Itpr3	Oxtr	Ltbr	Procr
Kdr	Pdgfa	Mc1r	Prokr1
Kit	Pdgfra	Mc3r	Prokr2
Ldlr	Pdgfrb	Mc4r	Ptch1
Lepr	Pgr	Mc5r	Ptch2
Lgals3bp	Plaur	Mchr1	Pth1r
Lgr5	Plgrkt	Met	Ptprk
Lhcgr	Plxna1	Mst1r	Ptprs
Lifr	Plxna2	Ncoa3	Ptprz1
Lrp1	Plxna3	Ncor1	Ret
Lrp2	Plxna4	Neo1	Robo3
Lrp5	Plxnb1	Ngfr	Ror1
Lrp6	Plxnc1	Nmbr	Ror2
Lsr	Plxnd1	Nmur2	Rorb
Ltbr	Procr	Notch1	Rtn4r
Marco	Prokr1	Notch2	Rtn4rl1
Mc1r	Prokr2	Notch3	Rxrg
Mc2r	Ptch1	Npffr1	Ryr1
Mc3r	Ptch2	Npr1	Ryr2
Mc5r	Pth1r	Npr2	Sctr
Mchr1	Ptprk	Npr3	Sdc4
Met	Ptprs	Npy1r	Sfrp1
Mpl	Ptprz1	Npy5r	Sfrp2
Ncoa3	Ret	Nr3c1	Slc1a5
Ncor1	Robo3	Nrp1	Sorcs3
Ngfr	Ror1	Nrp2	Sort1
Nmbr	Ror2	Ntng1	Sstr1
Nmur2	Rorb	Ntng2	Sstr2
Notch1	Rtn4r	Ntrk1	Sstr3
Notch2	Rtn4rl1	Ntrk2	Sstr4
Notch3	Rxrg	Ntrk3	Sstr5
Npffr2	Ryr1	Ntsr1	Tek
Npr1	Ryr2	Oprl1	Tgfbr2
Npr2	Sctr	Osmr	Tgfbr3
Npr3	Sdc4	Oxtr	Thbd
Npy1r	Sfrp1	Pdgfa	Thra
Npy2r	Sfrp2	Pdgfra	Thrap3
Npy5r	Slc1a5	Pdgfrb	Tnfrsf10b
Nr3c1	Sorcs3	Pgr	Tnfrsf11a
Nrp1	Sort1	Plat	Tnfrsf11b
Nrp2	Sstr1	Plaur	Tnfrsf12a
Ntrk1	Sstr2	Plgrkt	Tnfrsf13c
Ntrk2	Sstr3	Plxna1	Tnfrsf14
Ntrk3	Sstr4	Plxna2	Tnfrsf17
Ntsr1	Sstr5	Plxna3	Tnfrsf18
Oprl1	Tek	Plxna4	Tnfrsf1a
Osmr	Tgfbr2	Plxnb1	Tnfrsf1b
Oxtr	Tgfbr3	Plxnb2	Tnfrsf25
Pdgfa	Thbd	Plxnc1	Tnfrsf8
Pdgfra	Thra	Plxnd1	Tnfrsf9
Pdgfrb	Thrap3	Prlhr	Tshr
Pgr	Tnfrsf10b	Prlr	Unc5b
Plat	Tnfrsf11a	Procr	Unc5c
Plaur	Tnfrsf11b	Prokr1	Uts2r
Plgrkt	Tnfrsf12a	Prokr2	Vipr1
Plxnb2	Tnfrsf13c	Ptch1	Vldlr
Prlr	Tnfrsf14	Ptch2	Vtn
Procr	Tnfrsf17	Pth1r	
Prokr1	Tnfrsf18	Pth2r	
Prokr2	Tnfrsf1a	Ptprh	
Ptch1	Tnfrsf1b	Ptprk	
Ptch2	Tnfrsf25	Ptprs	
Pth1r	Tnfrsf8	Ptprz1	
Pth2r	Tnfrsf9	Ret	
Ptprk	Tshr	Robo3	
Ptprs	Unc5b	Ror1	
Ptprz1	Unc5c	Ror2	
Ret	Uts2r	Rorb	
Robo3	Vipr1	Rtn4r	
Ror1	Vldlr	Rtn4rl1	
Ror2	Vtn	Rxfp2	
Rorb		Rxfp4	
Rtn4r		Rxrg	
Rxfp1		Ryr1	
Rxfp2		Ryr2	
Rxrg		Sctr	
Ryr1		Sdc4	
Ryr2		Sfrp1	
Sctr		Sfrp2	
Sdc4		Slc1a5	
Sfrp1		Sorcs3	
Sfrp2		Sort1	
Slc1a5		Sstr1	
Sorcs3		Sstr2	
Sort1		Sstr3	
Sstr1		Sstr4	
Sstr2		Sstr5	
Sstr3		Tek	
Sstr4		Tgfbr1	
Sstr5		Tgfbr2	
Tek		Tgfbr3	
Tgfbr1		Thbd	
Tgfbr2		Thra	
Tgfbr3		Thrap3	
Thbd		Tnfrsf10b	
Thra		Tnfrsf11a	
Thrap3		Tnfrsf11b	
Tnfrsf10b		Tnfrsf12a	
Tnfrsf11a		Tnfrsf13b	
Tnfrsf11b		Tnfrsf13c	
Tnfrsf12a		Tnfrsf14	
Tnfrsf13b		Tnfrsf17	
Tnfrsf13c		Tnfrsf18	
Tnfrsf14		Tnfrsf1a	
Tnfrsf17		Tnfrsf1b	
Tnfrsf18		Tnfrsf25	
Tnfrsf1a		Tnfrsf4	
Tnfrsf1b		Tnfrsf8	
Tnfrsf25		Tnfrsf9	
Tnfrsf4		Trhr	
Tnfrsf8		Tshr	
Tnfrsf9		Unc5b	
Trhr		Unc5c	
Tshr		Uts2r	
Unc5c		Vipr1	
Uts2r		Vipr2	
Vipr1		Vldlr	
Vipr2		Vtn	
Vldlr		Xcr1	
Vtn			
Xcr1			

Receptor mRNAs identified by microarray for motor neurons (MNs; column 1) and by RNA-seq for RGCs (column 3), as defined using the updated ligand-receptor database (modified from Yuzwa et al., 2016). Only included are receptor mRNAs that had expression levels exceeding a cutoff of the top 87% of mRNAs for motor neurons and FPKM of 1 for RGCs. Also shown are receptor mRNAs that overlap between sensory, sympathetic and motor neurons (column 2) or between all four populations of neurons (column 4). The sensory and sympathetic neuron receptor mRNAs are shown in Table 8. The total numbers of receptor mRNAs in each column are indicated.

We obtained similar findings when we analyzed RGCs using a bulk RNA-seq dataset generated from P5–P7 rat RGCs that were cultured for 12 h (Blanco-Suarez et al., 2018). Using an FPKM of 1 as a threshold for expression, we found 320 receptors expressed by RGCs, with 258 of them also expressed by the three peripheral neuron types (Table 10). Modeling of the unidirectional paracrine interactions between the injured nerve and RGCs identified 126 predicted interactions that included all 122 shared sensory and sympathetic neuron interactions (Fig. 7*B*, shared interactions are outlined in purple; Table 9). There were also four interactions that were unique to the RGC model involving CCL9, CXCL1, CXCL2, and CXCL16. Thus, the injured peripheral nerve and in particular the Schwann cells and endoneurial mesenchymal cells are predicted to provide a ligand environment that acts on multiple populations of neurons.

### The communication networks identify mesenchymal-derived ligands that regulate peripheral axon growth

**Figure 8.**
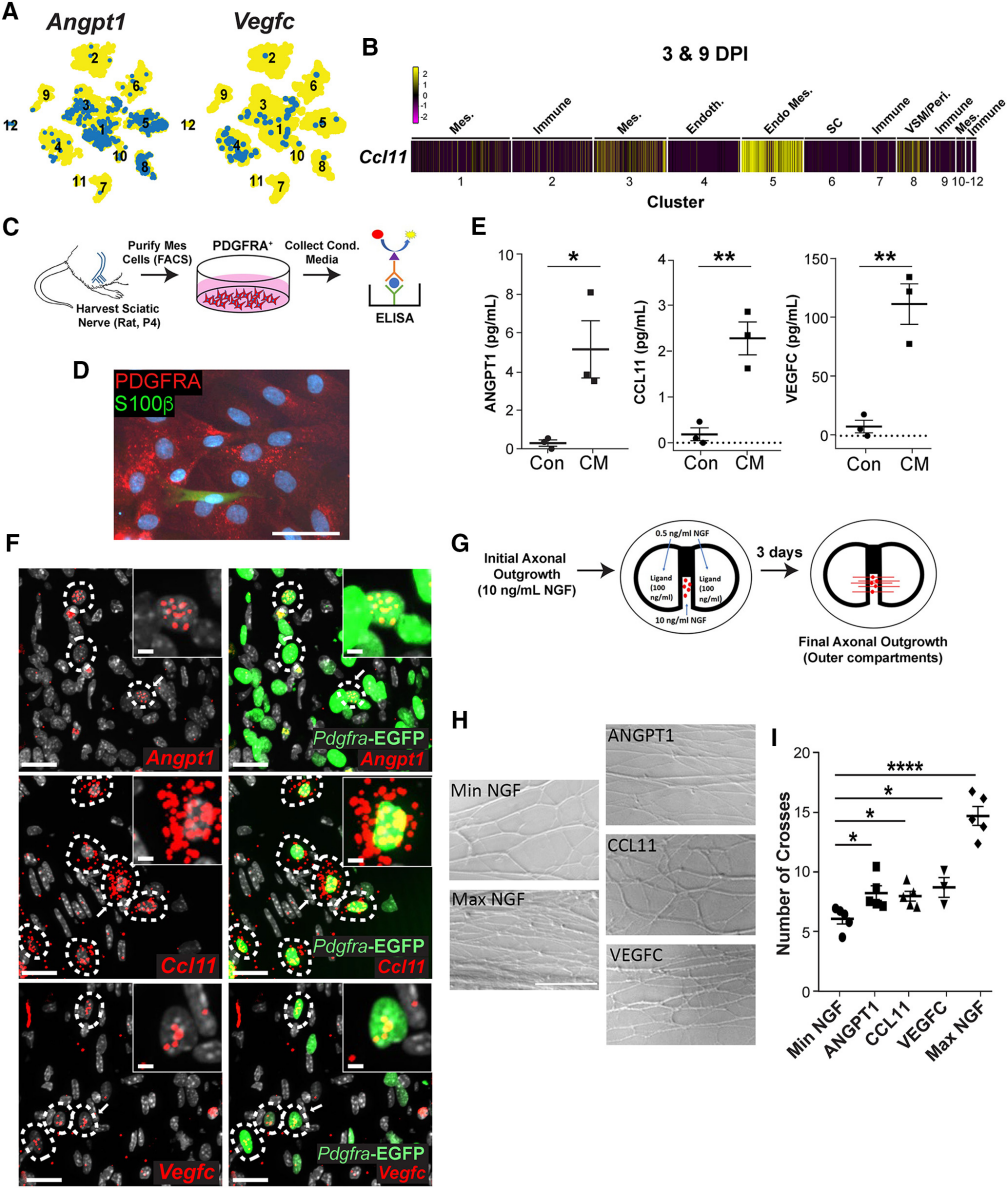
Identification and characterization of ligands expressed in nerve mesenchymal cells that locally promote sympathetic axon growth. ***A***, tSNE gene expression overlays of *Angpt1* and *Vegfc* on the combined 3 and 9 DPI nerve dataset shown in Figure 1*E*. Cells that detectably express the ligand are colored blue, and the numbers correspond to the clusters. ***B***, Heatmap showing expression of *Ccl11* mRNA in single cells within clusters of the combined injured nerve scRNA-seq dataset shown in Figure 1*E*. Each column line represents the level of expression in a single cell. Gene expression represents scaled expression values using Seurat’s scaling function and is color coded as per the adjacent color key, where yellow indicates the highest expression. Cluster numbers are on the bottom, and the cell types in that cluster are annotated on the top. Mes. = mesenchymal, Endoth. = endothelial, Endo Mes. = endoneurial mesenchymal, SC = Schwann cell. ***C–E***, Neonatal (P4) rat sciatic nerve mesenchymal cells were sorted for cell-surface PDGFRα using FACS, cultured in defined growth medium, and medium was collected after 1–4 d of conditioning. ***C***, Schematic of the experiments. ***D***, Representative image of the mesenchymal cell cultures immunostained for PDGFRα and for the Schwann cell protein S100β to indicate the relative purity of the cultures. Scale bar = 50 μm. ***E***, Quantitative ELISA analysis of the nerve mesenchymal cell conditioned medium for ANGPT1, CCL11, and VEGFC (CM). Control growth medium was used as a negative control in each experiment (Con). Shown are the mean ± SEM from three independent experiments; **p < 0.01 (CCL11, *p* = 0.0055; VEGFC, *p* = 0.0045) and *p < 0.05 (ANGPT1, *p* = 0.031), two-tailed unpaired Student’s *t* test. ***F***, Images of the 9-d injured distal sciatic nerve of an adult *Pdgfra^Egfp/+^* mouse analyzed by FISH for *Angpt1*, *Ccl11*, and *Vegfc* mRNAs. Hatched white lines outline EGFP-positive cells (green nuclei) that were also positive for the mRNA of interest (red dots). Also shown is Hoechst 33258 counterstaining (white/gray) to highlight cell nuclei. The arrows indicate cells that are shown at higher magnification in the insets. Scale bars = 20 μm. Scale bars in insets = 8.75 μm. ***G***, Schematic of the compartmented culture axon outgrowth experiments. Neonatal SCG sympathetic neurons were established in compartmented cultures in the presence of 10 ng/ml NGF. When their axons had crossed into the side compartments, the side compartment medium was replaced with medium containing 0.5 ng/ml NGF plus 100 ng/ml of ANGPT1, CCL11, or VEGFC for three additional days. As a positive control, 50 ng/ml NGF was added into side compartments and as a baseline control, axons were maintained in 0.5 ng/ml NGF alone. ***H***, Brightfield images of sympathetic axons in side compartments grown as described in ***G***, located within 1 mm of the furthest extent of axonal outgrowth. Positive control (Max NGF) = 50 ng/ml, negative control (Min NGF) = 0.5 ng/ml. Scale bar = 100 μm. ***I***, Scatter plots showing the density of outgrowth in response to the different ligands. A vertical line was drawn within the farthest 1 mm of axonal outgrowth, and the number of axons crossing the line was quantified. A maximum of eight separate lanes was scored per technical replicate, with three to four technical replicate cultures (i.e., cultures generated from sympathetic ganglia harvested from the same litter) scored per biological replicate (*n* values in plot). Individual points show mean ± SEM of individual biological replicates; *p < 0.05 (*p* = 0.041), ****p < 0.0001, one-way ANOVA with Holm–Sidak’s multiple comparison’s test (*n* = 5 for all treatments except VEGFC, where *n* = 3).

These models predict that, in addition to Schwann cells, nerve mesenchymal cells are key growth factor sources in the injured nerve. To validate this concept, we focused on two predicted endoneurial ligands that have not previously been explored within a nerve context, ANGPT1 and CCL11 (Fig. 8*A*,*B*). We also analyzed VEGFC, which is expressed at a low level in injured nerve mesenchymal cells (Fig. 8*A*), but that had a coreceptor (NPR1) that was validated at the protein level in both sensory and sympathetic neurons.

We first asked whether these three ligands were secreted by nerve mesenchymal cells. To do this, we isolated mesenchymal cells from rat sciatic nerves using antibody-based flow sorting for cell-surface PDGFRα. We cultured and expanded these sorted mesenchymal cells for 2.5–4 weeks, at which point the cultures were comprised of over 90% PDGFRα-positive mesenchymal cells, with the remaining cells being S100β-positive Schwann cells (Fig. 8*C*,*D*). After three further days in culture, we added defined, serum-free medium for 24–96 h, collected this conditioned medium, and performed ELISAs. ANGPT1, CCL11, and VEGFC were all detected in three independent preparations of nerve mesenchymal cell conditioned medium (Fig. 8*E*).

Having confirmed that these ligands were secreted by nerve mesenchymal cells in culture, we asked whether *Angpt1*, *Ccl11*, and *Vegfc* mRNAs were expressed in endoneurial cells of the injured nerve. To do this, we analyzed injured distal nerve sections from the *Pdgfra^Egfp/+^*mice. We resected sciatic nerves and at 9 DPI performed single molecule FISH. *Angpt1* and *Ccl11* mRNAs were detectable in many *Pdgfra-*EGFP-positive mesenchymal cells within the injured nerve endoneurium (Fig. 8*F*). Consistent with the scRNA-seq data, many but not all EGFP-positive cells were positive for these mRNAs (Fig. 8*A*,*B*; 24% and 87% of endoneurial cells express detectable *Angpt1* and *Ccl11* mRNAs, respectively). There were also some *Angpt1* or *Ccl11*-positive cells that were *Pdgfra*-EGFP-negative. For *Angpt1* mRNA these are likely VSM/pericyte cells, while for *Ccl11* mRNA, they could be VSM/pericytes or immune cells (Fig. 8*B*). *Vegfc* mRNA was also detected in scattered endoneurial *Pdgfra*-EGFP-positive cells but consistent with the scRNA-seq analysis (Fig. 8*A*), there were fewer double-labeled cells and the FISH signal was low (Fig. 8*F*).

Having validated their expression, we asked whether ANGPT1, CCL11, or VEGFC could promote growth of peripheral axons. To do this, we used compartmented cultures of neonatal SCG sympathetic neurons (Campenot et al., 1991; Park et al., 2010). In these cultures, cell bodies are physically separated from axons so that ligands can be applied just to the axons, as would occur in the peripheral nerve (Fig. 8*G*). We established sympathetic neurons in these compartmented cultures with 10 ng/ml NGF, their obligate survival factor, in all compartments. Three days later, when axons had crossed into the side compartments, we replaced the side compartment medium with medium containing 0.5 ng/ml NGF plus 100 ng/ml of ANGPT1, CCL11, or VEGFC for three additional days. As a positive control, we added 50 ng/ml NGF into side compartments, and as a baseline control, we maintained axons in 0.5 ng/ml NGF alone. To quantify the density of axonal growth in these compartments, we drew a line perpendicular to the axis of the outgrowth within the furthest 1 mm of outgrowth where axons were maximally defasciculated. This analysis showed that 50 ng/ml NGF caused a robust increase in the density of sympathetic axons relative to the 0.5 ng/ml NGF baseline control (Fig. 8*H*,*I*). Notably, all three mesenchymal ligands modestly but significantly enhanced axonal density, although to a lesser degree than maximal NGF (Fig. 8*H*,*I*). Thus, at least some of the mesenchymally derived ligands predicted in our models were bioactive on sympathetic axons.

## Discussion

In the present study, we have characterized the ligand environment of the uninjured, injured, and developing sciatic nerves using bulk and single-cell transcriptional profiling. We have identified receptor proteins on the surface of two types of peripheral neurons (sensory and sympathetic) and made predictions of ligand-receptor paracrine interactions between the injured nerve and peripheral neurons. We then go on to show, based on these predictions, that mesenchymal cells express factors that are capable of augmenting growth of peripheral axons *in vitro*, indicating that at least some of these ligands may directly contribute to the positive axonal growth environment of the developing and regenerating peripheral nerves.

Peripheral nerves provide a highly supportive environment for axonal growth during development and following injury (Chen et al., 2007; Cattin and Lloyd, 2016; Fledrich et al., 2019) and promote the repair and regeneration of innervated tissues (Kumar and Brockes, 2012; Johnston et al., 2013, 2016; Mahmoud et al., 2015). Many nerve-derived growth factors have already been well studied, including NGF, BDNF, NTF3, GDNF, and cytokines of the LIF/CNTF family (for review, see Terenghi, 1999). These factors are generally assumed to be Schwann cell derived, although macrophages express factors like GAS6 that promote proper function of Schwann cells in the regenerating nerve (Stratton et al., 2018). To identify other important factors that might be involved in providing a supportive peripheral nerve environment, we used a modeling strategy based on transcriptomic analysis and cell-surface mass spectrometry, as has been previously done for embryonic cortical development and digit tip regeneration (Johnston et al., 2016; Yuzwa et al., 2016). However, while bulk transcriptomic profiling was used in these earlier studies, here we added single-cell transcriptional profiling, thereby providing a level of cellular resolution previously not possible for complex tissues. This approach allowed us to define a previously unappreciated role for mesenchymal cells in establishing the nerve paracrine environment, to identify new nerve ligands, and to predict that many nerve ligands will act on both PNS and CNS neurons, thereby potentially providing an explanation for why peripheral nerves can promote growth of CNS axons.

Our study defined many growth factor mRNAs induced in Schwann cells following nerve injury. Some of these encoded previously studied factors like *Artn*, *Bdnf*, *Gdnf*, *Pdgfa*, *Shh*, and *Lif*, while others encoded factors not well studied in this regard, including *Ucn2*, *Fgf5*, and the CNTF-like cytokines *Clcf1* and *Crlf1*. Previous studies have proposed that this ligand induction is part of a unique Schwann cell “repair” phenotype (Jessen and Mirsky, 2019) that is important for axonal regeneration in the case of ligands like BDNF, GDNF, and LIF, and for tissue repair, in the case of PDGFA (Johnston et al., 2016). What is this repair phenotype? Previous work has shown that following injury Schwann cells display altered morphology and gene expression that is thought to be conducive to promoting axonal regeneration (Gomez-Sanchez et al., 2017; Jessen and Mirsky, 2019). Repair Schwann cells have also been reported to have enhanced epithelial to mesenchymal transition gene expression and TGFβ signaling that contributes to the establishment of an invasive, “mesenchymal-like” phenotype (Arthur-Farraj et al., 2017; Clements et al., 2017). Our findings also show that following nerve injury, Schwann cells induce ligand genes that are not expressed at detectable levels in the uninjured neonatal or adult nerves. Moreover, our transcriptional comparisons expand on previous work and show that repair Schwann cells are more similar to neonatal than to adult uninjured nerve Schwann cells but that they are nonetheless distinct. In this regard, our developmental comparison was limited to the neonatal nerve when myelination is ongoing, and it would be interesting to use single-cell transcriptional profiling to see how similar repair Schwann cells are to embryonic nerve Schwann cells before myelination has commenced.

An important finding of this work is that *Pdgfra*-positive mesenchymal cells are a major source of ligands in the developing, adult, and injured nerves and that they express well-characterized nerve growth factors like NGF, HGF, and LIF. Of particular interest is the high ligand expression by endoneurial mesenchymal cells, which are neural crest derived (Joseph et al., 2004) and are scattered throughout the endoneurial space in close apposition to Schwann cells and axons. These endoneurial mesenchymal cells are thus ideally positioned to regulate axon and Schwann cell biology, and, like Schwann cells, they display increased expression of many ligand mRNAs following injury, including well-studied ligands like *Crlf1*, *Ngf*, and *Lif* and less-studied ligands such as *Angpt1*, *Ccl9*, and *Sema7a*. Equally intriguing was the observation that endoneurial cells express many little-studied ligands under homeostatic conditions, including *Adm*, *Bmp7*, *Il33*, *Pthlh*, and *Wnt5a*. Since mesenchymally derived ligands include both well-studied nerve growth factors such as NGF as well as many ligands with unknown roles in nerve biology, it is likely that mesenchymal cells express ligands that are critical components of the regenerative response of the injured nerve and/or the growth program of the developing nerve. Our validation studies indicate that at least some of these endoneurial cell ligands are active on axons, but they might be equally important for other nerve cell types and/or for the tissues they innervate. As one example, PTHLH and nerve innervation are both important for bone homeostasis and repair (Elefteriou et al., 2014; Ansari et al., 2018), and endoneurial cells, which express *Pthlh*, migrate into the injured bone where they directly contribute to bone repair (Carr et al., 2019). As a second example, the vasodilator peptide adrenomedullin (ADM) was previously shown to stimulate cAMP accumulation in endothelial cells and Schwann cells (Dumont et al., 2002), suggesting that endoneurial cell-derived ADM might be important for nerve vasculature and/or Schwann cell biology. As a final example, BMP7 inhibits myelin gene expression in Schwann cells (Liu et al., 2016) and promotes mammalian digit tip regeneration (Yu et al., 2010), suggesting that endoneurial cell-derived BMP7 might play multiple important roles.

Another important finding is that most injured nerve ligands are predicted to act on all three populations of PNS neurons as well as RGCs. In this regard, the nerve could promote axonal growth and regeneration in two somewhat disparate ways. In one model, Schwann cells and endoneurial mesenchymal cells would produce different ligands depending on the axons that they are currently or have previously interacted with, thereby tailoring the nerve environment to the axons that need to grow or regenerate. Support for this model comes from studies showing that denervated Schwann cells of motor versus sensory nerves provide ligands specific to different types of axons (Höke et al., 2006; Brushart et al., 2013). In a second model, during development or following nerve damage Schwann cells and mesenchymal cells could express a broad repertoire of ligands, thereby ensuring that growth of all types of PNS axons would be supported. Our findings support this latter model, since we find broad injury-induced ligand expression and a relatively broad repertoire of receptor expression on four different types of neurons, culminating in many similar predicted paracrine interactions. Such a mechanism would provide maximum flexibility and would explain why peripheral nerve grafts promote regeneration of multiple types of injured CNS neurons, which do not normally project in peripheral nerves. Nonetheless, our findings are still consistent with the finding of differential ligand expression in different nerve subtypes (Höke et al., 2006; Brushart et al., 2013), since we have only defined the ligand landscape in a mixed nerve.

How predictive are these models? Previous studies using bulk transcriptomics and/or cell-surface mass spectroscopy predicted three factors important for embryonic cortical neurogenesis (IFNγ, Neurturin, and GDNF; Yuzwa et al., 2016) and one for oligodendrogenesis (Fractalkine; Voronova et al., 2017), and two factors important for digit tip regeneration (PDGFA and OSM; Johnston et al., 2016). In those studies, the cell of origin for each ligand was identified either by isolating cells or by performing single-cell resolution morphologic approaches. Here, we have instead used single-cell transcriptional profiling to provide the necessary resolution, an approach with much broader applicability. The validity of the resultant models is attested to by our finding that almost all ligands previously shown to be important for peripheral nerve regeneration were independently assigned in our models, including many ligands known to be expressed in Schwann cells. Nonetheless, to ensure the validity of these models, we also examined three ligands that were predicted to be made by nerve mesenchymal cells, ANGPT1, CCL11, and VEGFC. Two of these factors, ANGPT1 and VEGFC, are well-known angiogenesis factors, while the third, CCL11 or eotaxin, is a chemokine involved in eosinophil recruitment (Jose et al., 1994). None of the three has been studied as a positive factor within the context of the injured nerve, although ANGPT1 has been shown to promote growth of cultured sensory neurons (Kosacka et al., 2006), VEGFC promotes growth of developing motor neurons in zebrafish (Kwon et al., 2013), and CCL11 inhibits Schwann cell differentiation (Büttner et al., 2018). Based on our predictive models, we tested these ligands and found that all three (1) were expressed by endoneurial mesenchymal cells in the injured nerve, as shown by both scRNA-seq and FISH analyses; (2) were synthesized and secreted by cultured nerve-derived PDGFRα-positive mesenchymal cells; and (3) enhanced sympathetic axon outgrowth when applied locally in the presence of minimal NGF. While we recognize that additional studies will be required to show that these three ligands are secreted by mesenchymal cells *in vivo*, our data indicate that they are highly expressed following nerve injury, raising the possibility that they are important for nerve repair. In this regard, ANGPT1, CCL11, and VEGFC were not as potent as NGF in promoting growth of sympathetic axons in culture, but they did enhance growth and thus could be important factors for peripheral axon growth in a regenerating nerve context. We therefore feel that our studies validate the predictive value of the modeling approach and provide support for the idea that mesenchymal cells within the nerve are important ligand sources, particularly within the context of nerve repair and regeneration.

The data presented here reinforce the importance of Schwann cells as sources of growth promoting factors and provide evidence that mesenchymal cells also play an important role in determining the ligand environment of the developing and injured nerve. Notably, some well-known nerve regeneration ligands such as GDNF and BDNF were expressed at the highest levels in Schwann cells, while others, such as NGF and HGF, were instead highest in mesenchymal cells. In addition, both cell types express ligands that are not well-characterized as nerve injury ligands, including BTC and UCN2 for Schwann cells, and ANGPT1, CCL11, and VEGFC for mesenchymal cells. While the relative contributions of growth factors from these two cell types to nerve growth and repair remain unknown, our study does highlight mesenchymal cells as a previously overlooked reservoir of growth factors for axon growth and potentially for nerve tissue regeneration, an area we are only now starting to understand (for examples, see Parrinello et al., 2010; Cattin et al., 2015). The data presented here thus provide an important step toward defining nerve paracrine interactions not only with regard to axon growth and peripheral nerve regeneration, but also with regard to the paracrine roles of the nerve during repair and regeneration of target tissues.

## Synthesis

Reviewing Editor: Jeffery Twiss, University of South Carolina Decisions are customarily a result of the Reviewing Editor and the peer reviewers coming together and discussing their recommendations until a consensus is reached. When revisions are invited, a fact-based synthesis statement explaining their decision and outlining what is needed to prepare a revision will be listed below. The following reviewer(s) agreed to reveal their identity: Ahmet Hoke, Moses Chao.

I apologize for the delay in getting this review process completed. Normally I would put together a synthesis statement that incorporates both reviewers comments and an online discussion. The extraordinary circumstances that we are under now has everyone distracted, so I did not get to conduct the online discussion to the extent that would be needed for my usual synthesis. I am appending the direct comments from each reviewer below my summary of their comments and my suggestions for revision.

Both reviewers encouraged resubmission. Both reviewers and the reviewing editor fully recognize the amount of work that went into this study. I think that critiques give clear guidance on points to address for revision. In particular, you should reorganize the text to make sure that the main message of the manuscript is not lost in the single cell RNA data - shortening the manuscript may help as well as being selective on what extended data needs to be included. The statistical analyses are difficult to follow, so more clearly outlining the tests used and rationale for those would help. With the newness of scRNA-Seq, you will do the reader a service by carefully justifying the statistical measures.

For the comments from reviewer 1 on no data showing the ligands play a role in regeneration and issue of Schwann cells outnumbering mesenchymal cells many fold, I think this could be addressed by text changes. Specifically backing off on conclusions and referring to these ligands as potential growth modulators. I fully agree with the reviewer that showing in vitro activity in SCGs is not sufficient to make a conclusion on regeneration. The conclusions would of course be strengthened by in vivo data, but I do not think that is necessary if you can appropriately qualify your conclusions.

For the comment from reviewer 2 on Vegfc, Ccl11 and Angpt1 secretion, I agree with the reviewer that UniProt database designations are not sufficient. Your experimental system could certainly behave differently than the studies that UniProt draws from. Data on secretion in peripheral nerve would certainly strengthen the manuscript. Alternatively, I think that you could suggest this for future studies, acknowledging the limitations of public databases (also I recognize the difficulty in showing secretion in vivo in the PNS, and I think that would unfortunately be the necessary data).

Reviewer 1:

This is an interesting paper that examines potentially secreted ligands that can act on neurons from cells in the injured nerve and concludes that about half of potential ligands are coming from mesenchymal cells. Although potentially a very important observation, the work presented is very preliminary and does not justify the conclusions, including the title. There is absolutely no data shown that these molecules actually play a role in regeneration. Demonstrating that some of the candidate molecules have in vitro activity in SCG neurons is not sufficient to prove that they have a role in regeneration. The authors fail to take into consideration that the number of mesenchymal cells in the distal nerve are dwarfed by Schwann cells and macrophages and that any potentially secreted ligand may or may not have any substantial effect on regeneration.

I think the authors need to be commended for performing this scRNA-Seq analysis as it will be a resource for the whole scientific community. However, the references to regeneration need to be removed from the paper as data is not there to justify such conclusions.

Reviewer 2:

The study covers a topic of interest-transcriptional changes in the sciatic nerve after injury. The goal is to identify ligand-receptor expression in neighboring cells with the goal of identifying molecules involved in regeneration. The manuscript covers the results in a very extensive manner. Unfortunately, the description of the results is very lengthy, unwieldly and drawn out. The results and the conclusions should be edited and shortened to cover the main points in a more succinct manner.

This study is carefully performed with appropriate programs and algorithms (Seurat, Gene Ontology, Panther). The manuscript needs to be strengthened by considering several issues and questions. They include the following questions, which should be addressed and clarified. -

1) The Materials and Methods section has a thorough description of the procedures; the isolation of single cells; the scRNA-seq and computational analysis. However, it is interspersed with commentary and citations about the results. To organize the paper better, the description of the results and Figures should be integrated within the Results section.

2) A major finding is that Vegfc, Ccl11 and Angpt1 are expressed in endoneurial cells after injury. Do the authors have direct evidence the three ligands are secreted? Do they all give a similar receptor signaling response downstream of their receptors? The UniProtKB database is cited as the source of secreted ligands, but the manuscript would be stronger if more data were included.

3) In this regard, the presented facts can be condensed by placing relevant results in separate themes and biological contexts, instead of listings of enriched genes.

4) Several descriptions of the Results should be better explained and more comprehensible. For example, “This analysis identified seven Sox10-positive clusters containing 5331 Schwann cells” does not convey the significance of this finding.

5) The effect of injury upon ligand mRNAs needs to be clarified, as it is unclear if the levels of Ccl1 are greatly affected (Fig. 4G). Also, Ccl11 appears to be expressed in many cell types-Schwann, vascular, immune cells (Fig. 8). The cell specificity of ligand expression should be emphasized from the analysis.
