# Supplementary figures and images for: Peripheral Nerve Single-Cell Analysis Identifies Mesenchymal Ligands that Promote Axonal Growth
Source: eNeuro. 2020 Jun 11;7(3):ENEURO.0066-20.2020. doi: 10.1523/ENEURO.0066-20.2020 (PMC7294463; doi:10.1523/ENEURO.0066-20.2020)

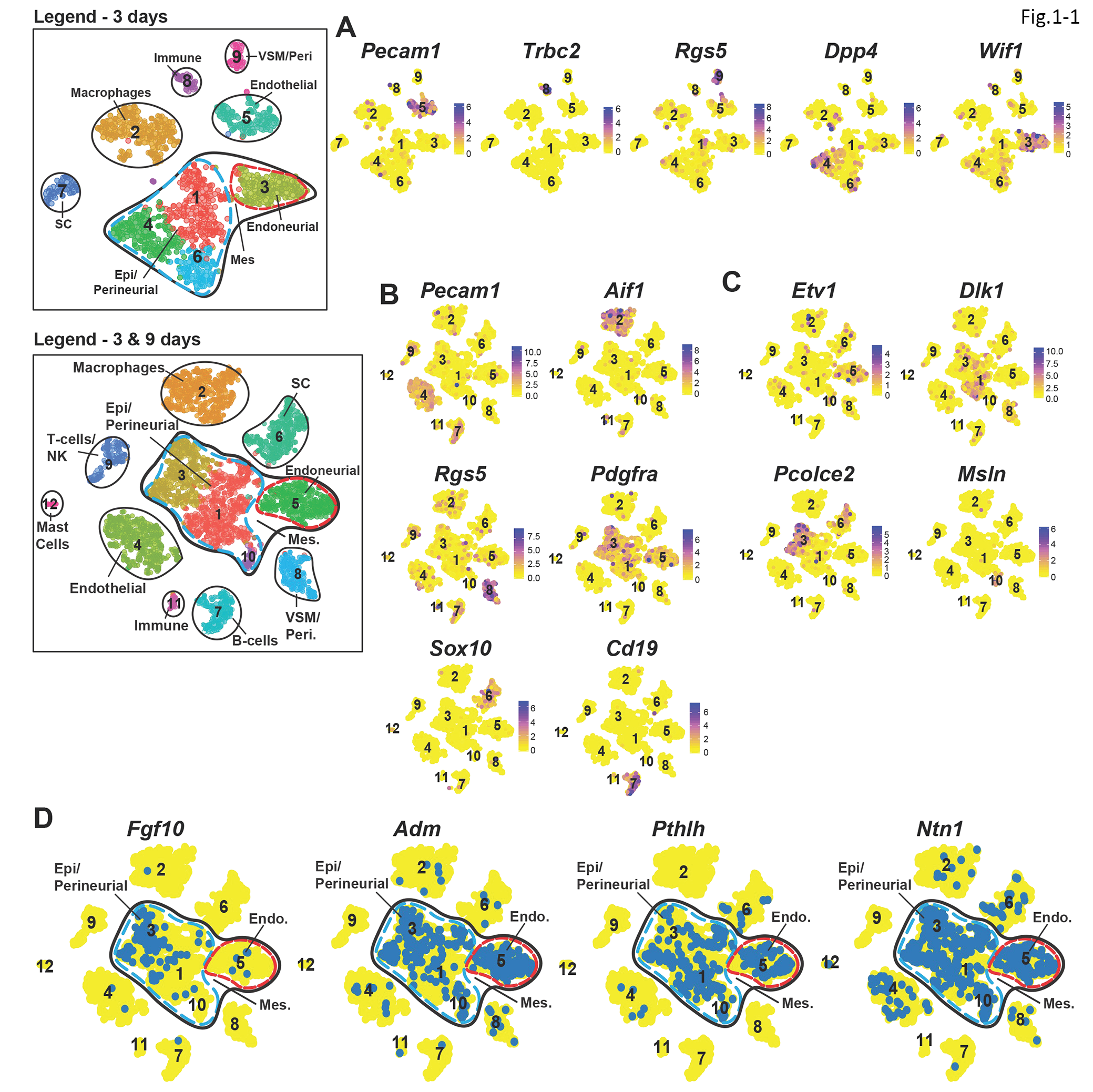

Supplement: Extended Data Figure 1-1 — Characterization of the 3- and 9-d injured sciatic nerve scRNA-seq datasets. A, t-SNE gene expression overlays on the 3 DPI total cell dataset (shown in Fig. 1C and in the adjacent legend) for the endothelial cell marker Pecam1, the immune cell marker Trbc2, the VSM/pericyte cell marker Rgs5, and the Pdgfra-positive mesenchymal epineurial and endoneurial cell markers Dpp4 and Wif1. Relative transcript expression levels are color coded as per the adjacent color keys and numbers correspond to clusters. B, t-SNE gene expression overlays on the combined 3 and 9 DPI total cell datasets (shown in Figure 1E and in the adjacent legend) for the endothelial cell marker Pecam1, the immune cell marker Aif1, the VSM/pericyte cell marker Rgs5, the mesenchymal marker Pdgfra, the Schwann cell marker Sox10, and the B cell marker Cd19. Relative transcript expression levels are color coded as per the adjacent color keys and numbers correspond to clusters. C, t-SNE gene expression overlays on the combined 3 and 9 DPI total cell dataset (shown in Fig. 1E and in the adjacent legend) for markers for the different types of Pdgfra-positive mesenchymal cells, including Etv1-positive endoneurial cells, Pcolce2-positive epineurial cells, Msln-positive perineurial cells, and Dlk1-positive differentiating mesenchymal cells. Relative transcript expression levels are color coded as per the adjacent color keys and numbers correspond to clusters. D, t-SNE gene expression overlays of the combined 3 and 9 DPI total cell dataset for Fgf10, Adm, Pthlh, and Ntn1. Cells that detectably express the ligand are colored blue and the numbers correspond to the clusters. Specific cell types are circled and annotated, including mesenchymal (Mes.), endoneurial (Endo.), and epineurial/perineurial (Epi/Perineurial) cells. Download Figure 1-1, TIF file [file enu-eN-NWR-0066-20-s02.tif]

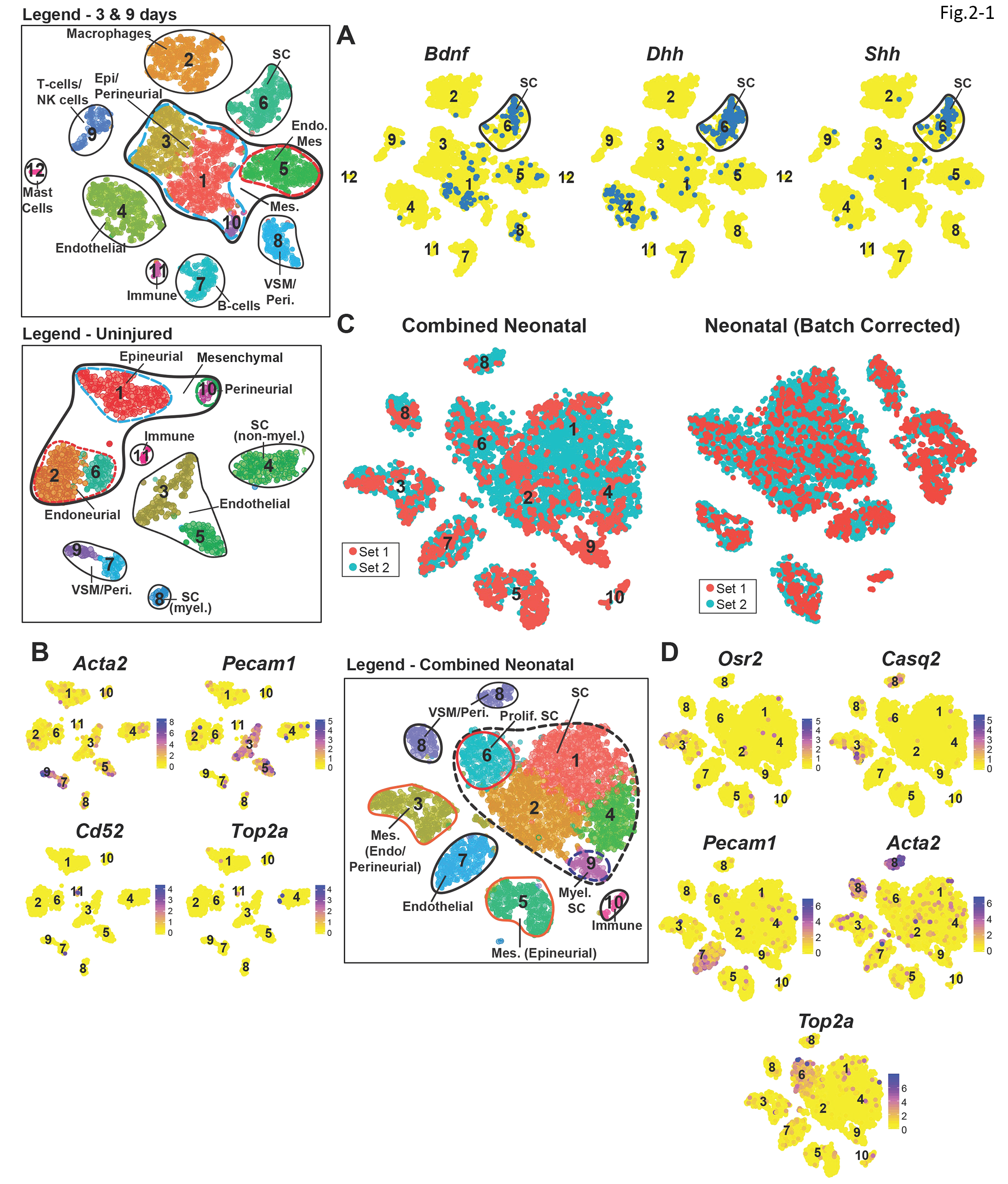

Supplement: Extended Data Figure 2-1 — Characterization of the uninjured and neonatal sciatic nerve scRNA-seq datasets. A, t-SNE gene expression overlays of the combined 3 and 9 DPI total cell dataset (shown in Fig. 1E and the adjacent legend) for Bdnf, Dhh, and Shh. Cells that detectably express the ligand are colored blue and the numbers correspond to the clusters. Schwann cells are circled and annotated (SC). B, t-SNE gene expression overlays on the uninjured sciatic nerve total cell dataset (shown in Fig. 2C and in the adjacent legend) for the VSM/pericyte cell marker Acta2, the endothelial cell marker Pecam1, the immune cell marker Cd52, and the proliferating cell marker Top2a. Relative transcript expression levels are color coded as per the adjacent color keys and numbers correspond to clusters. C, t-SNE cluster visualization of neonatal sciatic nerve single-cell transcriptomes (as in Fig. 2E and the adjacent legend) showing dataset of origin. Set 1 (red) refers to the neonatal nerve cells isolated by FACS and Set 2 (blue) to the neonatal nerve cells isolated by treatment with the myelin removal beads. The right t-SNE cluster visualization indicates the datasets of origin following Harmony data integration batch correction of the combined datasets. D, t-SNE gene expression overlays on the neonatal sciatic nerve total cell dataset (shown in Fig. 2E and in the adjacent legend) for Osr2, which marks endoneurial mesenchymal cells, Casq2, which is expressed in perineurial cells, the endothelial cell marker Pecam1, the VSM/pericyte cell marker Acta2, and the proliferating cell marker Top2a. Relative transcript expression levels are color coded as per the adjacent color keys and numbers correspond to clusters. Download Figure 2-1, TIF file. [file enu-eN-NWR-0066-20-s03.tif]

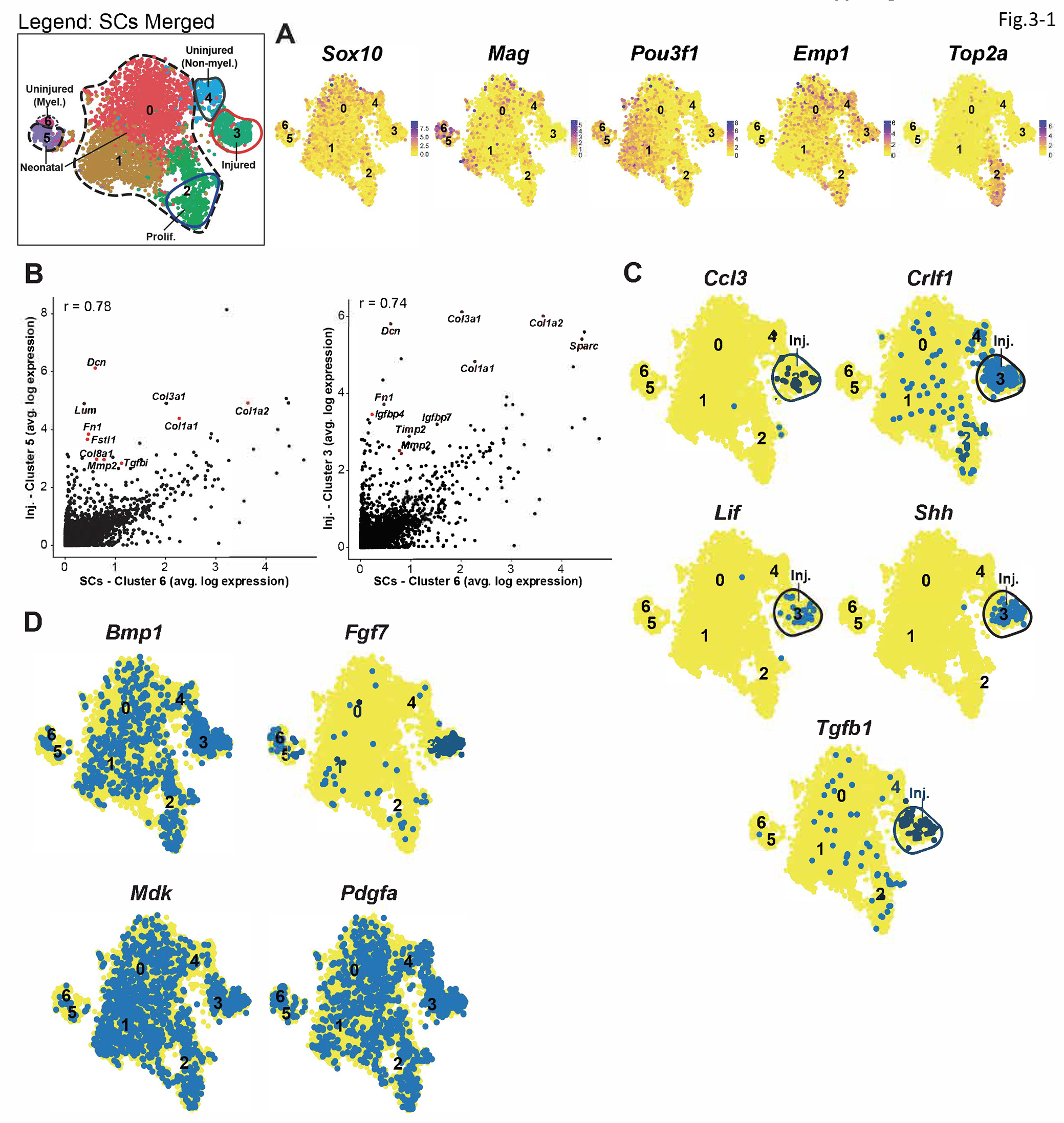

Supplement: Extended Data Figure 3-1 — Characterization of the combined Schwann cell sciatic nerve scRNA-seq dataset. A, t-SNE gene expression overlays on the combined and batch-corrected neonatal, injured adult and uninjured adult Schwann cell data (shown in Fig. 3A and the adjacent legend) for Sox10, the myelination gene Mag, the pre-myelinating Schwann cell marker Pou3f1, the non-myelinating Schwann cell gene Emp1, and the proliferation marker Top2a. Relative transcript expression levels are color coded as per the adjacent color keys and numbers correspond to clusters. B, Plots show correlation analyses of average transcript expression in the in the combined injured nerve dataset (Fig. 1E) showing Schwann cell cluster 6 compared to endoneurial cell cluster 5 (left plot) and to epineurial cell cluster 3 (right plot). Outlier transcripts expressed in the mesenchymal cell clusters are highlighted red and labelled. C, D, t-SNE gene expression overlays of the combined Schwann cell data (shown in Fig. 3A and the adjacent legend) for Ccl3, Crlf1, Lif, Shh, and Tgfb1 (C) and Bmp1, Fgf7, Mdk, and Pdgfa (D). Cells that detectably express the ligand are colored blue and the numbers correspond to the clusters. Injured Schwann cell cluster 3 is circled and annotated (C, Inj.). Download Figure 3-1, TIF file. [file enu-eN-NWR-0066-20-s04.tif]

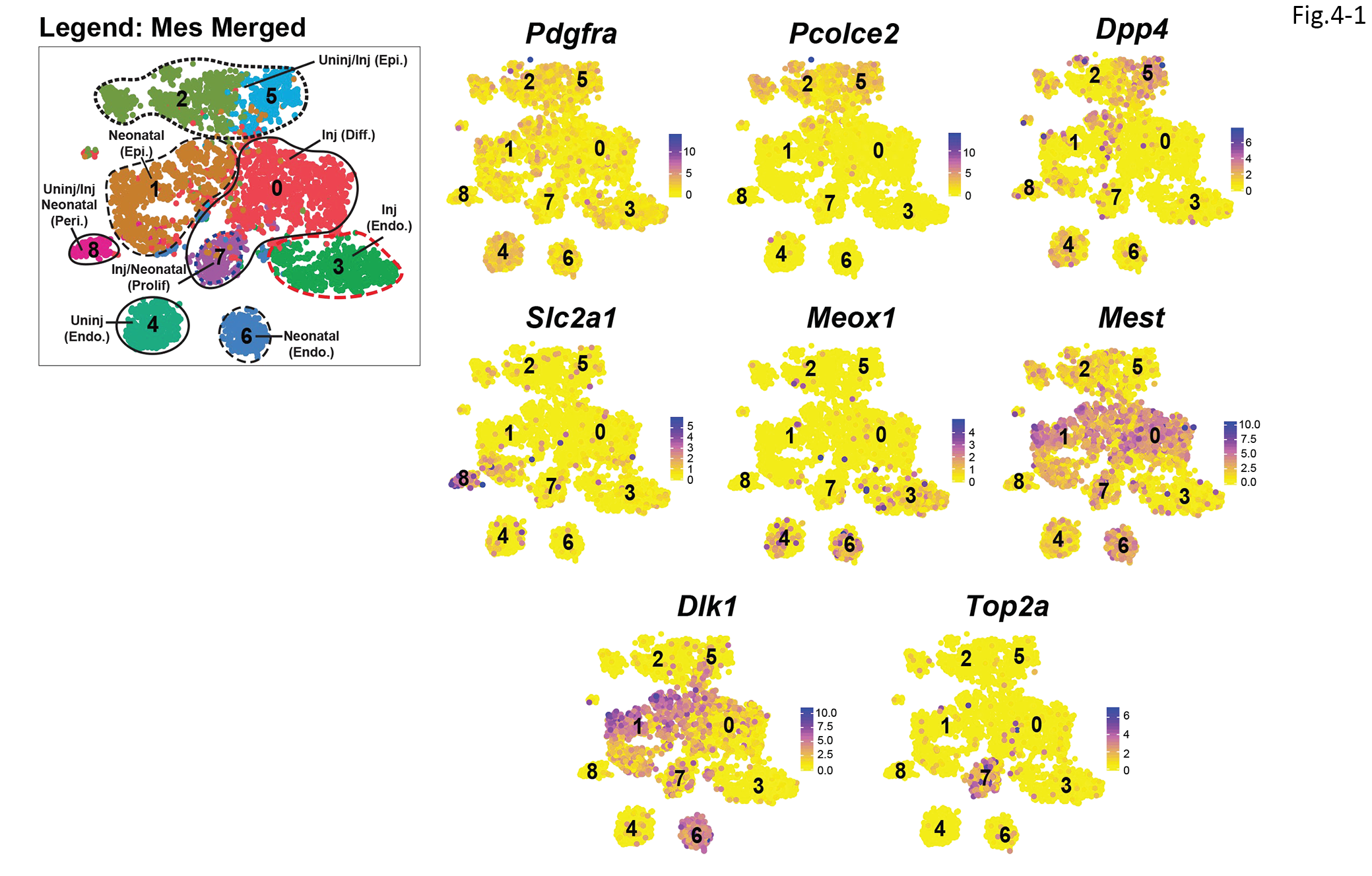

Supplement: Extended Data Figure 4-1 — Characterization of the combined Pdgfra-positive mesenchymal cell sciatic nerve scRNA-seq dataset. t-SNE gene expression overlays on the combined and batch-corrected neonatal, injured adult and uninjured adult Pdgfra-positive mesenchymal cell data (shown in Fig. 4A and the adjacent legend) for Pdgfra, the epineurial markers Pcolce2 and Dpp4, the perineurial gene Slc2a1, the endoneurial gene Meox1, the differentiating injured cell genes Mest and Dlk1, and the proliferation gene Top2a. Relative transcript expression levels are color coded as per the adjacent color keys and numbers correspond to clusters. Download Figure 4-1, TIF file. [file enu-eN-NWR-0066-20-s05.tif]

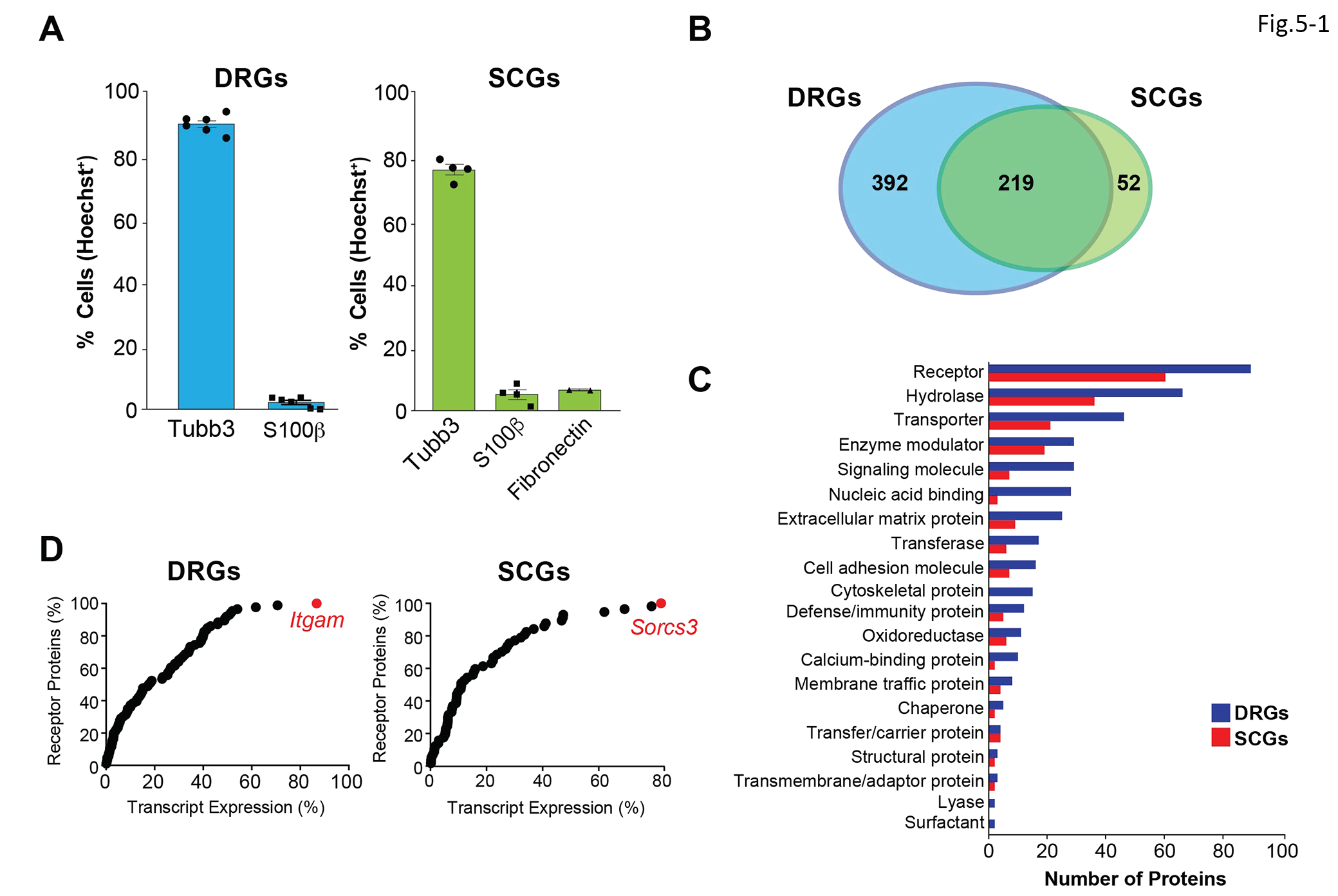

Supplement: Extended Data Figure 5-1 — Identification of cell-surface proteins on sensory and sympathetic neurons. A, Bar graphs showing the percentage of cells expressing the neuronal protein βIII-tubulin (Tubb3), the Schwann cell protein S100β, or the fibroblast protein Fibronectin in cultures of DRG sensory neurons or SCG sympathetic neurons as shown in Figure 5A. The total number of cells in the cultures was determined by counterstaining with Hoechst 33258. Values: mean ± SEM, n = 6 for DRGs, n = 4 for SCGs except for cultures immunostained for Fibronectin where n = 2. B, Venn diagram showing the overlap of cell-surface proteins detected by mass spectrometry in sensory neurons and sympathetic neurons. All proteins included were annotated by the terms “cell membrane” and/or “secreted” by the UniProtKB database (http://uniprot.org). C, Bar graphs showing classification of the proteins detected by cell-surface capture mass spectrometry on sensory neurons (DRGs, blue) and sympathetic neurons (SCGs, red). Proteins were classified as receptors based on the ligand-receptor database, GO terms in the PANTHER classification system, as well as by manual curation, and were further classified into receptor types as shown in Figure 5B. The remainder of the graph includes proteins classified using PANTHER (http://pantherdb.org). D, Graphs showing the distribution of proteins detected by cell-surface capture mass-spectrometry relative to their transcript expression levels (based on microarray analyses described in the text) in sensory neurons (DRGs, left) and sympathetic neurons (SCGs, right). The cutoffs used to define receptor expression in the microarray data were based on the receptors detected by mass spectrometry analysis that had the lowest levels of mRNA expression. This was Itgam for sensory neurons (DRGs) and Sorcs3 for sympathetic neurons (SCGs, shown in red). Download Figure 5-1, TIF file. [file enu-eN-NWR-0066-20-s06.tif]

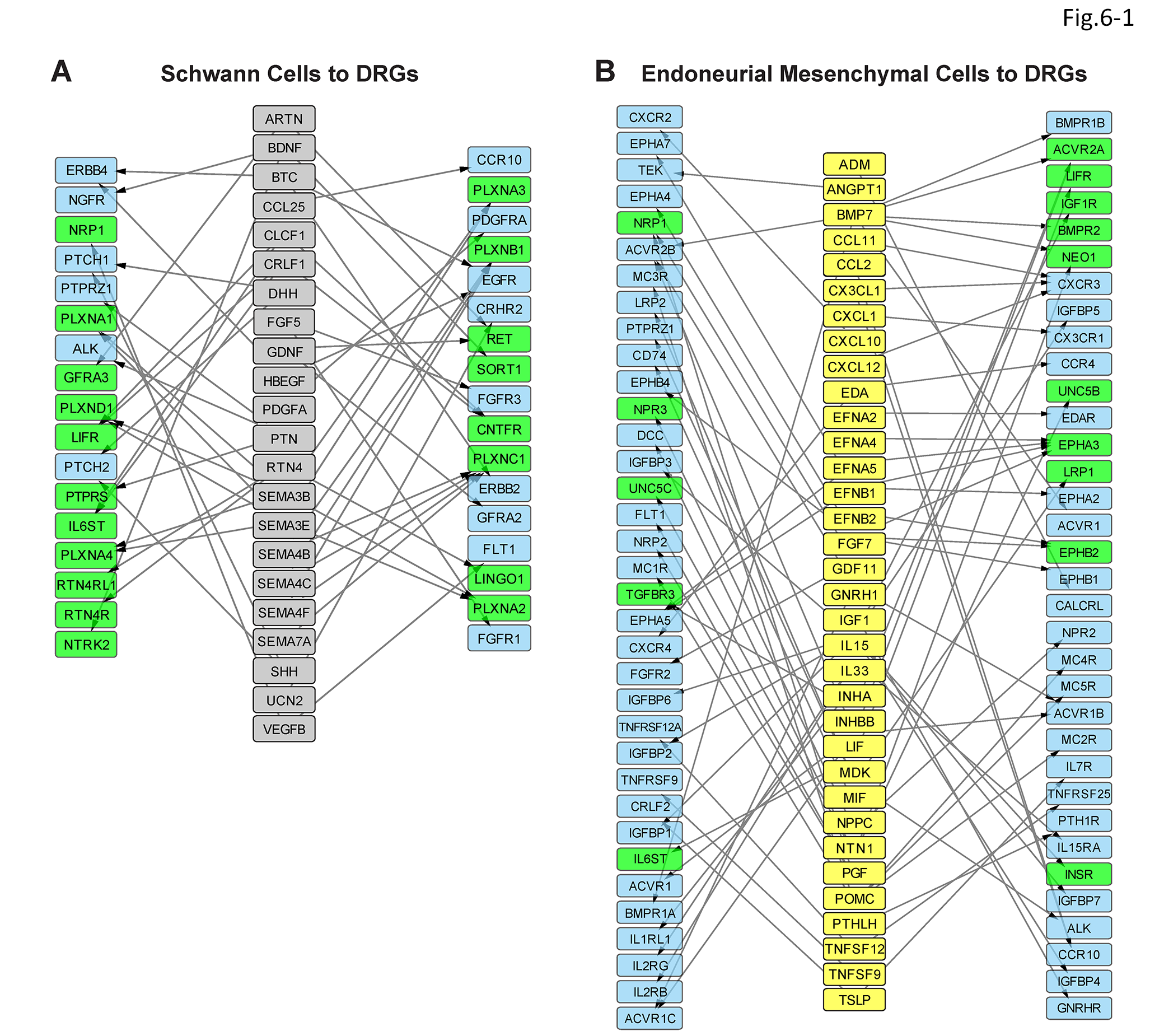

Supplement: Extended Data Figure 6-1 — Predicted unidirectional ligand-receptor interactions between injured sciatic nerve Schwann cells or endoneurial mesenchymal cells and sensory neurons. Models showing predicted unidirectional interactions between the ligands most highly expressed by injured nerve Schwann cells (A) or endoneurial mesenchymal cells (B) and their receptors on cultured sensory neurons (DRGs). Ligands are shown in the central columns in A, B and are color coded as in Figure 5 (Schwann cell ligands in grey and endoneurial mesenchymal cell ligands in yellow). Receptors are shown on either side of the ligand column and also include coreceptors that are well-characterized components of receptor complexes. Receptors that were observed at both the transcriptomic and proteomic levels are colored green while those defined only at the transcriptomic level are colored blue. Arrows indicate directionality of interactions. Note that many ligands interact with multiple receptors and, conversely, that multiple ligands are sometimes predicted to share receptors. Download Figure 6-1, TIF file. [file enu-eN-NWR-0066-20-s07.tif]
